# Zwitterionic [Gd-(DOTA)] MRI Probes: Influence of Sulfobetaine Linker Length on Relaxivity

**DOI:** 10.1021/acsomega.6c00875

**Published:** 2026-04-16

**Authors:** Lennart F. V. Spickschen, David S. Pusztai, Michael G. Kaul, Verena R. Schulze, Marie Oest, Aleksander J. Swierzewski, Neus Feliu, Markus Fischer, Theophraste Lescot, Marc-André Fortin, John V. Frangioni, Wolfgang Maison

**Affiliations:** a Department of Chemistry, Institute of Pharmacy, University of Hamburg, Bundesstrasse 45, Hamburg 20146, Germany; b Department of Diagnostic and Interventional Radiology and Nuclear Medicine, Center of Radiology and Endoscopy, University Medical Center Hamburg-Eppendorf, Hamburg 20246, Germany; c 28428 Fraunhofer Institute for Applied Polymer Research IAP, Center for Applied Nanotechnology CAN, Grindelallee 117, Hamburg 20146, Germany; d Hamburg School of Food Science, Institute of Food Chemistry, University of Hamburg, Grindelallee 117, Hamburg 20146, Germany; e Axe Oncologie, Centre de Recherche du CHU de Québec-Université Laval, 2705, boul. Laurier, Québec, QC G1V 4G2, Canada; f Centre de Recherche sur le Cancer (CRC), 177453 de l’Université Laval, 9 Rue McMahon, Québec, QC G1R 3S3, Canada; g Département de Génie des Mines, de la Métallurgie et des Matériaux, Université Laval, Québec, QC G1V 0A6, Canada; h Curadel Pharma, 28120 Hunters Ridge Blvd, Suites 6-7, Bonita Springs, FL, Massachusetts 34135, United States

## Abstract

Macrocyclic chelators are a development platform for new Gd­(III)-based contrast agents (GBCAs) for magnetic resonance imaging. Zwitterionic modification of these Gd­(III)-complexes has been reported to improve their solubility, relaxivity, and stability, resulting in enhanced imaging performance and safety due to lower doses and reduced *in vivo* retention. Herein, the synthesis of zwitterionic Gd­(III)-complexes based on 1,4,7,10-tetraazacyclododecane-*N*,*N*′,*N*″,*N*‴-tetraacetic acid (DOTA) bearing sulfobetaine groups is reported. The sulfobetaine carbon spacer length (CSL) was varied, and the relaxivity of the complexes was evaluated at 1.4 and 7 T to assess the influence of charge separation on relaxivity. Measurements were performed in water and saline to probe potential salt effects of the different zwitterions. All GBCAs showed relatively high relaxivities compared to similar nonzwitterionic [Gd-(DOTA)]-derivatives. The highest value in water (*r*_1_ = 8.33 ± 0.14 mm^–1^ s^–1^) was observed for **7b** (CSL = two methylene groups). The impact of CSL on relaxivity in water was moderate. In saline, relaxivity decreased by 13.5–19.8% for compounds **7a**, **7b**, and **7d** (CSL = 1, 2, and 4 methylene groups). Only derivative **7c** (CSL = 3 methylene groups) had a slightly increased relaxivity in saline (+4.2%). These findings indicate that the CSL of sulfobetaine groups has only a minor effect on the relaxivity of Gd­(III) complexes in deionized water. However, it has a significant effect on relaxivity in saline and can thus influence the performance under physiological conditions as evaluated with a first *in vivo* application in mice. A CSL of three methylene groups is thus optimal for the design of zwitterionic GBCAs.

## Graphical abstract

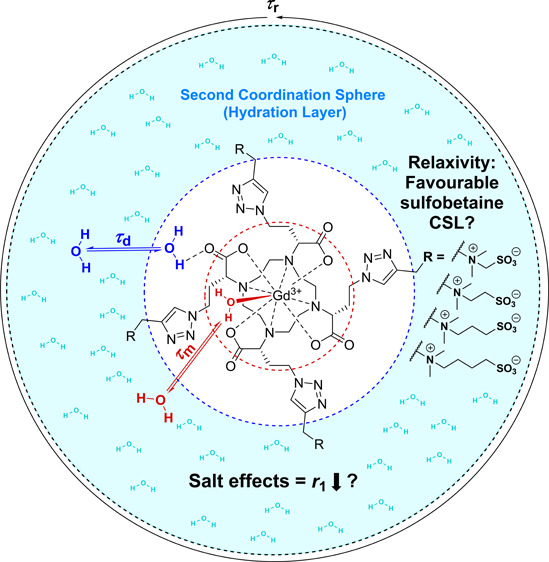

## Introduction

With approximately 700 million administered doses worldwide since introduction, Gd­(III)-based contrast agents (GBCAs) are an important component of clinical diagnostics via contrast-enhanced magnetic resonance imaging (CE-MRI).^1^ They have a good safety profile and acute adverse reactions connected to the clinical use of GBCAs are rare.^2^ Although GBCAs have been used successfully in clinical practice for decades, unwanted physiological effects connected to their application have recently been reported. This applies particularly to GBCAs based on acyclic gadolinium chelators such as gadopentetic acid, which in some cases led to the accumulation of gadolinium in the brain of patients or caused nephrogenic systemic fibrosis.^1,3–6^ This is most likely due to the release of (toxic) Gd­(III) from the complexes. The use of GBCAs based on acyclic chelators has therefore been restricted by health authorities.^7^ Macrocyclic chelators such as DOTA, on the other hand, have very high *in vivo* stability and therefore do not release Gd­(III) in patients.^8,9^ However, unwanted retention of gadolinium in patients and animals has also been detected after administration of GBCAs based on macrocyclic chelators,^10^ which is most likely due to the accumulation of intact gadolinium complexes particularly in the kidneys of patients.^11,12^

An obvious solution to the problem of *in vivo* gadolinium retention is to replace the toxic Gd­(III) with less toxic metal cations.^13^ Iron- ^14,15^ or manganese-based^16,17^ contrast agents are attractive choices in this context. However, to date, most of these contrast agents are either not sufficiently effective or lack *in vivo* stability. In consequence, GBCAs are still the most frequently used contrast agents used clinically for CE-MRI.

To minimize the problems mentioned above, GBCAs should 1. be administered in the lowest possible doses. 2. suitable GBCAs must be very stable *in vivo* to avoid release of toxic Gd­(III) and 3. they should ideally not be retained in patients.^18^ To address these points in the development of new GBCAs, the following should be considered: The dosage of GBCAs depends on their efficiency in generating contrast, which in turn is determined by different physicochemical properties that are summarized in the relaxivity value (*r*_1_ = increase in the relaxation rate *T* of water by a 1 mm GBCA solution). GBCAs are primarily used in *T*_1_-weighted MRI, but they enhance both the longitudinal relaxation rate 1/*T*_1_ and the transverse relaxation rate 1/*T*_2_.^19,20^ *r*_1_ is influenced by external factors such as temperature, salinity of the medium and the magnetic field strength. On the other hand, the chemical structure of GBCAs influences *r*_1_, particularly through the number of Gd-coordinated water molecules (*q*) (inner-sphere water), the molecular size or shape (defines the tumbling rate), and the bonding and ordering of surrounding water molecules (second-sphere water).^21^ Increasing the number of Gd-coordinated water molecules (*q*) often results in a loss of both kinetic and thermodynamic stability of the complex.^3,9^ Many of the clinically used macrocyclic chelators (DOTA and derivatives) are octadentate and allow the coordination of only one water molecule (*q* = 1), which is typically associated with only moderate values of *r*_1_.^8^ A notable exception is gadopiclenol, which is based on a pyclen-scaffold. It coordinates two inner-sphere water molecules (*q* = 2) and has a much higher *r*_1_ value than most DOTA derivatives.^22^ The size of GBCAs cannot be increased arbitrarily without adversely altering their pharmacokinetics. In addition, other factors such as charge, hydrophilicity and complex geometry can also have a considerable influence on *r*_1_ for GBCAs based on DOTA^23^ and similar derivatives.^24^ Of particular interest is the decoration of macrocyclic chelators with hydrophilic groups. These can ensure tight binding of water molecules via hydrogen bonds and/or electrostatic interactions in a second coordination sphere around the metal complex. This ordered water layer near the metal complex can therefore have a significant influence on relaxation.^25^ In addition, the binding of water molecules by polar groups increases the hydrodynamic diameter of the GBCA, which also leads to higher values of *r*_1_. Suitable polar groups are hydroxyl groups^26^ or zwitterions,^27^ such as sulfobetaines.^23^ The latter bind water particularly strong^28^ and lead to relatively long residence times of water molecules near the zwitterionic groups.^29^ The ordered water layer around zwitterionic groups has led to numerous applications of polyzwitterionic materials, particularly in the field of materials science.^30^ These materials are known to prevent bacteria and protein adhesion,^31^ suppress ice formation,^32^ and possess excellent lubricating properties. Furthermore, the structure and properties of zwitterions are often dependent on the salt concentration and temperature of the surrounding media.^33,34^ Work in materials science also suggests that the type of zwitterions^29^ and the distance between the positively and negatively charged groups (or their CSL)^35^ influence the hydration of zwitterions.^35^ These factors may thus also impact the properties of zwitterionic GBCAs.

It has been shown previously that the decoration with zwitterionic groups leads to an increase in relaxivity values (*r*_1_) of gadoteric acid and pyclen-based Gd-complexes.^23,27,36^ This study focuses on the influence of the CSL of sulfobetaines on the relaxivity of gadoteric acid derivatives. Furthermore, the influence of physiological salt concentrations on the *r*_1_ values of zwitterionic GBCAs is investigated.

## Results and Discussion

### Synthesis

A set of zwitterionic DOTA-derivatives **7a**–**d** was assembled via copper-catalyzed azide–alkyne cycloaddition (CuAAC) of DOTAZA **5**. Compound **7c** (CSL = 3) of this series has been reported before,^23,37^ the other close structural analogues **7a**, **7b** and **7d** (CSL = 1, 2, and 4 methylene groups) are new compounds. The synthesis is shown in Scheme 1 and starts from (*S*)-4-amino-2-hydroxy butanoic acid **1**. It has been shortened slightly compared to earlier protocols.^37,38^ Conversion to (*S*)-4-azido-2-hydroxy butanoic acid **2** was achieved in first experiments involving a diazo transfer to the primary amine **1**, as described by Nudelman et al.^39,40^ This transformation requires the *in situ* generation of trifluoromethanesulfonyl azide (TfN_3_) involving an excess of NaN_3_, trifluoromethanesulfonic anhydride (Tf_2_O) and dichloromethane as the solvent, according to the one-pot reaction procedure reported by Alper et al.^39^ Following the diazo transfer, an aqueous acidic workup at pH 1 was performed. While this procedure gave (*S*)-4-azido-2-hydroxy butanoic acid **2** in 91% yield and was reproducible in our hands, it poses significant safety risks. TfN_3_ is explosive and although it is *in situ* generation is common practice, the combination of excess NaN_3_ and unreacted Tf_2_O during the addition of CuSO_4_ is dangerous. Under these conditions, formation of copper azide [Cu­(N_3_)_2_], a shock-sensitive explosive species, may occur. Moreover, the acidic workup may lead to the release of hydrazoic acid (HN_3_), which is toxic, volatile and explosive. Another serious concern is the use of dichloromethane as solvent, which is known to react with NaN_3_ to form diazidomethane, a highly explosive and shock-sensitive compound.^41^ A safer alternative route was therefore developed. It involves the preparation of TfN_3_ in toluene, as previously described by Titz et al.,^42^ followed by its immediate reaction with an excess of the primary amine. This approach avoids the simultaneous presence of CuSO_4_ and NaN_3_, as well as the existence of NaN_3_ or unreacted TfN_3_ during the aqueous workup under acidic conditions. Under these optimized conditions, the formation of hazardous byproducts, such as copper azide and hydrazoic azide is effectively prevented at all stages of the reaction. Notably, the isolated yield of (*S*)-4-azido-2-hydroxy-butanoic acid **2** was high (88%, Scheme 1).

**Scheme 1:**
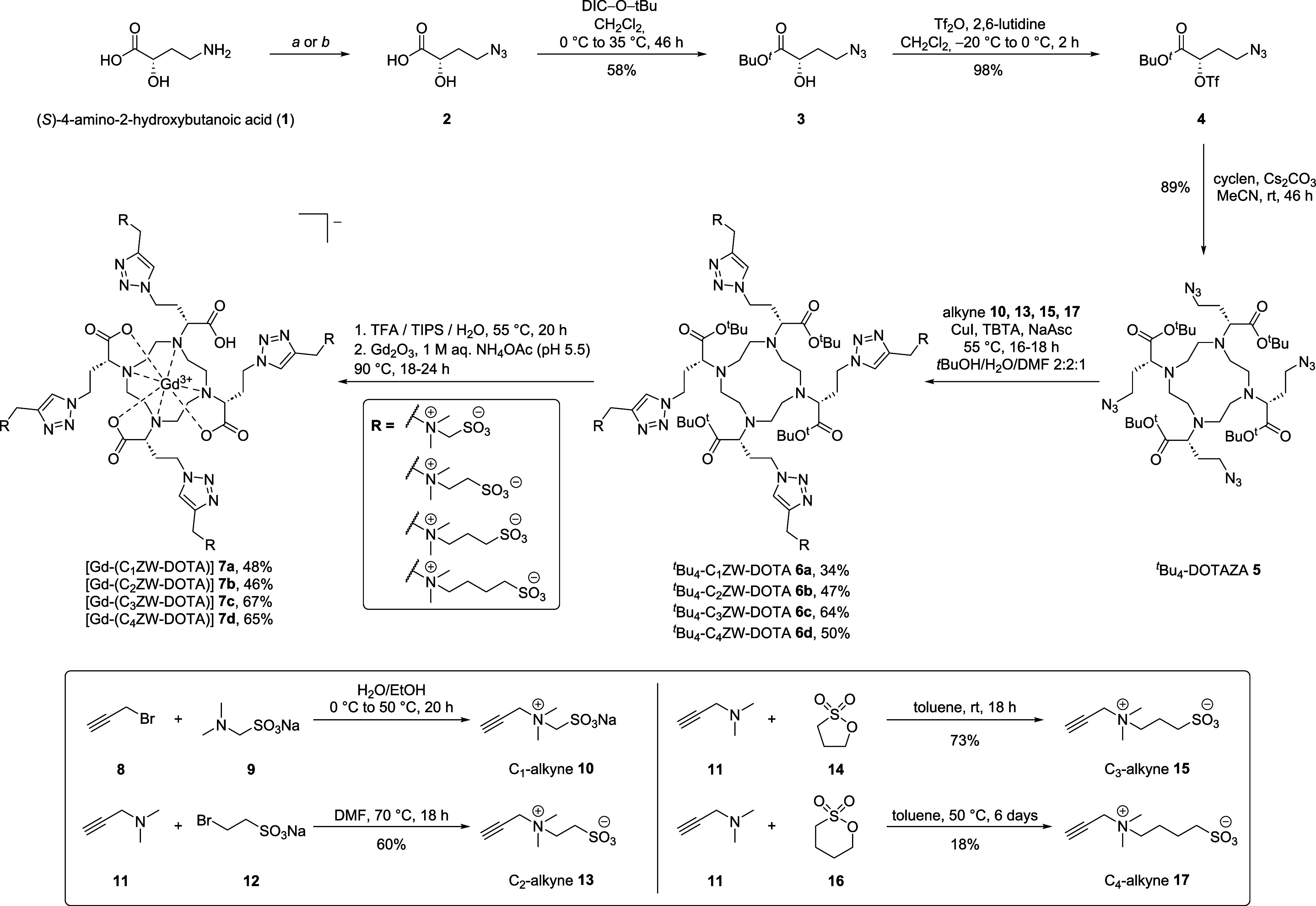
Synthesis of ^*t*^Bu_4_-DOTAZA **5**, Zwitterionic Alkynes **10**, **13**, **15**, **17**, CuAAC and Complexation to Zwitterionic Gadolinium Complexes **7a**-**7d** with a CSL of One to Four Methylene Groups

Subsequently, the carboxylic acid was converted into the corresponding *tert*-butyl ester by reaction with *O*-*tert*-butyl-*N*,*N*′-diisopropylisourea (DIU-O^*t*^Bu) in dichloromethane to give **3** in 58% yield. The α-hydroxy group was treated with Tf_2_O and 2,6-lutidine to give (*S*)-triflate **4** in 98% yield. This sequence gives triflate **4** in three steps, which is significantly shorter than alternative routes.^37^ Subsequent nucleophilic substitution of (*S*)-triflate **4** with cyclen in the presence of Cs_2_CO_3_ gave the *tert*-butyl-protected chelator ^*t*^Bu_4_-DOTAZA **5** in 89% yield.

A series of zwitterionic alkynes with different CSL was prepared to allow the modification of ^*t*^Bu_4_-DOTAZA **5** *via* CuAAC.^43,44^ The resulting zwitterionic chelators **6a**-**6d** were obtained in reasonable yields. It is notable that the zwitterionic *tert*-butyl-protected DOTA chelators form stable sodium complexes.^37^ The sodium ions were introduced with the 0.60 equiv of sodium ascorbate used in the CuAAC reaction. In the ^1^H NMR spectra of compounds **6a**-**6d**, the coexistence of the chelator and its sodium complex led to duplicated or broadened signals. Although not investigated in detail, the zwitterionic DOTA derivatives **6a**-**6d** were significantly more stable under acidic conditions compared to other DOTA-derivatives.^37^ This stabilizing effect of zwitterions has been observed for other chelators too.^45^ It leads also to a remarkable stability of the *tert*-butyl esters during acidic deprotection. While nonzwitterionic *tert*-butyl-protected DOTA derivatives can be cleaved at room temperature with 50% trifluoroacetic acid (TFA) in dichloromethane, the zwitterionic derivatives required significantly harsher conditions. No deprotection, not even partial cleavage of a single ester group, was observed with TFA/CH_2_Cl_2_ mixtures, neat TFA or 1 m aqueous HCl at room temperature. Instead, a mixture of 1% triisopropylsilane (TIPS) and 1% H_2_O in TFA at 55 °C for 20 h was required to achieve full deprotection of all four *tert*-butyl esters to give the deprotected chelators. Alternatively, rapid and efficient deprotection was achieved using concentrated aqueous HCl at 40 °C for 20 min. Notably, no degradation of the chelators was detected and the zwitterionic chelators were obtained in quantitative yields with both deprotection protocols. The complexation with Gd_2_O_3_ was performed at 90 °C in aqueous ammonium acetate buffer at pH 5.5 and the final gadolinium complexes **7a**-**7d** were obtained after purification by reversed-phase chromatography.

### Analysis of Relaxivity

To evaluate the influence of the CSL in sulfobetaines on the relaxometric properties of the gadolinium complexes, a series of relaxivity measurements was performed. Initial experiments focused on the spacer-dependent relaxivity in aqueous solution at a magnetic field strength of 1.4 T (60 MHz, 37 °C). The longitudinal relaxivity *r*_1_ values were determined using a standard inversion–recovery pulse sequence. The reciprocal spin–lattice relaxation time 1/*T*_1_ was plotted as a function of complex concentration and the *r*_1_ relaxivity was determined *via* linear regression (Figure 1). Complex concentrations were measured precisely by inductively coupled plasma mass spectrometry (ICP-MS). The *r*_1_-values of all four gadolinium complexes were similar when measured in pure water. Only [Gd-(C_2_ZW-DOTA)] **7b**, had a slightly higher *r*_1_ value of 8.33 ± 0.14 mm ^–1^ s^–1^. The CSL of the zwitterionic sulfobetaines has thus only a minor impact on the relaxivity of the complexes in water at low magnetic field strength. The observed relaxivity values are high compared to the smaller [Gd-(DOTA)] (*r*_1_ = 3.6 mm ^–1^ s^–1^)^46^ and also to other [Gd-(DOTA)]-derivatives of comparable size as we noted in a previous study of [Gd-(C_3_ZW-DOTA)] **7c**.^23^ The observed high relaxivity compared to other [Gd-(DOTA)] complexes of similar structure and size^23^ are most likely due to the strong hydration of the sulfobetaine groups leading to an increase of the hydrodynamic diameter of the complex and thus also an increase of the rotational correlation time τ_r_, thereby enhancing relaxivity. Other factors are also likely contributing to increased relaxivity, because strongly hydrated groups assemble water molecules with a relatively long residence time in close vicinity to the metal center.^47,48^ However, this study is limited with respect to the detailed analysis of second-sphere hydration and its impact on relaxivity. A more detailed investigation involving fast-field-cycling NMR-relaxometry would be desirable in the future.

**Figure 1:**
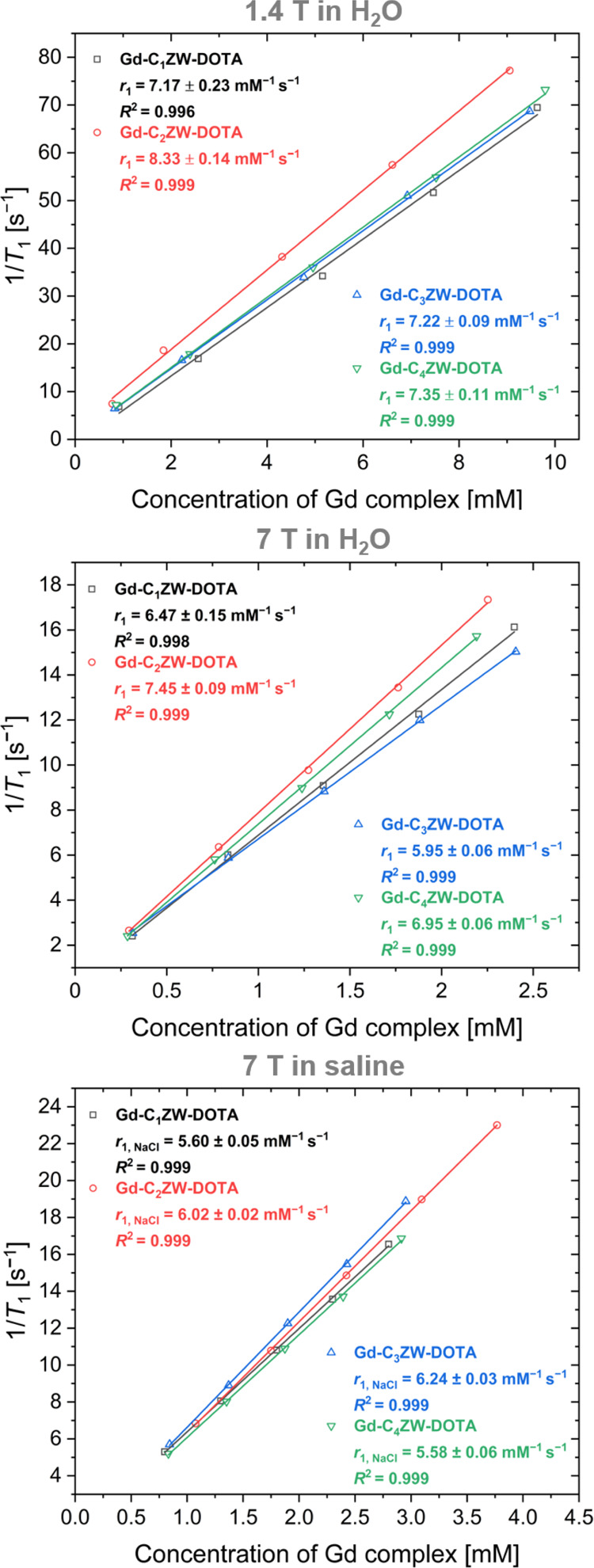
Longitudinal relaxivity (*r*_1_) determined from plotting the reciprocal relaxation time 1/*T*_1_ versus the concentration of the gadolinium complexes in H_2_O (37 °C, 1.4 T), in H_2_O (room temperature, 7 T) and in physiological saline (room temperature, 7 T).

To assess the field-strength dependence of relaxivity, additional measurements were performed at 7 T (300 MHz). The *r*_1_-values of all four gadolinium complexes were slightly reduced compared to 1.4 T and small differences in the extent of relaxivity reduction were observed. [Gd-(C_3_ZW-DOTA)] **7c** showed the largest decrease in *r*_1_ (−18%), followed by [Gd-(C_2_ZW-DOTA)] **7b** (−11%), [Gd-(C_1_ZW-DOTA)] **7a** (−10%) and [Gd-(C_4_ZW-DOTA)] **7d** (−6%), relative to the values at 1.4 T. [Gd-(C_2_ZW-DOTA)] **7b** was still the complex with the highest relaxivity of *r*_1_ = 7.45 ± 0.09 mm ^–1^ s^–1^. Overall, the data indicates a modest field strength dependence that varies with sulfobetaine spacer length. The combined observations at 1.4 and 7 T indicate that the sulfobetaine spacer length influences the relaxivity of the complexes only moderately. Furthermore, the observed effects are slightly larger at higher magnetic field strength.

Hydration and solubility of sulfobetaines are known to be sensitive to dissolved salts.^49^ Because both properties are important for GBCAs, the complexes were dissolved in physiological saline (0.154 m) and relaxivities were determined at 7 T (300 MHz). All complexes were completely soluble in concentrations of up to 0.5 m, indicating the absence of adverse salt effects. However, the *r*_1_-values were reduced significantly (−19.8% to – 13.5%, Figure 2) for the complexes [Gd-(C_1_ZW-DOTA)] **7a**, [Gd-(C_2_ZW-DOTA)] **7b** and [Gd-(C_4_ZW-DOTA)] **7d**. Only [Gd-(C_3_ZW-DOTA)] **7c** has an increased *r*_1_-value (+4.2%) in NaCl solution compared to water. The observed relaxivity reductions are likely due to partial destabilization of the hydration shell by ionic interactions with the zwitterions. These salt interactions of sulfobetaines have been investigated in detail for molecules bearing isolated zwitterionic groups and zwitterionic polymers.^35^ The fact that [Gd-(C_3_ZW-DOTA)] **7c** produced an overall increase in relaxivity contrary to the general trend of relaxivity loss, indicated that this “salt effect” does not follow a trivial dependence on spacer length.

**Figure 2:**
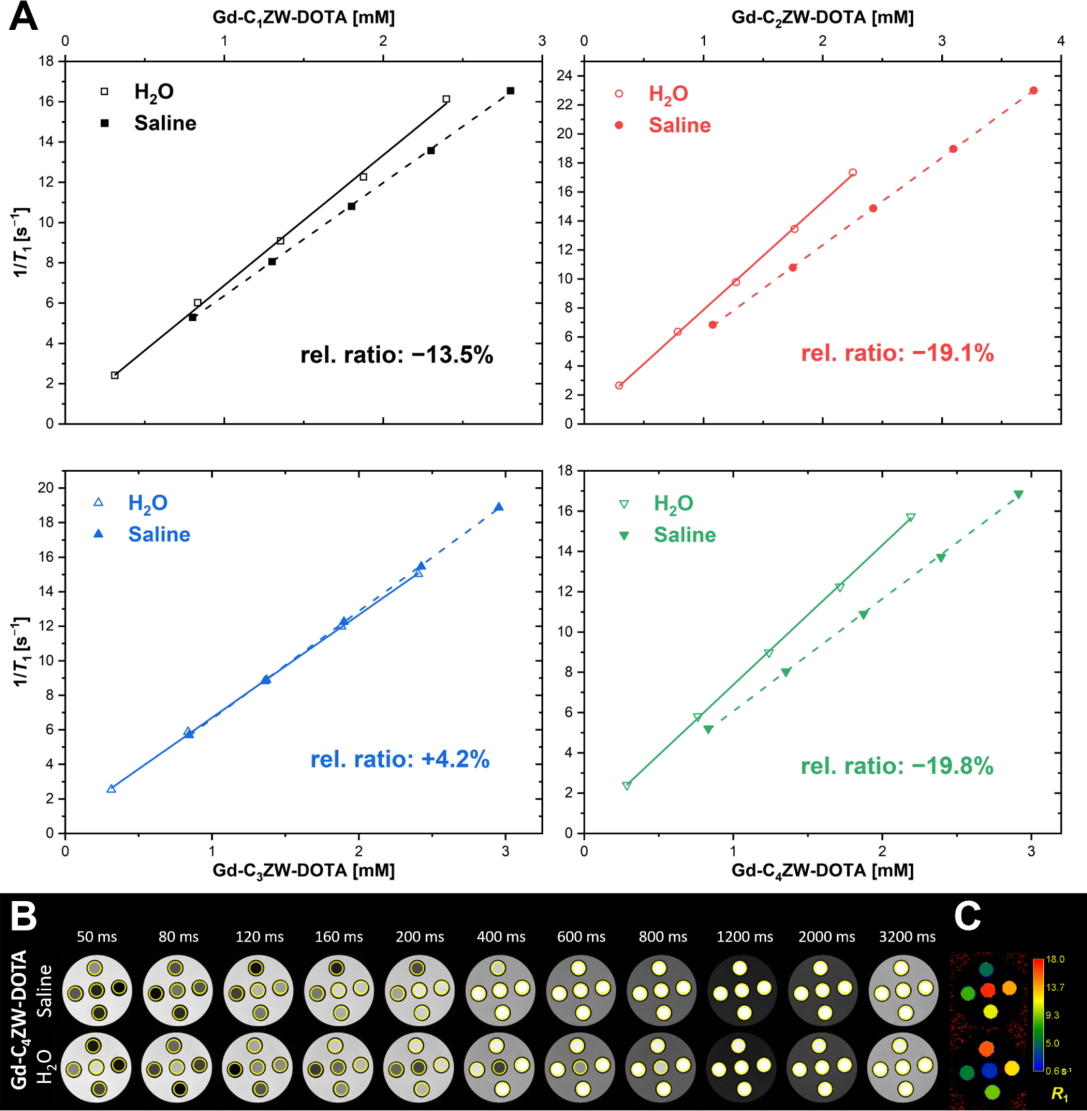
(A) Longitudinal relaxivity (*r*_1_) plots of [Gd-(C_1–4_ZW-DOTA)] complexes **7a**-**7d** measured in H_2_O and physiological saline at 7 T (300 MHz, room temperature). The relative *r*_1_ deviation in relaxivity between both media was calculated to assess the influence of ionic strength on the relaxometric performance. (B) Representative magnetic resonance images (MRI) acquired at 7 T at multiple inversion times (*TI*) ranging from 50 to 3200 ms of [Gd-(C_4_ZW-DOTA)] **7d** in H_2_O and physiological saline in a phantom holder surrounded by water. Yellow circles represent the regions of interest used for subsequent quantitative analysis. (C) Representative color-coded *R*_1_ relaxation rate map of [Gd-(C_4_ZW-DOTA)] **7d** in H_2_O and physiological saline acquired at 7 T with an inversion–recovery spin–echo sequence with multiple inversion time delays.

The dynamic contrast-enhancement (DCE) of the most promising GBCA **7c** was measured in BALB/c mice (n = 2). The GBCA (in saline) was administered intravenously in the tail vein at compound-specific doses. Prior to injection, the GBCA solutions were diluted to concentrations that matched the *in vitro* measured *T*_1_ value of Dotarem 1:12, which was used as reference. This adjustment was intended to yield a comparable blood-contrast-enhancement after weight-adjusted dosing.

*T*_1_-weighted MR images were acquired before and at multiple time points up to 24 h postinjection (Figure 3, for later time points see Figure S44). The DCE was subsequently quantified in the kidneys, brain and blood enabling a comparison of distribution and signal intensity. These preliminary *in vivo* experiments have several limitations, as both dose and relaxivity differ between the administered GBCAs. However, it reveals, that a similar contrast enhancement in mice can be achieved with a significantly reduced dose of the zwitterionic complex **7c** compared to [Gd-(DOTA)]. The time-signal curves reveal no differences in brain, and a slightly higher initial contrast in the kidney for com **7c** compared to [Gd-(DOTA)].

**Figure 3:**
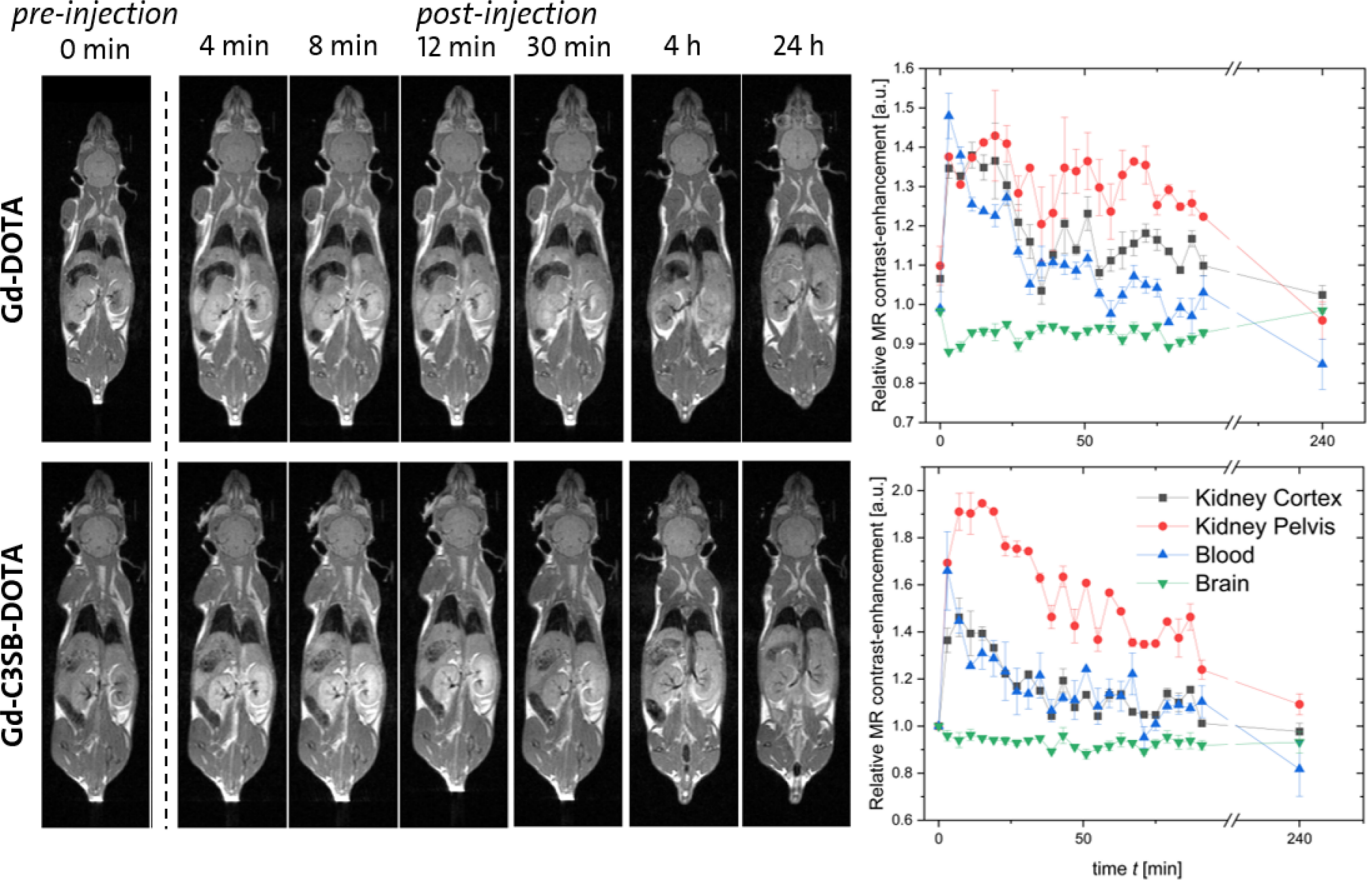
Dynamic contrast-enhanced MR images following GBCA administration. Sequential *T*_1_-weighted MR images acquired from 0 min (preinjection) up to 4 h postinjection after administration of [Gd-(DOTA)] (Dotarem, two male mice, dose: 280 μmol Gd/kg) and [Gd-(C_3_ZW-DOTA)] **7c** (two male mice, dose: 63 μmol Gd/kg). The images illustrate the temporal evolution of contrast-enhancement and subsequent clearance. Corresponding dynamic contrast-enhancement (DCE) time-signal curves are shown alongside the images and depict the signal-time profile for each GBCA.

## Conclusions

The decoration of GBCAs with sulfobetaines provides good water solubility, high complex stability and high relaxivity leading to favorable *in vivo* performance as demonstrated previously for selected examples.^23,27,36^ This study provides a systematic comparison of GBCAs **7a**-**d** bearing sulfobetaine groups with different CSL. It provides also an improved and safer synthetic protocol to zwitterionic GBCAs by CuAAC. The analysis of the compounds at 1.4 and 7 T in water and physiological saline revealed that the CSL has only a minor influence on relaxivity in deionized water. A significant reduction in relaxivity (14–20%) was observed for GBCAs **7a**,**b** (CSL = 1,2) and **7d** (CSL = 4) in saline. The highest relaxivity in saline was observed for GBCA **7c** (CSL = 3). The relaxivity of **7c** was almost unaffected by saline compared to deionized water. It is notable that all four complexes tested are close structural analogues of comparable molecular size and weight. As such, they share most likely the same hydration number, which has been determined before for similar tetrasubstituted DOTA-derivatives to be *q* ∼ 1.^50^ The complex geometry of **7c** and similar tetrasubstituted DOTA-derivatives has been determined to be TSAP in water.^23,27^ The zwitterionic GBCAs **7a**-**d** differ thus only in the CSL (one to four methylene groups). The observed high relaxivity compared to other [Gd-(DOTA)] complexes of similar structure and size^23^ are most likely due to the strong hydration of the sulfobetaine groups leading to an increase of the hydrodynamic diameter of the complex and thus also an increase of the rotational correlation time τ_r_, thereby enhancing relaxivity. Other factors are also likely contributing to increased relaxivity, because strongly hydrated groups assemble water molecules with a relatively long residence time in close vicinity to the metal center.^47,48^ However, this study is limited with respect to the detailed analysis of second-sphere hydration and its impact on relaxivity. A more detailed investigation involving fast-field-cycling NMR-relaxometry would be desirable in the future.

The hydration properties of sulfobetaines with different spacer length are dependent on the polarity of the zwitterions, which increases with increasing CSL, and other factors such as the formation of intramolecular tight ion pairs through charge interaction of the sulfonate with the ammonium group.^35,51^ Sulfobetaines with a CSL of three have the most negative hydration free energy according to DFT calculations.^52^ It is also known, that the hydration of sulfobetaines is compromised by ions in aqueous solution due to charge shielding. This ionic strength sensitivity of hydration is growing with increasing CSL because of the augmentation in partial charge by charged group separation.^35^ For the zwitterionic contrast agents investigated here, both factors (hydration and salt response) are important. A CSL of three methylene groups seems to be optimal with respect to charge separation. It leads to sulfobetaine groups with strong hydration and a low sensitivity to the ionic strength of the media. Both factors lead to strong hydration and thus high relaxivity of GBCA **7c** in saline *and* deionized water. GBCA **7c** was therefore selected for a first *in vivo* evaluation in mice. These preliminary experiments (n = 2) revealed a superior *in vivo* contrast enhancement of **7c** compared to [Gd-(DOTA)].

It is also notable that sulfobetaines with a CSL of three are easily accessible by nucleophilic ring opening of commercially available 1,3-propane sultone with tertiary amines and are therefore a particularly attractive choice for zwitterionization of GBCAs.

## Experimental Section

### General

[Gd-(C_3_ZW-DOTA)] **7c** was synthesized as described previously by Holzapfelet al.^23^ All commercially available reagents and starting materials were purchased from Sigma-Aldrich, TCI, abcr or BLDpharm and were used without further purification. Nondeuterated solvents in HPLC grade were purchased from VWR chemicals and deuterated solvents were purchased from Deutero GmbH. Water was purified using an ELGA PURELAB Classic UV water system. Reactions were monitored *via* HPLC-MS or TLC (Macherey Nagel TLC aluminum sheets, ALUGRAMSIL G UV254, 2.5 cm × 7.5 cm). Spots were visualized under UV light and/or by staining with a basic aqueous KMnO_4_ solution.

Medium pressure liquid chromatography was performed on automated systems using prepacked cartridges (Interchim). Normal phase chromatography was performed using a Biotage Isolera Prime system and for reversed flash chromatography on an Interchim PuriFlash 430 system with MeCN/H_2_O containing 0.1% formic acid as the mobile phase.

NMR analyses were performed on a Bruker Avance III HD 600 MHz, Bruker Avance I 400 MHz and Bruker Fourier 300 spectrometers. Chemical shifts (δ) are expressed in parts per millions (ppm).

Analytical HPLC-MS was performed on an Agilent HPLC system 1260 Infinity II with a Supelco SeQuant ZIC-pHILIC, 5 μm polymeric beads, 150 × 2.1 mm column linked to a Bruker amaZon SL ion trap mass spectrometer with ESI ionization.

### Relaxivity Measurements at 1.4 T (60 MHz)

The determination of the longitudinal relaxivity at 1.4 T (60 MHz) was conducted based on the spin–lattice relaxation time (*T*_1_) using a Bruker Minispec mq60 analyzer at 37 °C. *T*_1_ measurements were performed using the standard inversion recovery pulse sequence (180°-τ-90°) at a temperature of 37 °C ± 0.1 °C. The longitudinal relaxivity *r*_1_ was obtained *via* plotting the inverse relaxation time 1/*T*_1_ versus the concentration of the complexes and slope determination *via* the linear regression function in OriginLab 2022. The concentration of the complexes was determined *via* ICP-MS of the highest sample concentration.

### Relaxivity Measurements at 7 T (300 MHz)

Relaxation time measurements at 7 T (300 MHz) were conducted using a preclinical MRI scanner (Bruker Biospec 70/30, Ettlingen) at room temperature with a volume receive coil of an inner diameter of 40 mm. Five PCR tubes of 320 μL were positioned in a holder within a 50 mL Falcon tube filled with water, aligned parallel to the magnet bore. All imaging sequences utilized transversal orientation.

Following an initial survey scan, two distinct *T*_1_-measurement protocols were implemented. A 2D Look Locker EPI-based sequence provided quick *T*_1_ evaluation within 4 min. This protocol employed inversion times (*TI*) ranging from 20 to 770 ms in 50 ms increments, with TR 5000 ms and TE 9.9 ms. K-space acquisition utilized six segments with 8 averages, 10° RF pulses, and a 200 kHz readout bandwidth. Imaging parameters included a 32 mm field of view, 128 × 128 matrix, and 1.2 mm slice thickness.

Subsequently, a slice-selective 2D inversion recovery spin echo sequence was acquired over approximately 2 h. This protocol incorporated 11 inversion times (50, 80, 120, 160, 200, 400, 600, 800, 1200, 2000, and 3200 ms) with TE 7.0 ms, TR 5000 ms, and an 80 kHz readout bandwidth. Three slices of 1 mm thickness were obtained with 0.5 mm interslice gaps and 30 mm field of view with a 128 × 128 matrix.

Relaxation time analysis was conducted using qMapIt, an in-house quantification software that extends ImageJ functionality. Both Look-Locker and inversion recovery data sets underwent model function fitting:

$$f_{TI}=A\cdot \frac{1+e^{\left(-R_{1}\cdot TI\right)}-2\cdot e^{\left(-R_{1}\cdot TR\right)}}{1-2\cdot e^{\left(-R_{1}\cdot TR\right)}}$$ 1

with *T*_1_ = 1/*R*_1_ *via* a nonlinear least-squares Levenberg–Marquardt algorithm, generating amplitude A and relaxation time *T*_1_ maps. The inversion recovery data was smoothed before fitting with a Gaussian filter of 0.5 pixel to reduce Gibbs-ringing.

Automated contour detection identified individual tube boundaries, with segmented regions stored as regions of interest (ROIs) for subsequent quantitative analysis. The complete analytical workflow was orchestrated through a Python script executed within a Jupyter notebook environment, which seamlessly integrated Fiji and qMapIt operations *via* PyImageJ.

### Inductively Coupled Plasma Mass Spectrometry (ICP-MS)

Gadolinium concentrations were determined by inductively coupled plasma mass spectrometry (ICP-MS). Prior to ICP-MS measurements, 10 μL of the samples were digested in 1 mL of nitric acid (ROTIPURAN Supra, 69%) for 24 h at room temperature in precleaned tubes. Afterward, all samples were filled up to 12 mL with ultrapure water. The digested samples were then diluted 1:100 prior to analysis. The measurements were performed on an Agilent Technologies 7800x ICP-MS (Agilent Technologies Inc., Santa Clara, USA) equipped with a quadrupole mass analyzer. Prior to measurement, the ICP-MS setup was tuned with a mixture of Ce, Co, Li, Tl and Y at a concentration of 1 ppb (Agilent Technologies Inc., Santa Clara, USA). External calibration was performed using mixed element standards purchased from Merck KGaAA and PerkinElmer Inc. Calibration solutions containing Gd at concentrations of 0 to 1000 ppb were prepared freshly. Quantitation was performed by external calibration, corrected by internal standard (115 In for Gd). To ensure stability during the measurement a quality control sample containing 1 ppb Gd was measured.

### Synthesis

#### (*S*)-4-Azido-2-hydroxy-butanoic Acid **2**

NaN_3_ (3.33 g, 51.8 mmol, 1.67 equiv) was dissolved in a mixture of H_2_O (8.2 mL) and toluene (8.2 mL) and cooled to 0 °C. Afterward trifluoromethanesulfonic anhydride (5.2 mL, 31.0 mmol, 1.00 equiv) was added dropwise under vigorous stirring. Afterward the reaction mixture was stirred at 0 °C for 1 h and then at room temperature for 1 h. Sat. aq. NaHCO_3_ (8.2 mL) was added, the phases were separated, the aq. phase was extracted with toluene (2 × 8.2 mL) and the org. phases were combined. (*S*)-4-amino-2-hydroxybutyric acid (**1**) (4.21 g, 35.4 mmol, 1.14 equiv), CuSO_4_ pentahydrate (800 mg, 3.23 mmol, 0.10 equiv) and NaHCO_3_ (7.82 g, 93.0 mmol, 3.00 equiv) were dissolved in H_2_O (18 mL). Under vigorous stirring the priorly prepared TfN_3_ solution in toluene was added dropwise and a two phased reaction mixture was obtained. MeOH (100 mL) was added in a single portion which gave a homogeneous deep blue reaction mixture. The reaction mixture was stirred at room temperature for 48 h and afterward EtOAc (100 mL) was added. One m aq. HCl was added dropwise until the pH was found to be neutral and the phases separated again. The phases were separated, the aq. Phase was extracted with EtOAc (3 × 100 mL), the combined org. phases were washed with brine (100 mL) and dried over Na_2_SO_4_. The solvent was removed *in vacuo* (bath temperature: < 30 °C) and the crude product was obtained as a colorless oil. The crude product was purified *via* flash chromatography (*loaded as solution* in CH_2_Cl_2_/EtOAc/MeOH/AcOH 85:10:4:1 (10 mL), silica gel, MN cartridge BT40, CH_2_Cl_2_/EtOAc/MeOH/AcOH 85:10:4:1 *v*/*v*, isocratic) and (*S*)-4-azido-2-hydroxybutyric acid (**2**) (3.99 g, 88%) was obtained as a colorless liquid. ^1^H NMR (CD_3_OD, 300 MHz) δ = 4.21 (dd, ^3^ *J*_H,H_ = 4.09 Hz, 8.56 Hz, 1H, HO_2_C–CH), 3.47 (t, ^3^ *J*_H,H_ = 6.67 Hz, 2H, CH_2_–CH_2_–N_3_), 2.10–1.99 (m, 1H, CH_2_–CH_2_–N_3_), 1.91–1.80 (m, 1H, CH_2_–CH_2_–N_3_). ^13^C NMR (CD_3_OD, 75 MHz) δ = 177.3 (C_carbonyl_), 68.6 (HO_2_C–CH), 48.6 (CH_2_–CH_2_–N_3_), 34.4 (CH_2_–CH_2_–N_3_). HRMS (ESI) *m*/*z* [M–H]^−^ calcd. for C_4_H_6_N_3_O_3_^–^: 144.0415, found: 144.0422.

#### *tert*-Butyl-(*S*)-4-azido-2-hydroxybutanoate (**3**)

At 0 °C and under nitrogen atmosphere *O*-*tert*-butyl-*N*,*N*′-diisopropylisourea (4.97 g, 24.8 mmol, 3.00 equiv) was added dropwise to a solution of (*S*)-2-hydroxy-4-azidobutyric acid (**2**) (1.20 g, 8.27 mmol, 1.00 equiv) in dry CH_2_Cl_2_ (37 mL) over a period of 2 h. After warming to room temperature the colorless solution became a yellowish suspension and was then stirred at 35 °C for 46 h. After cooling to room temperature, the reaction mixture was filtered through a cellite plug, cellite was washed with CH_2_Cl_2_ (3 × 15 mL) and all volatiles were removed *in vacuo*. The crude product was obtained as a yellow liquid which contained residual colorless solids. The crude product was purified *via* flash chromatography (*loaded as solution* in pentane/EtOAc 9:1 (4 mL), silica gel, MN cartridge BT15, pentane/EtOAc 9:1 *v*/*v,* isocratic) and *tert*-butyl-(*S*)-4-azido-2-hydroxybutanoate (3) (967 mg, 58%) was obtained as a colorless oil. ^1^H NMR (CDCl_3_, 400 MHz) δ = 4.13 (dd, ^3^ *J*_H,H_ = 4.12 Hz, 7.52 Hz, ^*t*^BuO_2_C–CH), 3.46 (m, 2H, CH_2_–CH_2_–N_3_), 2.95 (s, 1H, OH), 2.08–2.00 (m, 1H, CH_2_–CH_2_–N_3_), 1.89–1.80 (m, 1H, CH_2_–CH_2_–N_3_), 1.50 (s, 9H, CH_3_). ^13^C NMR (CDCl_3_, 100 MHz) δ = 174.0 (C_carbonyl_), 83.2 (^*t*^BuO_2_C–CH), 68.0 (CH_2_–CH_2_–N_3_), 47.5 (CH_2_–CH_2_–N_3_), 33.4 (C­(CH_3_)_3_), 28.1 (CH_3_). HRMS (ESI) *m*/*z* [M + Na]^+^ calcd. for C_8_H_15_N_3_NaO_3_^+^: 224.1011, found: 224.1015.

#### (*S*)-Triflate **4**

Under nitrogen atmosphere a solution of *tert*-butyl-(*S*)-4-azido-2-hydroxybutanoate (**3**) (2.34 g, 11.6 mmol, 1.00 equiv) in dry CH_2_Cl_2_ (90 mL) was cooled to – 20 °C and following 2,6-lutidine (2.03 mL, 17.4 mmol, 1.50 equiv) followed by trifluoromethanesulfonic anhydride (2.74 mL, 16.3 mmol, 1.40 equiv) were added dropwise which gave an orange and then deep red reaction mixture. The solution was stirred at – 20 °C for 1 h and following at 0 °C for 1 h. After removing all volatiles *in vacuo* (bath temperature: < 35 °C) the crude product was obtained as a yellow-brown oil. The crude product was purified *via* flash chromatography (*loaded as solution* in CH_2_Cl_2_/MeOH 98:2 (6 mL), silica gel, MN Cartridge BT40, CH_2_Cl_2_/MeOH 98:2 *v*/*v*, isocratic). (*S*)-triflate **4** (3.79 g, 98%) was obtained as an orange oil. ^1^H NMR (CDCl_3_, 600 MHz) δ = 5.11 (dd, ^3^ *J*_H,H_ = 6.99 Hz, 5.17 Hz), 1H, ^*t*^BuO_2_C–CH), 3.57–3.53 (1H, m, CH_2_–CH_2_–N_3_), 3.50–3.46 (1H, m, CH_2_–CH_2_–N_3_), 2.26–2.17 (2H, m, CH_2_–CH_2_–N_3_), 1.52 (s, 9H, CH_3_). ^13^C NMR (CDCl_3_, 100 MHz) δ = 165.7 (C_carbonyl_), 118.6 (q,^1^ *J*_C–F_ = 320.5 Hz, CF_3_), 85.2 (^*t*^BuO_2_C–CH), 80.8 (C­(CH_3_)_3_), 46.3 (CH_2_–CH_2_–N_3_), 31.5 (CH_2_–CH_2_–N_3_), 27.9 (CH_3_). ^19^F-NMR (CDCl_3_, 564 MHz) δ = – 74.9 (OTf).

#### ^*t*^Bu_4_-DOTAZA **5**

To a solution of cyclen (410 mg, 2.38 mmol, 1.00 equiv) in dry MeCN (20 mL) was added Cs_2_CO_3_ (3.41 g, 10.4 mmol, 4.40 equiv) followed by the dropwise addition of a solution of (*S*)-triflate **4** (3.33 g, 9.99 mmol,4.20 equiv) in dry MeCN (20 mL). The reaction mixture was stirred at room temperature for 46 h and all volatiles were removed *in vacuo.* The residue was dissolved in a mixture of sat. aq. NaHCO_3_ solution (75 mL) and CH_2_Cl_2_ (50 mL). After stirring for 10 min the phases were separated and the aq. Phase was extracted with CH_2_Cl_2_ (5 × 40 mL). The combined org. phases were dried over Na_2_SO_4_ and the solvent was removed *in vacuo*. The crude product was obtained as an orange oil. The crude product was purified *via* flash chromatography (*loaded as solution* in CH_2_Cl_2_/MeOH/Et_3_N 95:4.5:0.5 *v*/*v* (5 mL), irregular silica gel, MN Cartridge BT40, CH_2_Cl_2_/MeOH/Et_3_N 95:4.5:0.5 *v*/*v*, isocratic). The product was obtained as a colorless solid. Due to the waxy consistency of the product solvents were hardly removed *via* rotavap. Thus, the product was dissolved in MeCN/H_2_O 80:20 and lyophilized. After lyophilization ^*t*^Bu_4_-DOTAZA **5** (1.92 g, 89%) was obtained as an orange lyophilizate. *R*_f_ (CH_2_Cl_2_/MeOH/Et_3_N 95:4.5:0.5 *v*/*v*) = 0.5. ^1^H NMR (CDCl_3_, 400 MHz) δ = 3.44 (t, ^3^ *J*_H,H_ = 7.30 Hz, 4H, ^*t*^BuO_2_C–CH), 3.27–3.21 (m, 4H, CH_2_–CH_2_–N_3_), 3.19–3.08 (m, 4H, CH_2_–CH_2_–N_3_), 3.05–2.73 (m, 16H, N–CH_2_–CH_2_–N), 1.98–1.87 (m, 4H, CH_2_–CH_2_–N_3_), 1.79–1.65 (m, 4H, CH_2_–CH_2_–N_3_), 1.41 (s, 36H, CH_3_). ^13^C NMR (CDCl_3_, 150 MHz) δ = 171.5 (C_carbonyl_), 80.9 (C­(CH_3_)_3_), 61.7 (^*t*^BuO_2_C–CH), 51.7 (N–CH_2_–CH_2_–N), 49.0 (CH_2_–CH_2_–N_3_), 29.4 (CH_2_–CH_2_–N_3_), 28.3 (CH_3_). HRMS (ESI) *m*/*z* [M + H]^+^ calcd. for C_40_H_73_N_16_O_8_^+^: 905.5792, found: 905.5813.

#### C_1_-Alkyne **10**

Sodium hydroxymethanesulfinate (26.3 g, 196 mmol, 1.00 equiv) was dissolved in H_2_O (100 mL) and cooled to 0 °C with an ice bath. Under vigorous stirring an aq. dimethylamine solution (40% *w*/*w*, 50 mL, 0.40 mol, 2.00 equiv) was added dropwise, the reaction mixture was stirred at 0 °C for 30 min and then at room temperature for 24 h. The reaction mixture was lyophilized and the isolated crude sodium dimethylaminomethanesulfonate **9** (29.2 g) was used in the next step without further purification. A solution of 3-bromoprop-1-yne (**8**) (1.15 mL, 10.3 mmol, 1.11 equiv) in EtOH (8 mL) was cooled to 0 °C with an ice bath and afterward a solution of sodium dimethylaminomethanesulfonate (**9**) (1.50 g, 9.31 mmol, 1.00 equiv) in EtOH/H_2_O 1:1 *v*/*v* (20 mL) was added dropwise. The reaction mixture was warmed to 50 °C and stirred at this temperature for 20 h. After lyophilization the ^1^H NMR spectrum showed incomplete conversion of the starting material. Thus, the crude mixture was dissolved in EtOH/H_2_O 1:1 *v*/*v* (20 mL) again, 3-bromoprop-1-yne (**8**) (0.30 mL, 2.7 mmol, 0.29 equiv) was added and the reaction mixture was stirred at 55 °C for 18 h. After lyophilization the C_1_-alkyne **10** (2.47 g) was obtained as a colorless lyophilizate. The crude product contained residual NaBr and was used without further purification in the next step. HRMS (ESI) *m*/*z* [M + H]^+^ calcd. for C_6_H_12_NO_3_S^+^: 178.0532, found: 178.0530.

#### C_2_-Alkyne **13**

3-Dimethylamino-1-propyne (**11**) (2.70 mL, 25.0 mmol, 4.00 equiv) was dissolved in dry DMF (25 mL) under nitrogen and the solution was warmed to 70 °C. Following 2-bromoethanesulfonate (**12**) (1.32 g, 6.25 mmol, 1.00 equiv) was added in a single portion and the reaction mixture was stirred at 70 °C for 18 h during which a colorless precipitate formed. The reaction mixture was cooled to 4 °C, the precipitate isolated *via* filtration, washed with cold dry DMF (40 mL), pentane (80 mL) and dried under high vacuum. C_2_-alkyne **13** (713 mg, 60%) was isolated as a colorless solid. ^1^H NMR (CDCl_3_, 500 MHz) δ = 4.34 (d, ^4^ *J*_H,H_ = 2.14 Hz, C≡C–CH_2_), 3.87–3.84 (m, 2H, CH_2_–CH_2_–SO_3_), 3.49–3.46 (m, 2H, CH_2_–CH_2_–SO_3_), 3.34 (t, ^4^ *J*_H,H_ = 2.46 Hz, H–C≡C), 3.26 (s, 6H, CH_3_). ^13^C NMR (CDCl_3_, 150 MHz) δ = 84.5 (H–C≡C), 72.6 (H–C≡C), 61.8 (CH_2_–CH_2_–SO_3_), 57.2 (C≡C–CH_2_), 53.3 (CH_3_), 46.7 (CH_2_–CH_2_–SO_3_). HRMS (ESI) *m*/*z* [M + H]^+^ calcd. for C_7_H_14_NO_3_S^+^: 192.0689, found: 192.0694.

#### C_3_-Alkyne **15**

To a solution of 3-dimethylamino-1-propyne (**11**) (3.60 mL, 36.0 mmol, 1.00 equiv) in toluene (150 mL) was added 1,3-propanesultone (**14**) (9.6 g, 6.9 mL, 2.20 equiv) in a single portion at room temperature and the solution was stirred at this temperature for 18 h. The colorless precipitate was isolated *via* filtration, washed with toluene (3 × 15 mL), acetone (3 × 15 mL) and dried under high vacuum. C_3_-alkyne **15** (5.41 g, 73%) was isolated as a colorless solid. ^1^H NMR (CDCl_3_, 600 MHz) δ = 4.32 (2H, d, ^4^ *J*_H,H_ = 2.57 Hz, C≡C–CH_2_), 3.64 (2H, m, CH_2_–SO_3_), 3.31 (1H, t, ^4^ *J*_H,H_ = 2.54 Hz, C≡C–H), 3.24 (6H, s, CH_3_), 3.02 (2H, t, ^3^ *J*_H,H_ = 7.24 Hz, N–CH_2_–CH_2_), 2.28 (2H, m, CH_2_–CH_2_–SO_3_). ^13^C NMR (CDCl_3_, 150 MHz) δ = 81.7 (C ≡C–H), 70.8 (C≡C–H), 62.4 (CH_2_–CH_2_–N­(CH_3_)_2_), 54.4 (CH_2_–C≡C), 50.4 (CH_3_), 47.2 (CH_3_), 18.3 (O_3_S-CH_2_–CH_2_). HRMS (ESI) *m*/*z* [M + H]^+^ calcd. for C_8_H_16_NO_3_S^+^: 206.0846, found: 206.0848. Procedure adapted from Niu et al.^43^

#### C_4_-Alkyne **17**

Under nitrogen atmosphere 3-dimethylamino-1-propyne **11** (3.20 mL, 30.0 mmol, 1.00 equiv) was dissolved in dry toluene (20 mL) and subsequently a solution of 1,4-butane sultone **16** (3.90 mL, 38.0 mmol, 1.25 equiv) in toluene (10 mL) was dropwise over a period of 5 min. The reaction mixture was warmed to 50 °C and stirred at this temperature for 5 days, during which a colorless precipitate formed. After cooling to 4 °C the precipitate was isolated *via* filtration, washed with toluene (20 mL), acetone (5 mL) and Et_2_O (20 mL). After drying under high vacuum C_4_-alkyne **17** (1.15 g, 18%) was obtained as a colorless solid. ^1^H NMR (D_2_O, 400 MHz) δ = 4.28 (d, ^4^ *J*_H,H_ = 2.27 Hz, C≡C–CH_2_), 3.54–3.50 (m, 2H, CH_2_–SO_3_), 3.30 (t, ^4^ *J*_H,H_ = 2.59 Hz, 1H, C≡C–H), 3.21 (s, 6H, CH_3_), 3.04–3.00 (m, 2H, N–CH_2_–CH_2_), 2.04–1.96 (m, 2H, CH_2_–CH_2_–SO_3_), 1.90–1.82 (m, 2H, N–CH_2_–CH_2_). ^13^C NMR (D_2_O, 75 MHz) δ = 81.5 (C≡C–H), 70.4 (C≡C–H), 63.5 (CH_2_–SO_3_), 54.2 (C≡C–CH_2_), 50.4 (CH_3_), 49.9 (N–CH_2_–CH_2_), 21.0 (N–CH_2_–CH_2_, CH_2_–CH_2_–SO_3_). HRMS (ESI) *m*/*z* [M + H]^+^ calcd. for C_9_H_18_NO_3_S^+^: 220.1002, found: 220.1018.

#### ^*t*^Bu_4_-C_1_ZW-DOTA **6a**

Under nitrogen atmosphere a mixture of ^*t*^BuOH/H_2_O/DMF 2:2:1 (*v*/*v*, 8 mL) was degassed by purging the solution with nitrogen for 15 min under vigorous stirring. Afterward CuI (13.0 mg, 68.0 μmol, 0.21 equiv), sodium ascorbate (39.0 mg, 0.20 mmol, 0.60 equiv) and tris­((1-benzyl-4-triazolyl)­methyl)­amine (44.0 mg, 83 μmol, 0.25 equiv) were added and the degassing process was allowed to proceed for further 10 min. ^*t*^Bu_4_-DOTAZA **5** (300 mg, 331 μmol, 1.00 equiv) was added as a solution in DMF (1 mL) and the reaction mixture was degassed for further 15 min before C_1_-alkyne **10** (300 mg, 1.69 mmol, 5.10 equiv) was added in a single portion and the reaction mixture was warmed to 55 °C. The reaction mixture was stirred at this temperature for 20 h and the reaction progress was monitored *via* HPLC-MS. Since the reaction showed no completion C_1_-alkyne **10** (114 mg, 643 μmol, 1.94 equiv) and sodium ascorbate (39.0 mg, 0.20 μmol, 0.60 equiv) were added and the reaction mixture was stirred at 55 °C for 24 h. After cooling to room temperature QuadraPure TU (70 mg) was added and the suspension was stirred for 1 h. The resin was removed *via* filtration and after removing all volatiles *in vacuo* (bath temperature 60 °C) the crude product was obtained as a brownish waxy solid. The crude product was purified *via* reversed flash chromatography (*loaded as solution* in H_2_O (5 mL), RP C18ec silica 40–63 μm, MN cartridge RS15, H_2_O + 0.1% formic acid/MeCN + 0.1% formic acid 98:2 → 2:98 *v*/*v*) and after lyophilization ^*t*^Bu_4_-C_1_ZW-DOTA **6a** (181 mg, 34%) was obtained as a colorless lyophilizate. *t*_R_ (SeQuant ZIC-pHILIC, method 1): 13.1 min. ^1^H NMR (CD_3_OD, 400 MHz) δ = 8.45 (s, 4H, C–H_triazole_), 4.98 (m, 8H, N–CH_2_–SO_3_), 4.63 (m, 11H, CH–CH_2_, triazole-CH_2_–N), 4.32 (s, 8H, CH–CH_2_–CH_2_), 3.36 (s, 24H, N­(CH_3_)_3_), 3.30–2.36 (m, 24H, N–C_2_H_4_–N, CH–CH_2_–CH_2_), 1.53–1.45 (m, 36H, C­(CH_3_)_3_). ^13^C NMR (CDCl_3_, 150 MHz) δ = 83.5 (HMBC, C­(CH_3_)_3_), 73.6 (CH–CH_2_–CH_2_), 60.4 (N–CH_2_–SO_3_), 52.7 (N­(CH_3_)_2_), 48.4 (triazole-CH_2_–N), 43.6, 35.8, 35.5 (HSQC, N–C_2_H_4_–N, CH–CH_2_–CH_2_), 28.7 (C­(CH_3_)_3_). *Due to the conformational dynamics of the cyclen ring in the presence of formic acid, the NMR spectra exhibit line broadening and not all quarternary carbon signals are observed.* HRMS (ESI) *m*/*z* [M+2H]^2+^ calcd. for C_64_H_118_N_20_O_20_S_4_^2+^: 807.3852, found: 807.3893.

#### ^*t*^Bu_4_-C_2_ZW-DOTA **6b**

Under nitrogen atmosphere a mixture of ^*t*^BuOH/H_2_O/DMF 2:2:1 (*v*/*v*, 8 mL) was degassed by purging the solution with nitrogen for 15 min under vigorous stirring. Afterward CuI (13.0 mg, 68.0 μmol, 0.21 equiv), sodium ascorbate (39.0 mg, 0.20 mmol, 0.60 equiv) and tris­((1-benzyl-4-triazolyl)­methyl)­amine (43.0 mg, 81.0 μmol, 0.25 equiv) were added and the degassing process was allowed to proceed for further 10 min. ^*t*^Bu_4_-DOTAZA **5** (312 mg, 345 μmol, 1.00 equiv) was added as a solution in DMF (1 mL) and the reaction mixture was degassed for further 15 min before C_2_-alkyne **13** (299 mg, 1.56 mmol, 4.50 equiv) was added in a single portion and the reaction mixture was warmed to 55 °C. The reaction mixture was stirred at this temperature for 16 h and the reaction progress was monitored *via* HPLC-MS. After cooling to room temperature QuadraPure TU (70 mg) was added and the suspension was stirred for 1 h. The resin was removed *via* filtration and after removing all volatiles *in vacuo* (bath temperature 60 °C) the crude product was obtained as a brownish waxy solid. The crude product was purified *via* reversed phase flash chromatography (*loaded as solution* in H_2_O (5 mL), RP C18ec silica 40–63 μm, MN cartridge RS15, H_2_O + 0.1% formic acid/MeCN + 0.1% formic acid 98:2 → 2:98 *v*/*v*) and after lyophilization ^*t*^Bu_4_-C_2_ZW-DOTA **6b** (273 mg, 47%) was obtained as a colorless lyophilizate. *t*_R_ (SeQuant ZIC-pHILIC, method 1): 16.0 min. ^1^H NMR (CD_3_OD, 600 MHz) δ = 8.47 (m, 4H, C–H_triazole_), 4.80–4.64 (m, 19H, CH–CH_2_, N–CH_2_–CH_2_–SO_3_, triazole-CH_2_–N), 3.67, 3.47 (s, 16H, N–CH_2_–CH_2_–SO_3_, CH–CH_2_–CH_2_), 3.21 (s, 24H, N­(CH_3_)_3_), 3.2–2.34 (m, 24H, N–C_2_H_4_–N, CH–CH_2_–CH_2_), 1.52–1.29 (m, 36H, C­(CH_3_)_3_). ^13^C NMR (CD_3_OD, 150 MHz) δ = 136.7 (C_carbonyl_), 130.2 (C–H_triazole_), 60.6, 60.5 (N–CH_2_–CH_2_–SO_3_, CH–CH_2_–CH_2_), 59.3 (N–CH_2_–CH_2_–SO_3_, triazole-CH_2_–N), 51.7 (N­(CH_3_)_3_), 49.6 (CH), 45.8 (CH–CH_2_–CH_2_), 28.7 (C­(CH_3_)_3_). *Due to the conformational dynamics of the cyclen ring in the presence of formic acid, the NMR spectra exhibit line broadening and not all quarternary carbon signals are observed.* HRMS (ESI) *m*/*z* [M + H]^+^ calcd. for C_68_H_120_N_20_O_20_S_4_^+^ 835.4165, found: 835.4180.

#### ^*t*^Bu_4_-C_3_ZW-DOTA **6c**

Under nitrogen atmosphere a mixture of ^*t*^BuOH/H_2_O/DMF 2:2:1 (*v*/*v*, 50 mL) was degassed by purging the solution with nitrogen for 15 min under vigorous stirring. Afterward CuI (80.0 mg, 420 μmol, 0.20 equiv), sodium ascorbate (250 mg, 1.26 mmol, 0.60 equiv) and tris­((1-benzyl-4-triazolyl)­methyl)­amine (279 mg, 525 μmol, 0.25 equiv) were added and the degassing process was allowed to proceed for further 10 min. ^*t*^Bu_4_-DOTAZA **5** (1.90 g, 2.10 mmol, 1.00 equiv) was added as a solution in DMF (6 mL) and the reaction mixture was degassed for further 15 min before C_3_-alkyne **15** (1.94 g, 9.45 mmol, 4.50 equiv) was added in a single portion and the reaction mixture was warmed to 50 °C. The reaction mixture was stirred at this temperature for 16 h and the reaction progress was monitored *via* HPLC-MS. All volatiles *in vacuo* (bath temperature 60 °C) the crude product was obtained as a orange oil. The crude product was purified *via* reversed phase flash chromatography (*loaded as solution* in H_2_O (7 mL), RP POLYGOPREP C18 50–100 μm, MN cartridge BT40, H_2_O + 0.1% formic acid/MeCN + 0.1% formic acid 98:2 → 2:98 *v*/*v*) and after lyophilization ^*t*^Bu_4_-C_3_ZW-DOTA **6c** (2.31 g, 64%) was obtained as a colorless lyophilizate. ^1^H NMR (CD_3_OD, 600 MHz) δ = 8.58 (s, 4H, C–H_triazole_), 4.84–4.69 (m, 16H, CH–CH_2_–CH_2_, N–CH_2_–triazole), 3.56–3.45 (m, 12H, CH–CH_2_, N­(CH_3_)_2_-CH_2_–CH_2_), 3.16, 3.14 (s, 24H, N­(CH_3_)_2_), 2.94–2.86 (m, 8H, CH_2_–SO_3_), 2.65–2.61 (m, 4H, N–C_2_H_4_–N), 2.38–2.33 (m, 8H, CH_2_–CH_2_–SO_3_), 2.13–2.11 (m, 8H, CH–CH_2_), 1.87–1.75 (m, 8H, N–C_2_H_4_–N), 1.65 (m, 36H, C­(CH_3_)_3_), 1.54–1.46 (m, 4H, N–C_2_H_4_–N). ^13^C NMR (CD_3_OD, 150 MHz) δ = 176.0 (C_carbonyl_), 137.1 (quart. C_triazole_), 130.0 (C–H_triazole_), 84.3 (C­(CH_3_)_3_), 63.6 (N­(CH_3_)_2_-CH_2_–CH_2_), 59.5 (CH–CH_2_), 58.8 (N–CH_2_–triazole), 51.5, 51.3 (N­(CH_3_)_2_), 50.9 (CH–CH_2_–CH_2_), 47.9 (CH_2_–SO_3_), 45.4 (N–C_2_H_4_–N), 28.5 (C­(CH_3_)_3_), 26.8 (CH–CH_2_), 20.1 (CH_2_–CH_2_–SO_3_). HRMS (ESI) *m*/*z* [M+H+Na]^2+^ calcd. for C_72_H_133_N_20_O_20_S_4_Na^2+^ 874.4388, found: 874.4425.

#### ^*t*^Bu_4_-C_4_ZW-DOTA **6d**

Under nitrogen atmosphere a mixture of ^*t*^BuOH/H_2_O/DMF 2:2:1 (*v*/*v*, 8 mL) was degassed by purging the solution with nitrogen for 15 min under vigorous stirring. Afterward CuI (17.0 mg, 89.0 μmol, 0.27 equiv), sodium ascorbate (39.0 mg, 0.20 mmol, 0.60 equiv) and tris­((1-benzyl-4-triazolyl)­methyl)­amine (44.0 mg, 83.0 μmol, 0.25 equiv) were added and the degassing process was allowed to proceed for further 10 min. ^*t*^Bu_4_-DOTAZA **5** (300 mg, 331 μmol, 1.00 equiv) was added as a solution in DMF (1 mL) and the reaction mixture was degassed for further 15 min before C_4_-alkyne **17** (327 mg, 1.49 mmol, 4.50 equiv) was added in a single portion and the reaction mixture was warmed to 55 °C. The reaction mixture was stirred at this temperature for 17 h and the reaction progress was monitored *via* HPLC-MS. After cooling to room temperature QuadraPure TU (90 mg) was added and the suspension was stirred for 1 h. The resin was removed *via* filtration and after removing all volatiles *in vacuo* (bath temperature 60 °C) the crude product was obtained as a brownish waxy solid. The crude product was purified *via* reversed phase flash chromatography (*loaded as solution* in H_2_O (5 mL), RP C18ec silica 40–63 μm, MN cartridge RS15ec, H_2_O + 0.1% formic acid/MeCN + 0.1% formic acid 98:2 → 2:98 *v*/*v*) and after lyophilization ^*t*^Bu_4_-C_4_ZW-DOTA **6d** (298 mg, 50%) was obtained as a colorless lyophilizate. *t*_R_ (SeQuant ZIC-pHILIC, method 1): 23.0 min. ^1^H NMR (CD_3_OD, 600 MHz) δ = 8.62–8.42 (m, 4H, C–H_triazole_), 4.74–4.63 (m, 16H, CH–CH_2_–CH_2_, N–CH_2_–triazole), 3.38–3.35 (m, 12H, m, 12H, CH–CH_2_, N­(CH_3_)_2_-CH_2_–CH_2_), 3.16, 3.13 (s, 24H, N­(CH_3_)_2_), 3.08–2.10 (m, 32H, CH_2_–SO_3_, N–C_2_H_4_–N, CH_2_–CH_2_–SO_3_), 2.10 (s, 8H, CH_2_–CH_2_–CH_2_–SO_3_), 1.84 (s, 8H, CH_2_–CH_2_–CH_2_–SO_3_), 1.52–1.45 (m, 36H, C­(CH_3_)_3_). ^13^C NMR (CD_3_OD, 150 MHz) δ = 174.4 (C_carbonyl_), 136.9 (quart. C_triazole_) 129.9 (C–H_triazole_), 82.9 (C­(CH_3_)_3_), 69.3 (CH–CH_2_), 64.7 (N­(CH_3_)_2_-CH_2_–CH_2_), 64.5 (N­(CH_3_)_2_-CH_2_–CH_2_), 59.0 (CH_2_–SO_3_), 51.6 (CH_2_–CH_2_–SO_3_), 51.5 (CH_2_–CH_2_–SO_3_), 51.3 (N­(CH_3_)_2_), 51.1 (N­(CH_3_)_2_), 35.3 (CH–CH_2_–CH_2_), 28.7 (C­(CH_3_)_3_), 28.3 (C­(CH_3_)_3_), 23.2 (CH_2_–CH_2_–SO_3_), 22.5 (CH_2_–CH_2_–SO_3_). HRMS (ESI) *m*/*z* [M+H+Na]^2+^ calcd. for C_76_H_141_N_20_NaO_20_S_4_^+^: 902.4701, found: 902.4700.

#### [Gd-(C_1_ZW-DOTA)] **7a**

To a solution of ^*t*^Bu_4_-C_1_ZW-DOTA **6a** (41.0 mg, 25.4 μmol, 1.00 equiv) in TFA (7 mL) were added triisopropylsilane (32 mg, 0.20 mmol, 8.00 equiv) and H_2_O (50 μL). The reaction solution was stirred at 55 °C for 20 h and afterward all volatiles were removed *in vacuo*. The residue was dissolved in H_2_O (20 mL) and lyophilized. The isolated colorless lyphilizate was dissolved in 1 m aq. NH_4_Oac buffer (pH 5.5, 5 mL) and Gd_2_O_3_ (6.0 mg, 15 μmol, 0.6 equiv) was added. The suspension was warmed to 90 °C for 21 h during which a solution formed. The reaction solution was lyophilized and the crude product obtained as a colorless lyophilizate. The crude product was purified *via* reversed phase flash chromatography (*loaded as solution* in H_2_O (2 mL), RP C18ec silica 40–63 μm, MN cartridge RS15ec, H_2_O + 0.1% formic acid/MeCN + 0.1% formic acid 98:2 → 50:50 *v*/*v*) and after lyophilization [Gd-(C_1_ZW-DOTA)] **7a** (19 mg, 48%) was obtained as a colorless lyophilizate. *t*_R_ (SeQuant ZIC-pHILIC, method 1): 21.4 min. HRMS (ESI) *m*/*z* [M–H]^−^ calcd. for C_48_H_80_GdN_20_O_20_S_4_^–^: 1542.3987, found: 1542.4027.

#### [Gd-(C_2_ZW-DOTA)] **7b**

To a solution of ^*t*^Bu_4_-C_2_ZW-DOTA **6b** (150.0 mg, 90.0 μmol, 1.00 equiv) in TFA (8 mL) were added triisopropylsilane (114 mg, 0.72 mmol, 8.00 equiv) and H_2_O (150 μL). The reaction solution was stirred at 55 °C for 20 h and afterward all volatiles were removed *in vacuo*. The residue was dissolved in H_2_O (20 mL) and lyophilized. The isolated colorless lyophilizate was dissolved in 1 m aq. NH_4_OAc buffer (pH 5.5, 5 mL) and Gd_2_O_3_ (20.0 mg, 54 μmol, 0.6 equiv) was added. The suspension was warmed to 90 °C for 18 h during which a solution formed. The reaction solution was lyophilized and the crude product obtained as a colorless lyophilizate. The crude product was purified *via* reversed phase flash chromatography (*loaded as solution* in H_2_O (2 mL), RP C18ec silica 40–63 μm, MN cartridge RS15ec, H_2_O + 0.1% formic acid/MeCN + 0.1% formic acid 98:2 → 50:50 *v*/*v*) and after lyophilization [Gd-(C_2_ZW-DOTA)] **7b** (65 mg, 46%) was obtained as a colorless lyophilizate. *t*_R_ (SeQuant ZIC-pHILIC, method 1): 23.4 min. HRMS (ESI) *m*/*z* [M + H]^−^ calcd. for C_52_H_88_GdN_20_O_20_S_4_^–^ 1598.4613, found: 1598.4620.

#### [Gd-(C_4_ZW-DOTA)] **7d**

To a solution of ^*t*^Bu_4_-C_4_ZW-DOTA **6d** (121.0 mg, 67.9 μmol, 1.00 equiv) in TFA (10 mL) were added triisopropylsilane (86 mg, 0.54 mmol, 8.00 equiv) and H_2_O (110 μL). The reaction solution was stirred at 55 °C for 20 h and afterward all volatiles were removed *in vacuo*. The residue was dissolved in H_2_O (20 mL) and lyophilized. The isolated colorless lyophilizate was dissolved in 1 m aq. NH_4_OAc buffer (pH 5.5, 5 mL) and Gd_2_O_3_ (14.0 mg, 374 μmol, 0.55 equiv) was added. The suspension was warmed to 90 °C for 24 h during which a solution formed. The reaction solution was lyophilized and the crude product obtained as a colorless lyophilizate. The crude product was purified *via* reversed phase flash chromatography (*loaded as solution* in H_2_O (2 mL), RP C18ec silica 40–63 μm, MN cartridge RS15ec, H_2_O + 0.1% formic acid/MeCN + 0.1% formic acid 98:2 → 50:50 *v*/*v*) and after lyophilization [Gd-(C_4_ZW-DOTA)] **7d** (75 mg, 65%) was obtained as a colorless lyophilizate. *t*_R_ (SeQuant ZIC-pHILIC, method 1): 19.1 min. HRMS (ESI) *m*/*z* [M+2H]^2+^ calcd. for C_60_H_107_GdN_20_O_20_S_4_^2+^ 856.8042, found: 856.8066.

## Supplementary Material



## Notes

Characterization of new compounds, including NMR spectra and HPLC/MS analyses (PDF)

## Abbreviations

GBCAs: gadolinium-based contrast agents
DOTA: 1,4,7,10-tetraazacyclododecane-*N*,*N*′,*N*″,*N*‴-tetraacetic acid
CSL: carbon spacer length
CE-MRI: contrast-enhanced magnetic resonance imaging
CuAAC: copper-catalyzed azide–alkyne cycloaddition
TfN_3_: trifluoromethanesulfonyl azide
Tf_2_O: trifluoromethanesulfonic anhydride
DIU-O^*t*^Bu): *O*-*tert*-butyl-*N*,*N*′-diisopropylisourea
TFA: trifluoroacetic acid
TIPS: triisopropylsilane
ICP-MS: inductively coupled plasma mass spectrometry
