# Supplementary material for: Zwitterionic [Gd-(DOTA)] MRI Probes: Influence of Sulfobetaine Linker Length on Relaxivity
Source: ACS Omega. 2026 Apr 16;11(16):24623–34. doi: 10.1021/acsomega.6c00875 (PMC13129822; doi:10.1021/acsomega.6c00875)
Supplement: Supplementary file 1 [file ao6c00875_si_001.pdf]

SUPPORTING INFORMATION

Zwitterionic [Gd-(DOTA)] MRI Probes: Influence of Sulfobetaine Linker Length on Relaxivity

Lennart F. V. Spickschen,[a] David S. Pusztai,[a] Michael G. Kaul,[b] Verena R. Schulze,[c] Marie Oest,[d] Aleksander J. Swierzewski,[a] Neus Feliu,[c] Markus Fischer,[d] Theophraste Lescot,[e] Marc-André Fortin,[e] John V. Frangioni,[f] Wolfgang Maison[a]*

[a] Lennart F. V. Spickschen, David S. Pusztai, Aleksander J. Swierzewski, Prof. Dr. Wolfgang Maison, Department of Chemistry, Institute of Pharmacy, University of Hamburg Bundesstrasse 45, 20146 Hamburg, Germany, E-mail: wolfgang.maison@uni-hamburg.de; [b] Dr. Michael G. Kaul, Department of Diagnostic and Interventional Radiology and Nuclear Medicine, Center of Radiology and Endoscopy, University Medical Center Hamburg-Eppendorf, 20246 Hamburg, Germany; [c] Verena R. Schulze, Prof. Dr. Neus Feliu, Fraunhofer Institute for Applied Polymer Research IAP, Center for Applied Nanotechnology CAN, Grindelallee 117, 20146 Hamburg, Germany; [d] Marie Oest, Prof. Dr. Markus Fischer, Hamburg School of Food Science, Institute of Food Chemistry, University of Hamburg, Grindelallee 117, 20146 Hamburg, Germany; [e] Axe Oncologie, Centre de Recherche du CHU de Québec – Université Laval, 2705, boul. Laurier, Québec, QC, G1V4G2, Canada; Centre de Recherche sur le Cancer (CRC) de l’Université Laval, 9 Rue McMahon, Québec,

QC, G1R 3S3, Canada; Département de Génie des Mines, de la Métallurgie et des Matériaux, Université Laval, Québec, QC, G1V 0A6, Canada; [f] Dr. John V. Frangioni, Curadel Pharma, 28120 Hunters Ridge Blvd, Suites 6-7, Bonita Springs, FL MA 34135 (USA)

Characterization of new compounds, NMR spectra and HPLC-MS analyses

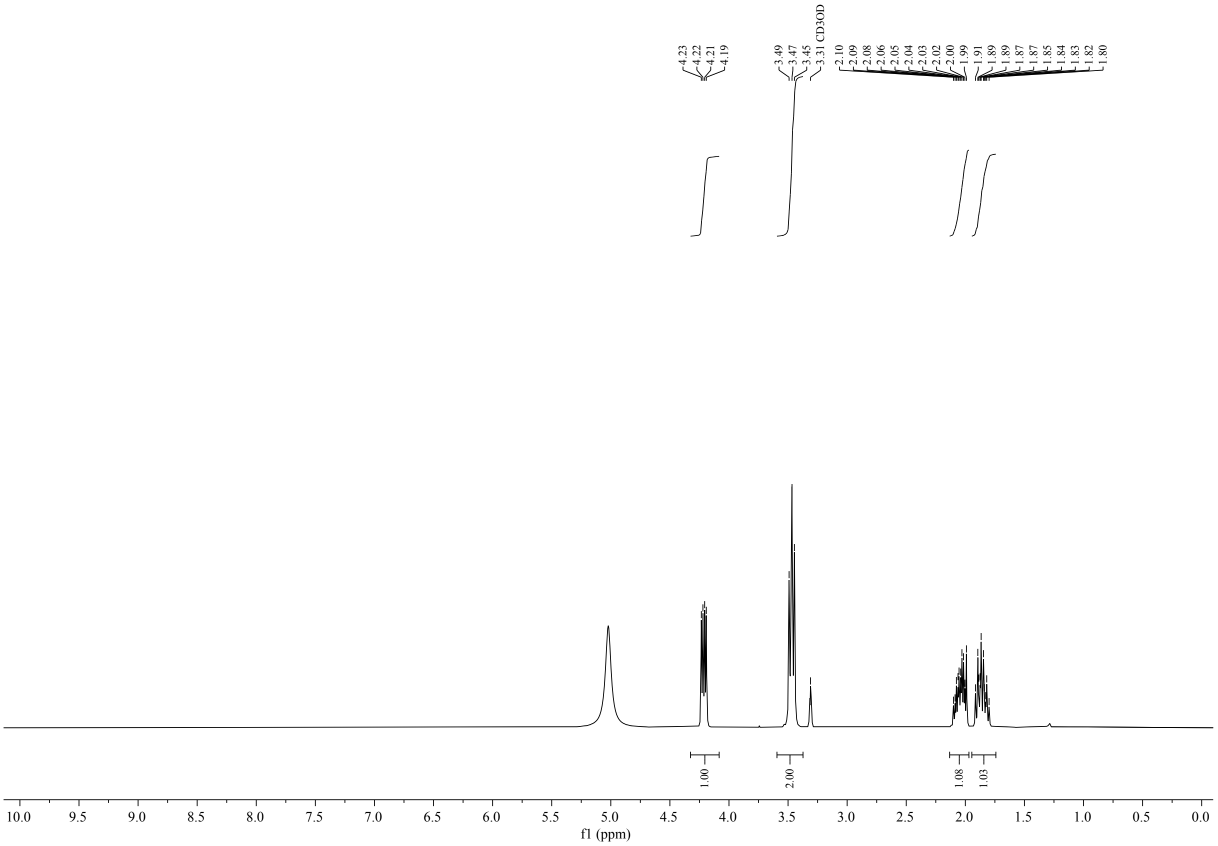

Figure S1: ^{1}H-NMR spectrum (300 MHz, CD_3OD) of (*S*)-azide **2**.

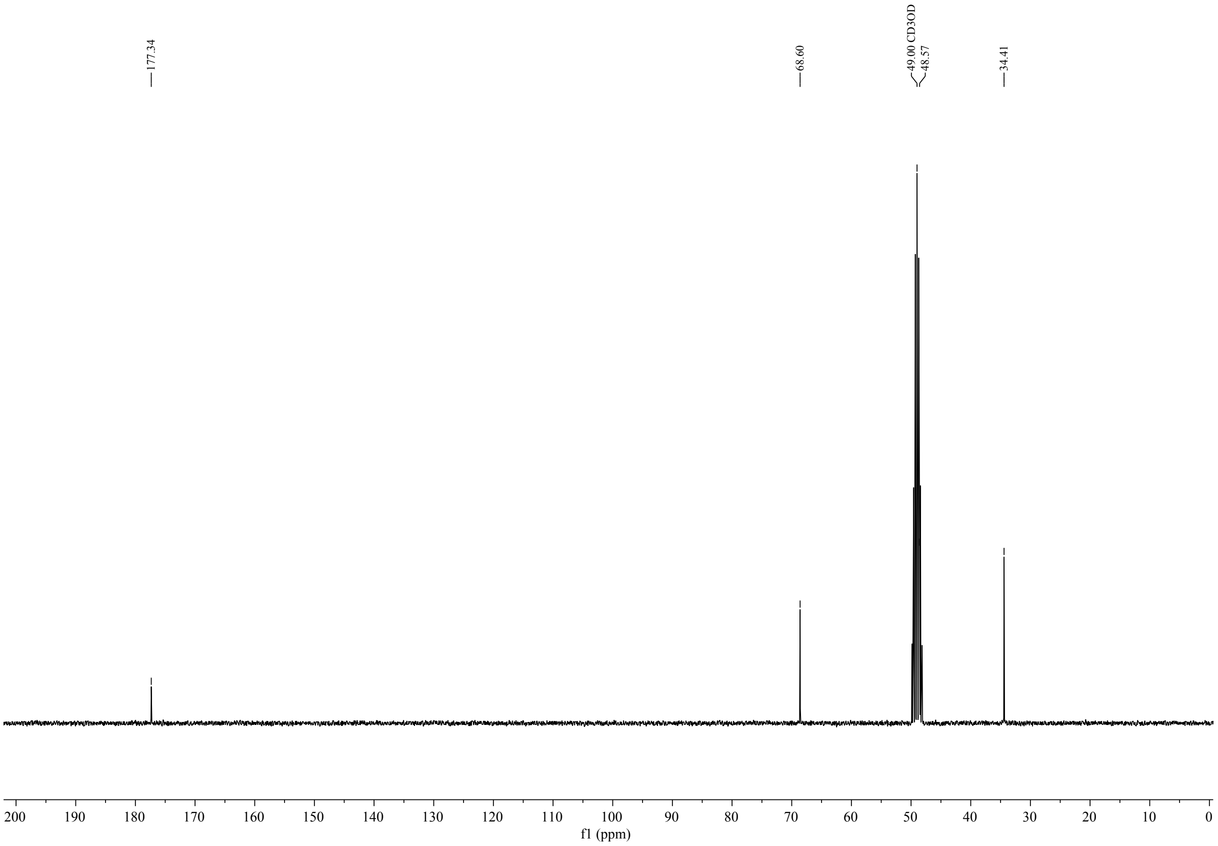

Figure S2: ^{13}C-NMR spectrum (75 MHz, CD_3OD) of (*S*)-azide **2**.

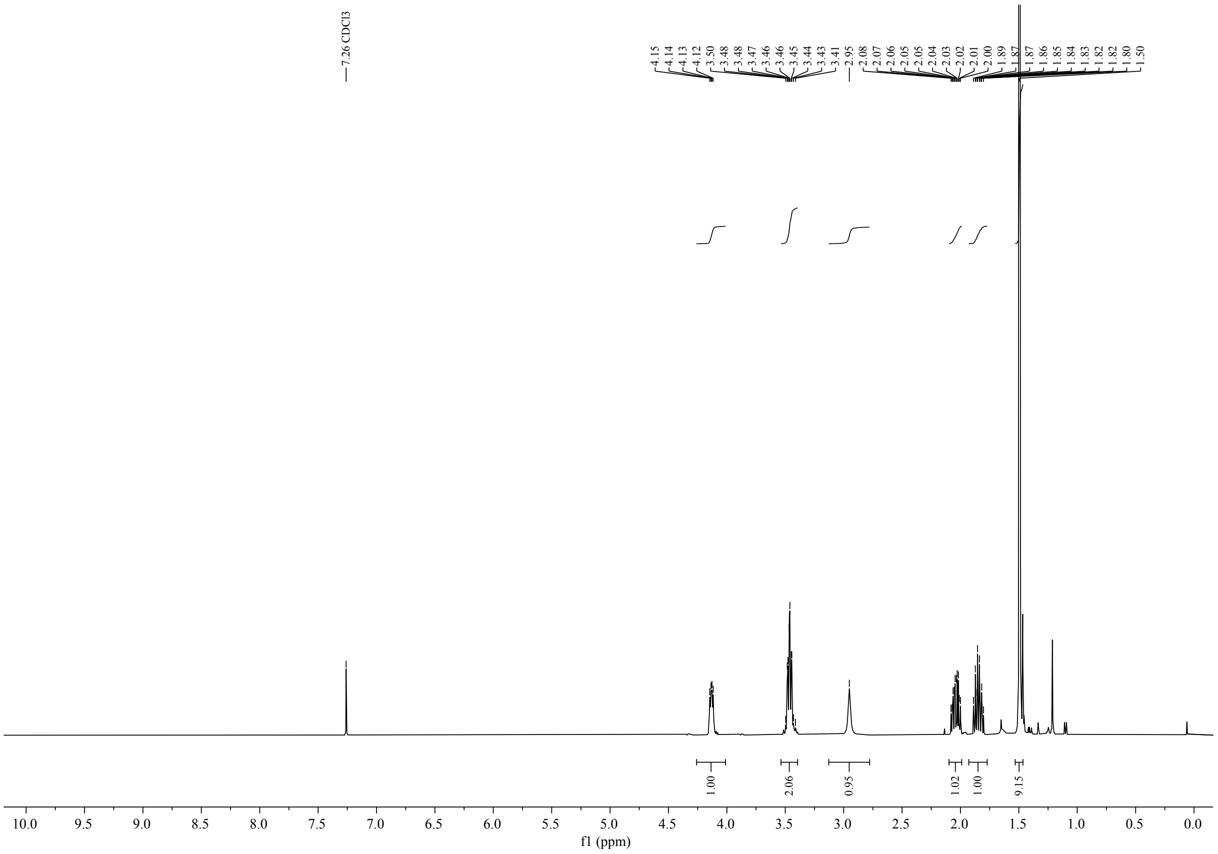

Figure S3: ^{1}H-NMR spectrum (400 MHz, $CDCl_3$) of (*S*)-ester **3**.

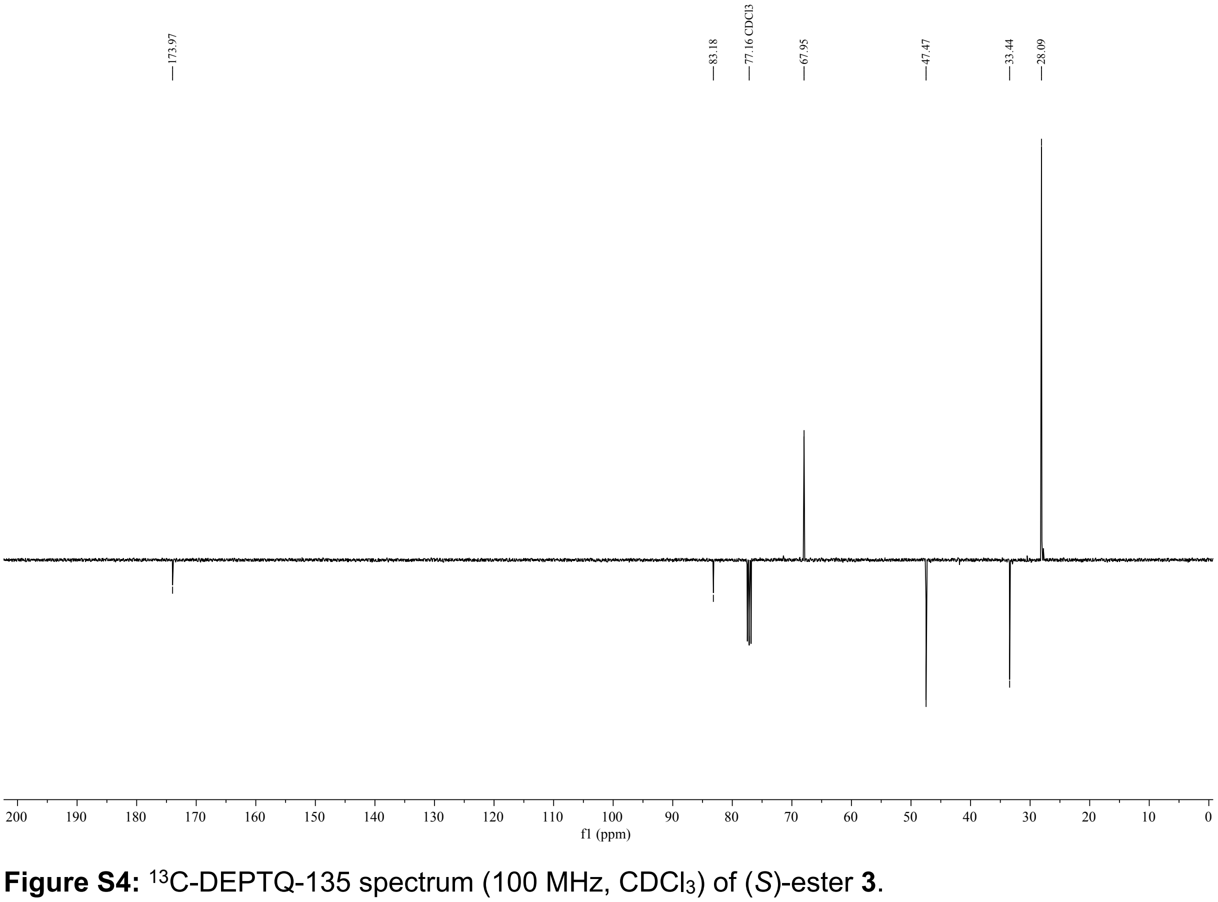

Figure S4: ^{13}C-DEPTQ-135 spectrum (100 MHz, $CDCl_3$) of (*S*)-ester **3**.

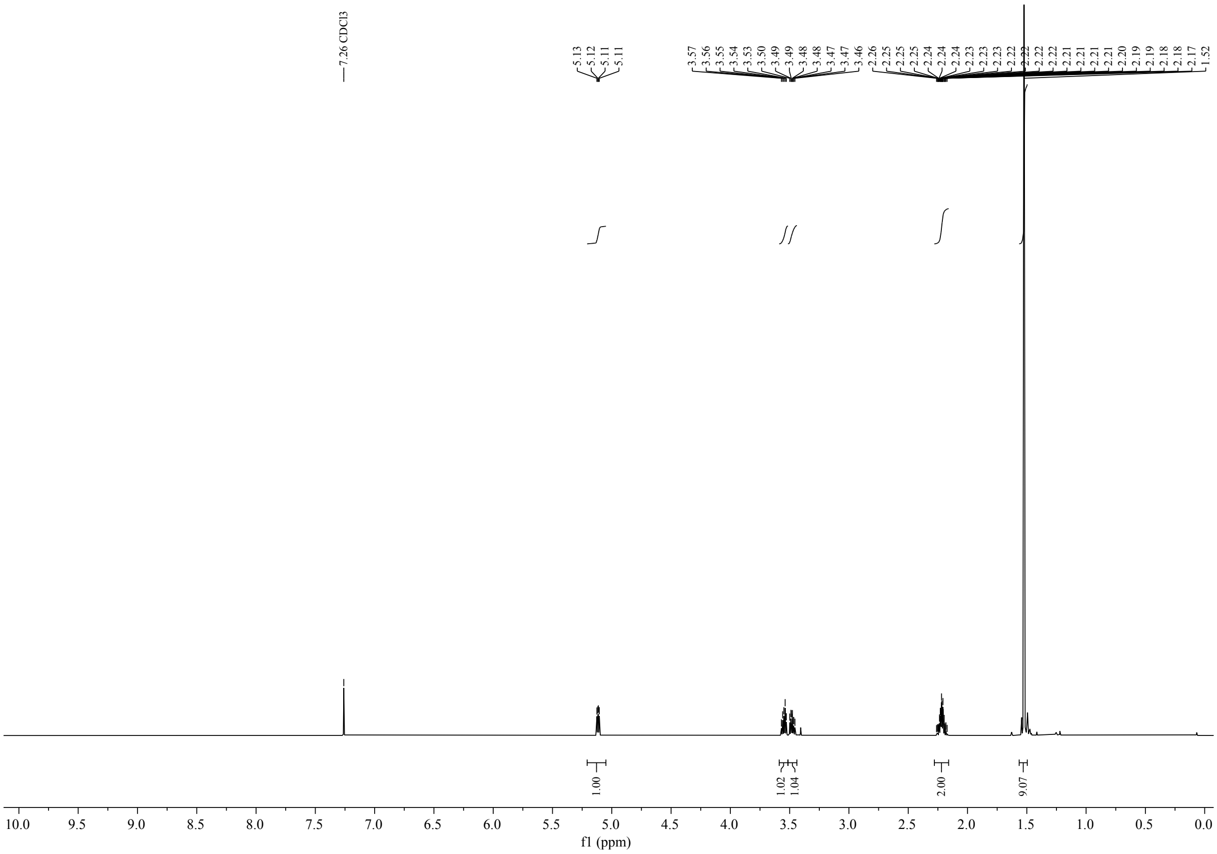

Figure S5: ^{1}H-NMR spectrum (600 MHz, $CDCl_3$) of (*S*)-triflate **4**.

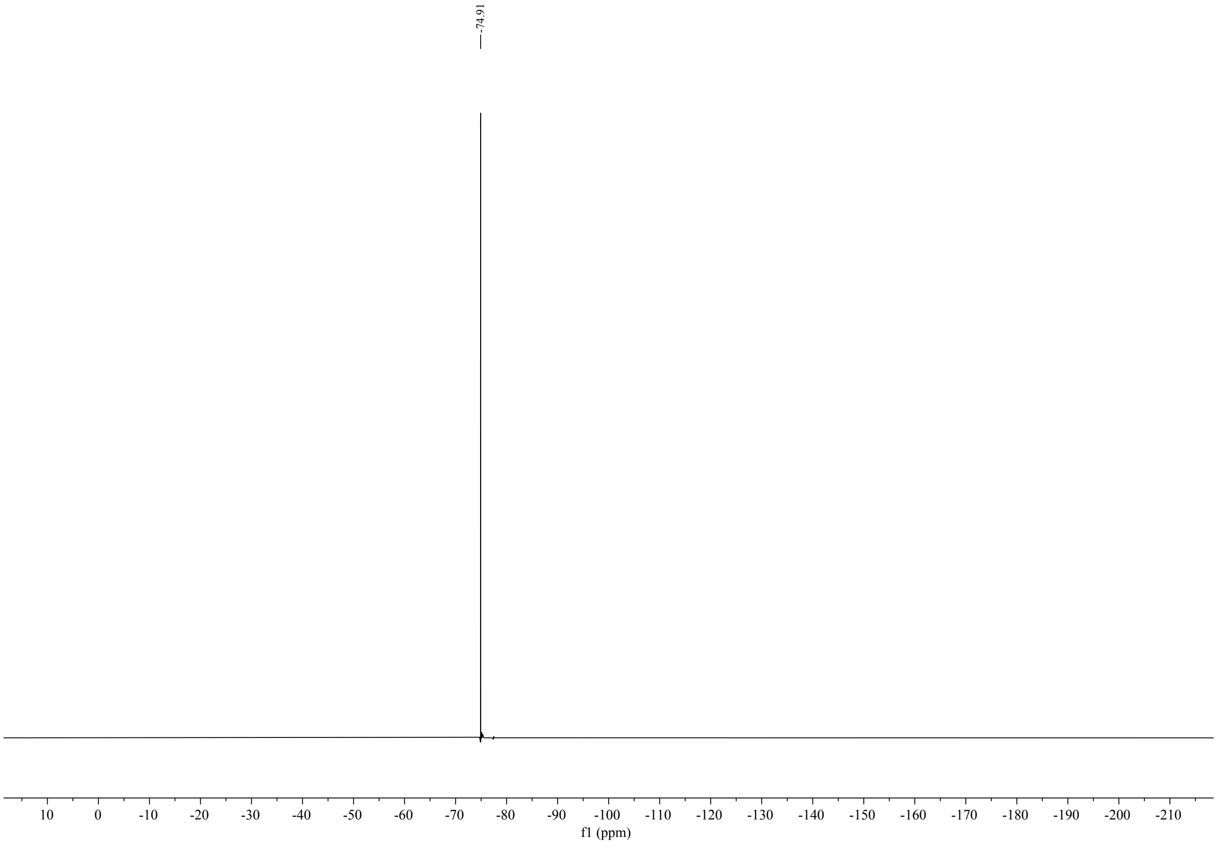

Figure S6: ^{19}F-NMR spectrum (564 MHz, $CDCl_3$) of (*S*)-triflate **4**.

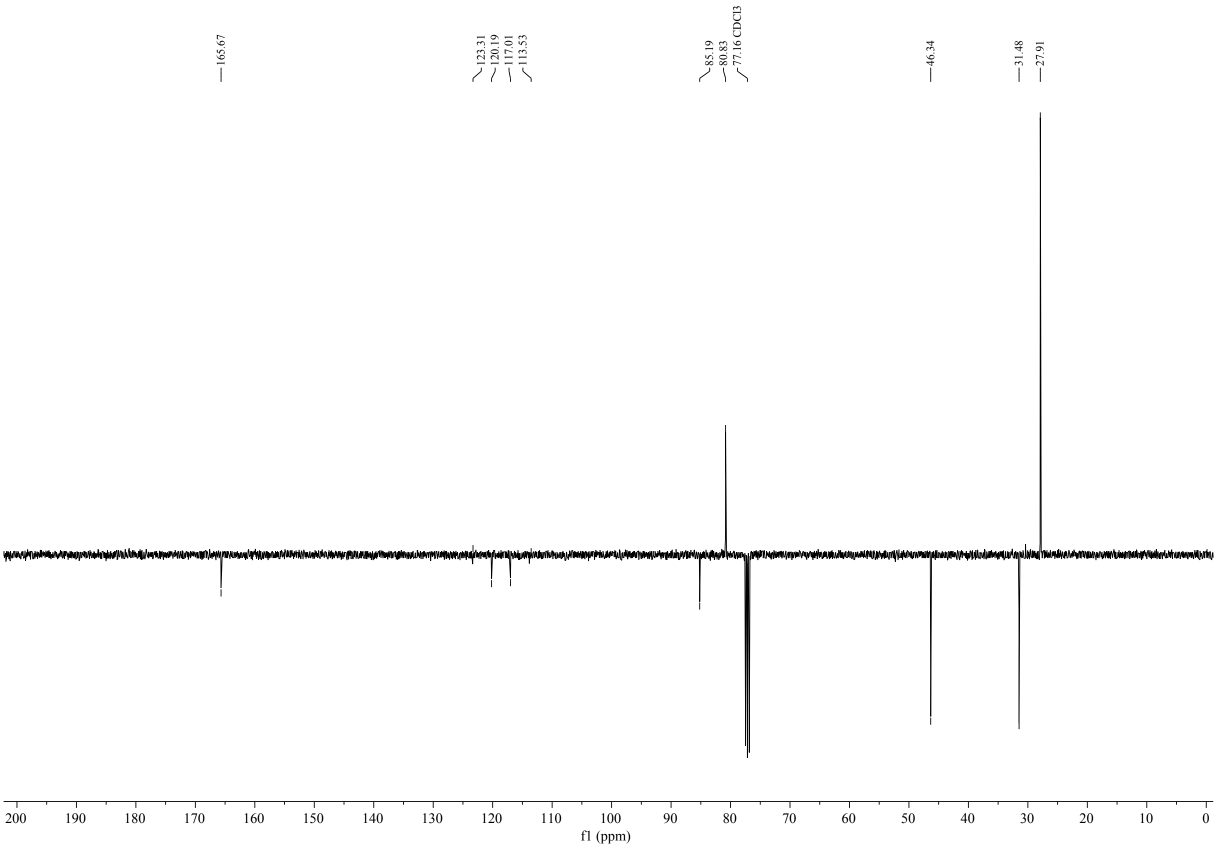

Figure S7: ^{13}C-DEPTQ-135 spectrum (100 MHz, $CDCl_3$) of (*S*)-triflate **4**.

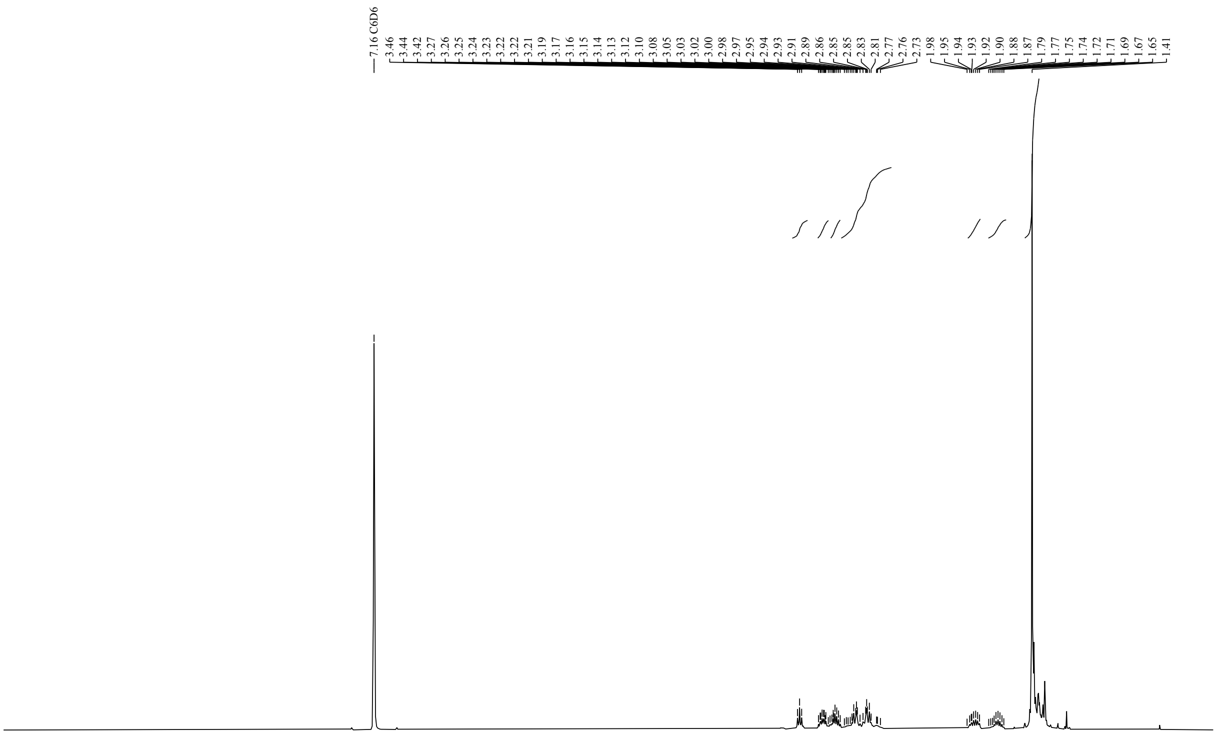

Figure S8: ^{1}H-NMR spectrum (400 MHz, C_6D_6) of $^{t}Bu_4$-DOTAZA **5**.

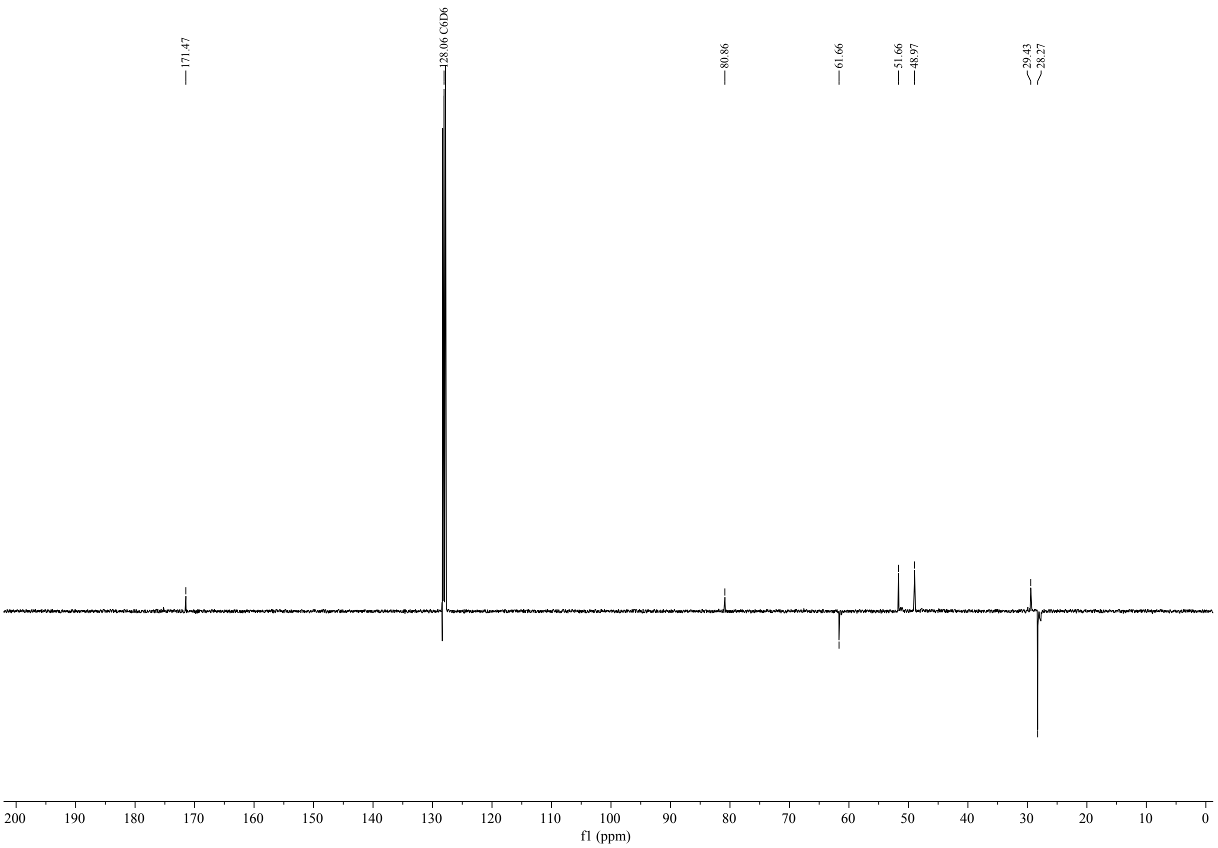

Figure S9: ^{13}C-DEPTQ-135 spectrum (100 MHz, C_6D_6) of ^{t}Bu$_4$-DOTAZA **5**.

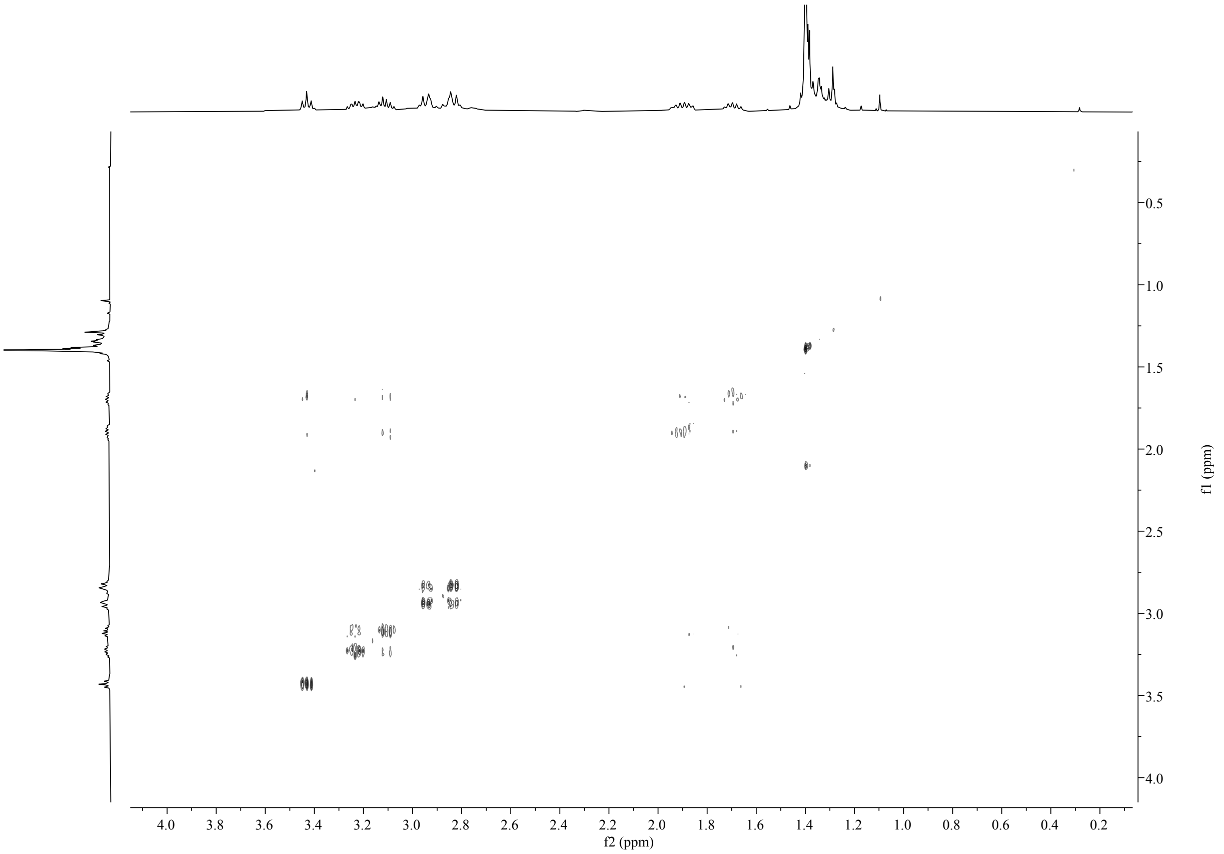

Figure S10: COSY 2D-NMR spectrum (400 MHz, C_6D_6) of ^{t}Bu$_4$-DOTAZA **5.**

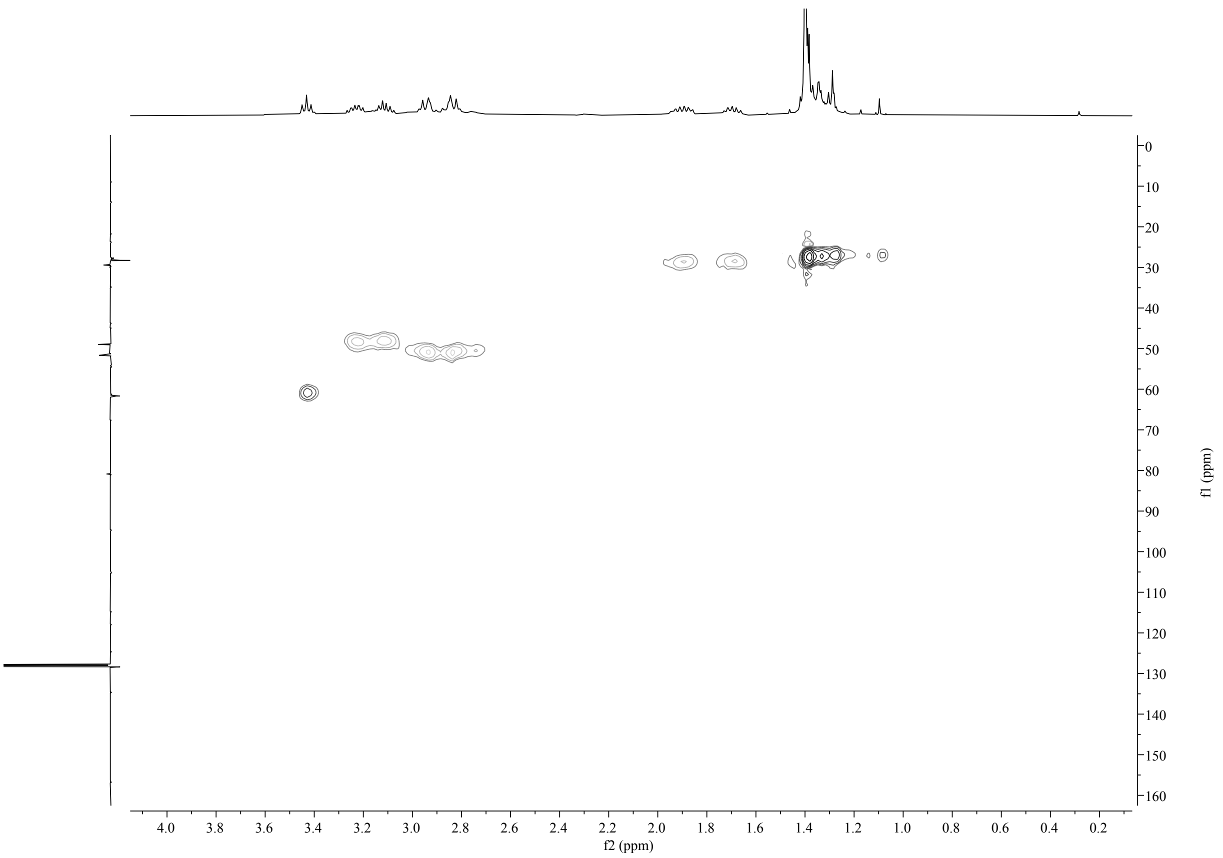

Figure S11: HSQC 2D-NMR spectrum (400 MHz, 100 MHz, C_6D_6) of ^{t}Bu$_4$-DOTAZA **5.**

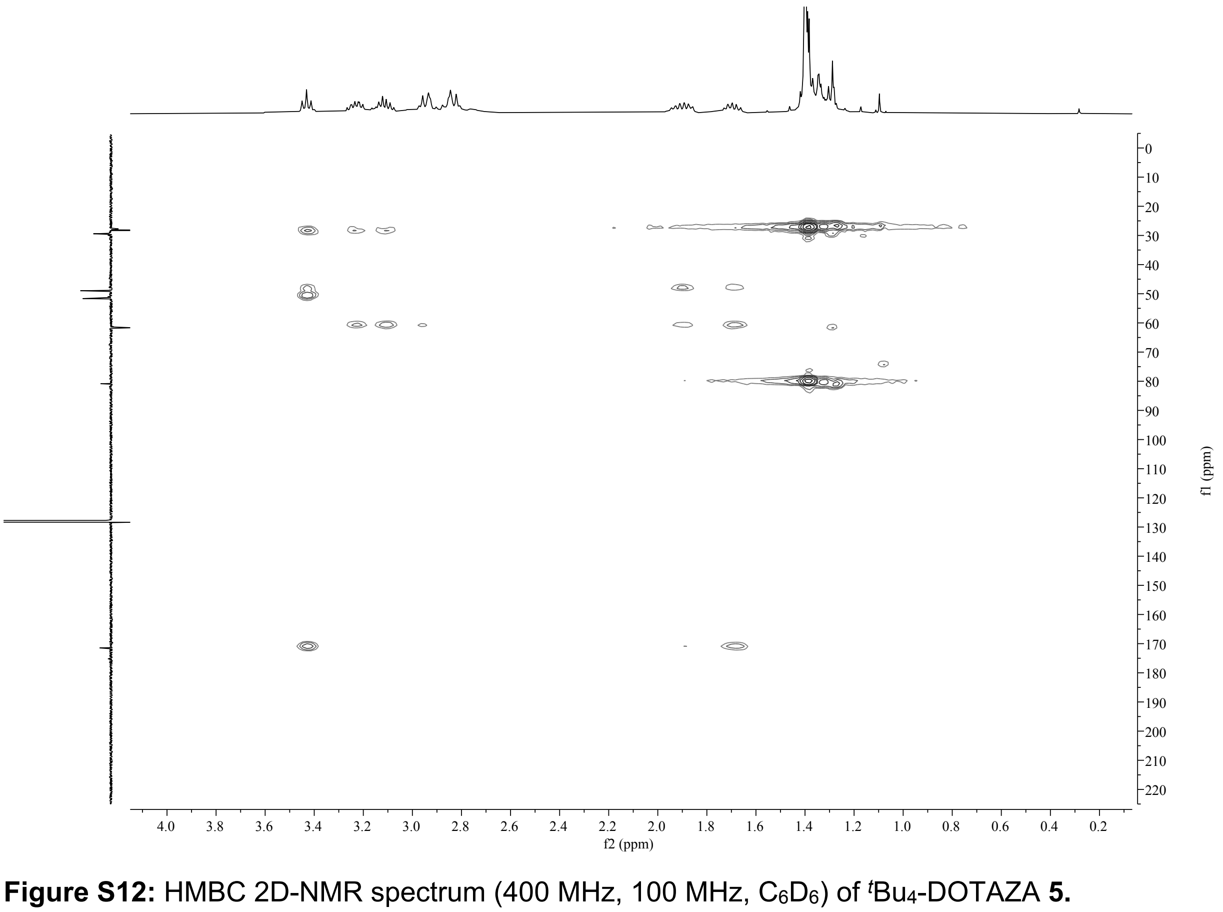

Figure S12: HMBC 2D-NMR spectrum (400 MHz, 100 MHz, C_6D_6) of ^{t}Bu$_4$-DOTAZA **5.**

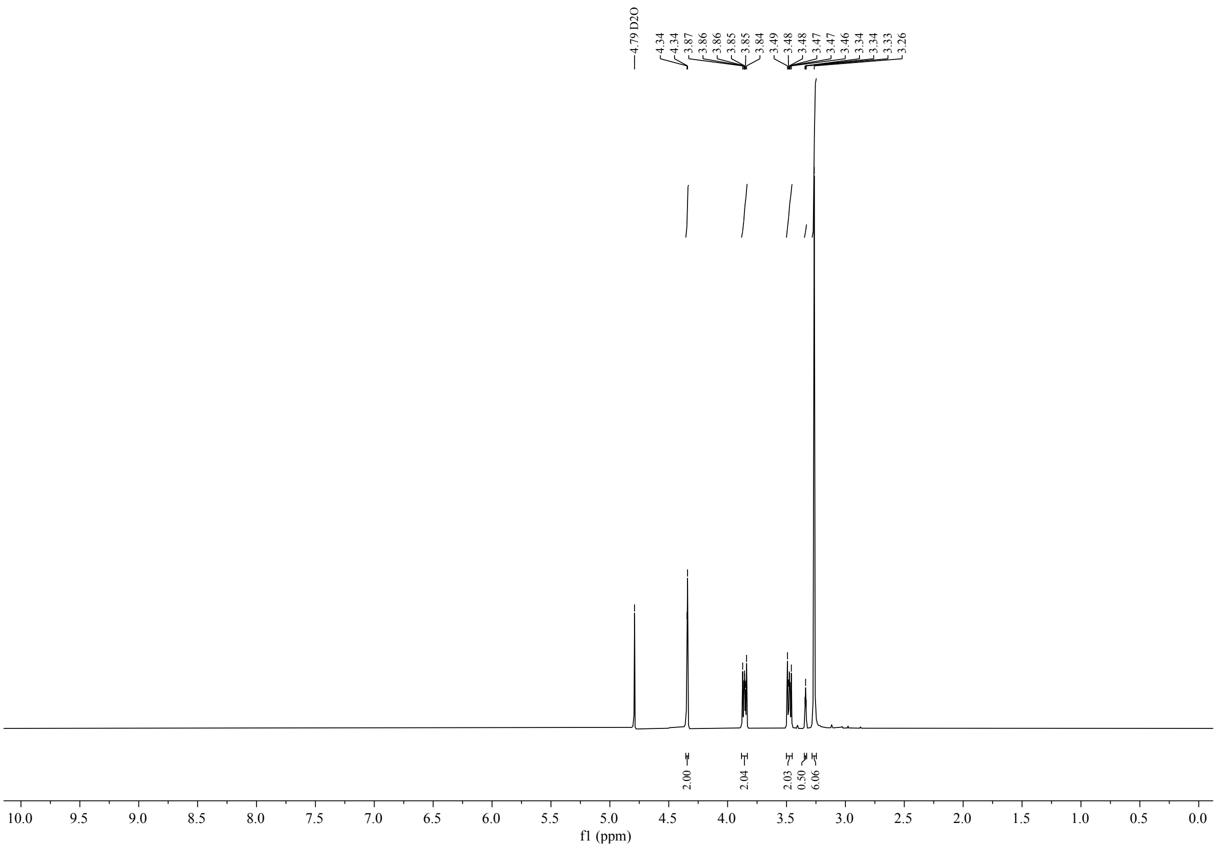

Figure S13: ^{1}H-NMR spectrum (500 MHz, D_2O) of C_2-alkyne **13**.

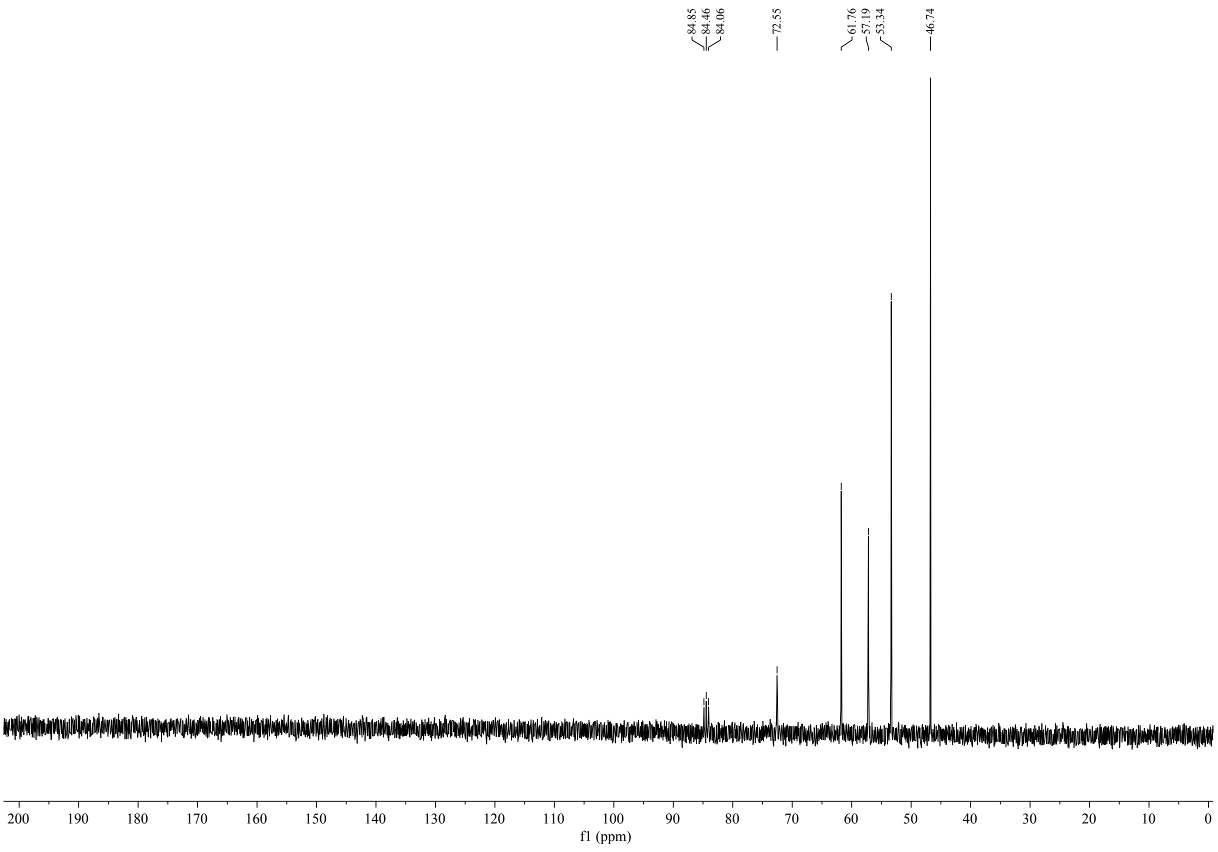

Figure S14: ^{13}C-NMR spectrum (100 MHz, D_2O) of C_2-alkyne **13**.

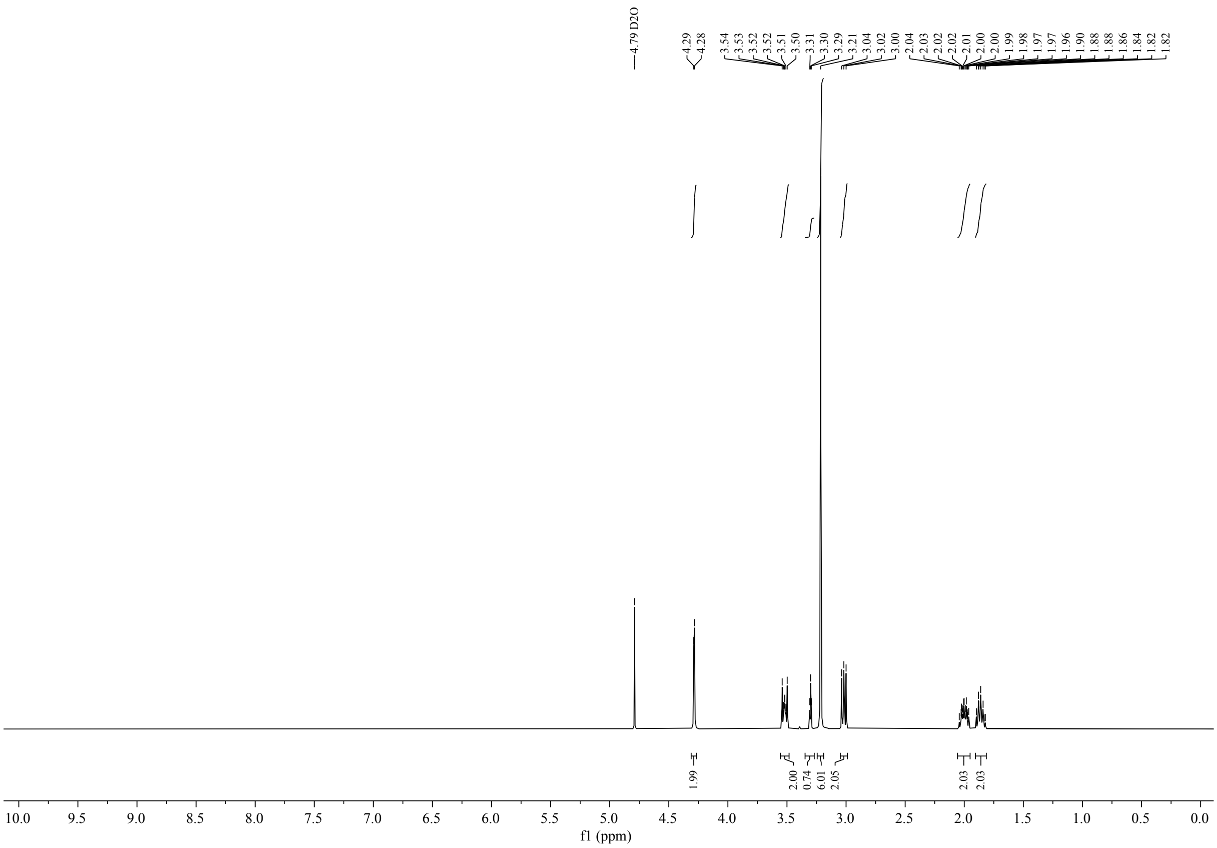

Figure S15: ^{1}H-NMR spectrum (400 MHz, D_2O) of C_4-alkyne **17**.

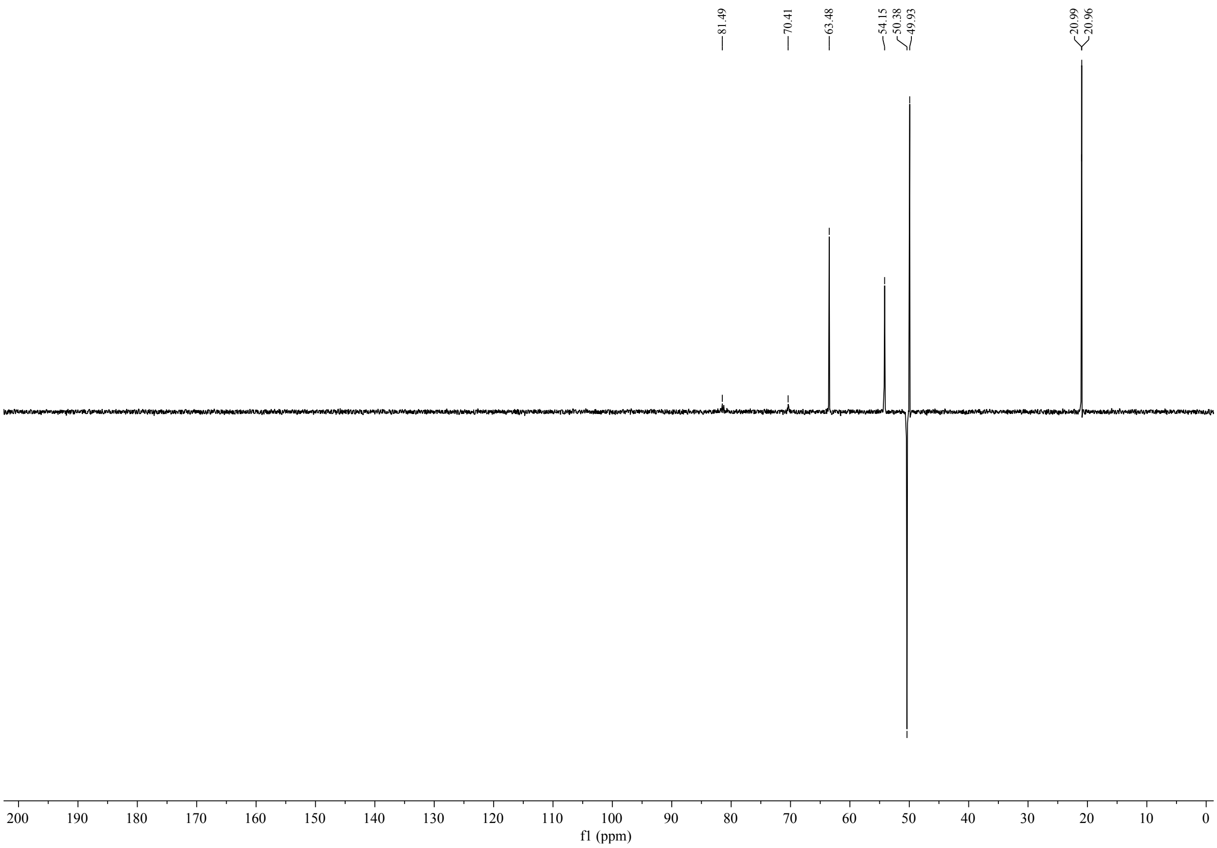

Figure S16: ^{13}C-DEPTQ-135 spectrum (75 MHz, D_2O) of C_4-alkyne **17**.

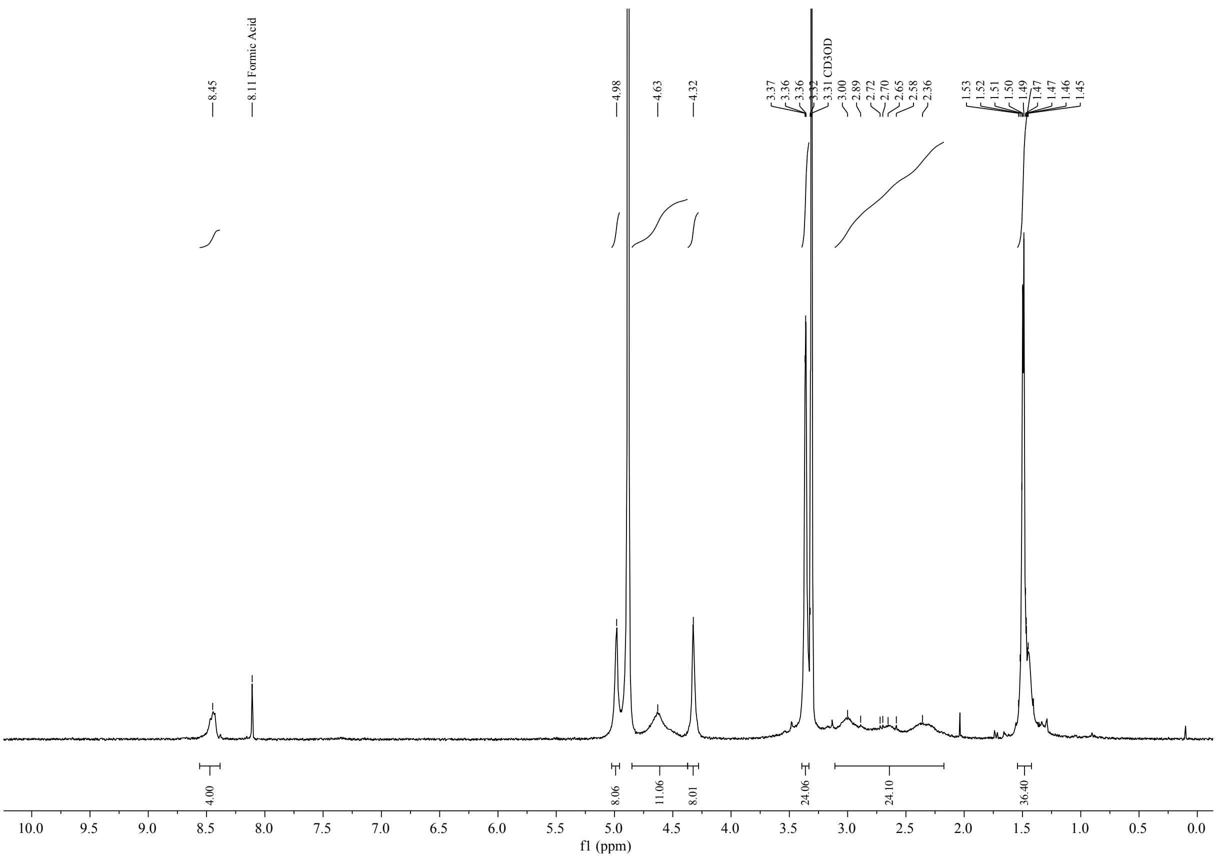

Figure S17: ^{1}H-NMR spectrum (400 MHz, CD_3OD) of $^{t}Bu_4$-C_1ZW-DOTA **6a**.

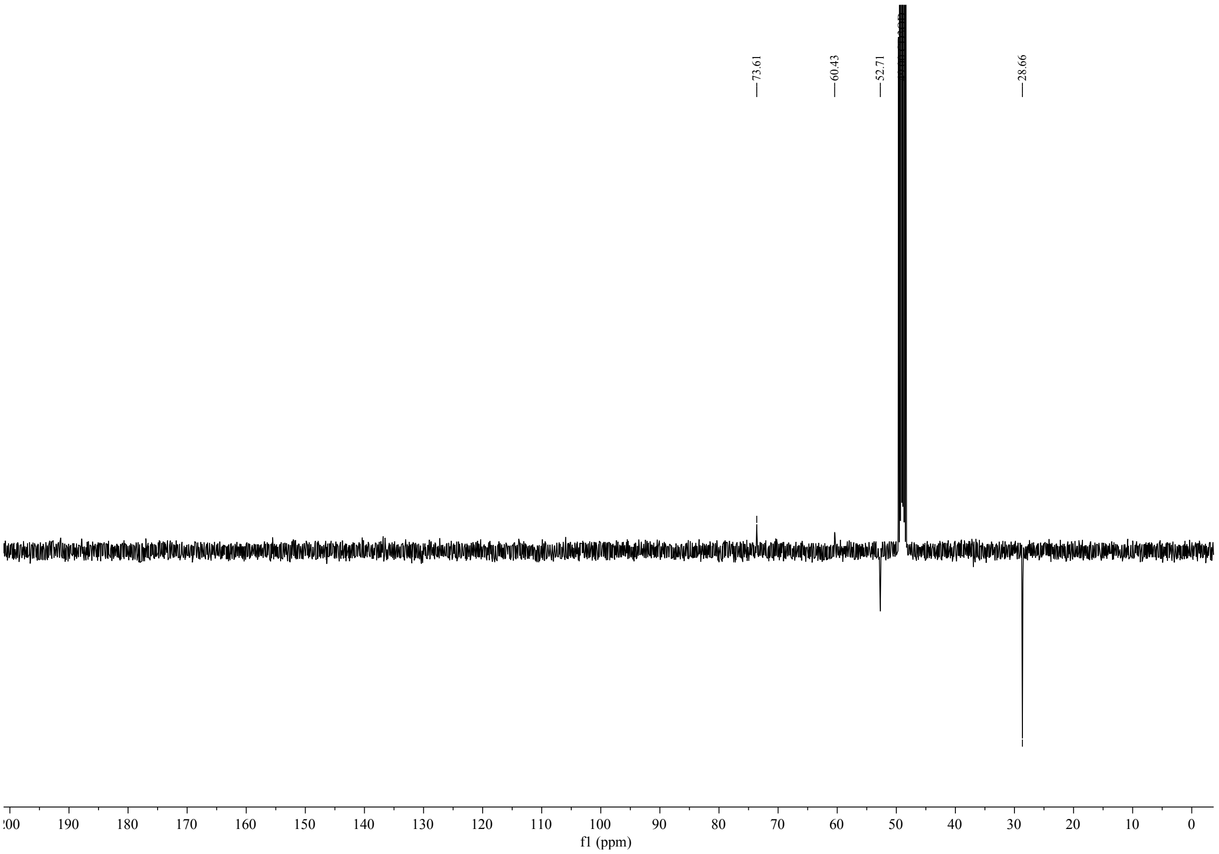

Figure S18: ^{13}C-DEPTQ-135 spectrum (100 MHz, CD_3OD) of $^{t}Bu_4$-C_1ZW-DOTA **6a**.

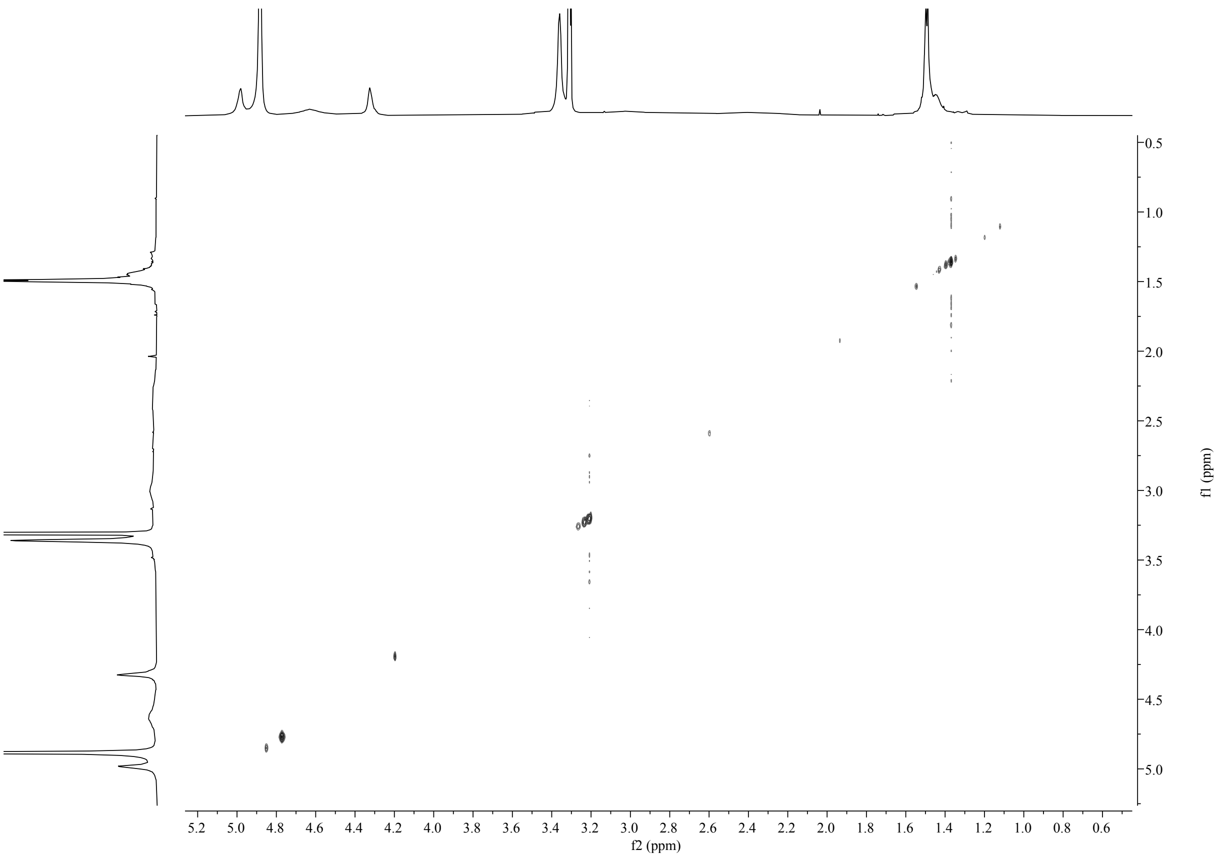

Figure S19: COSY 2D-NMR spectrum (400 MHz, CD_3OD) of ^{t}Bu$_4$-C$_1$ZW-DOTA **6a**.

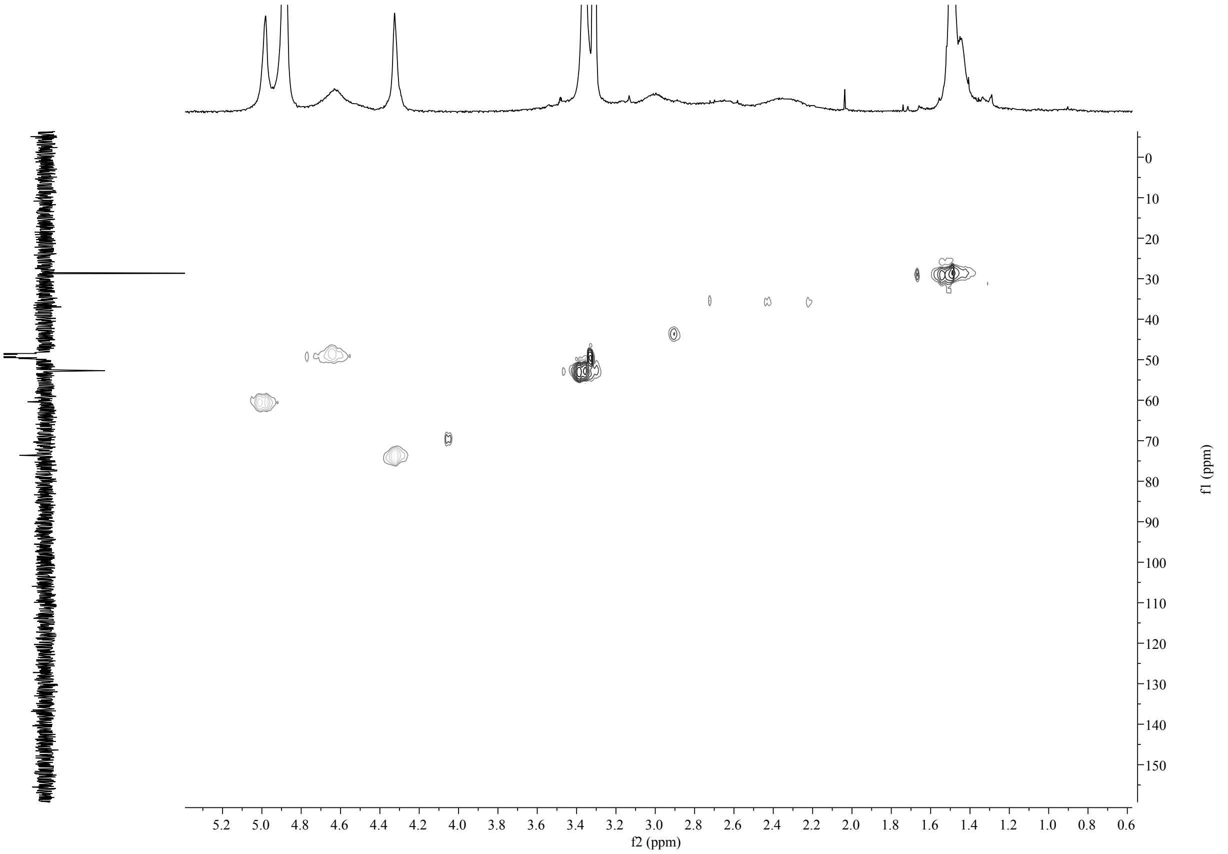

Figure S20: HSQC 2D-NMR spectrum (500 MHz, 125 MHz, CD_3OD) of ^{t}Bu$_4$-C$_1$ZW-DOTA **6a**.

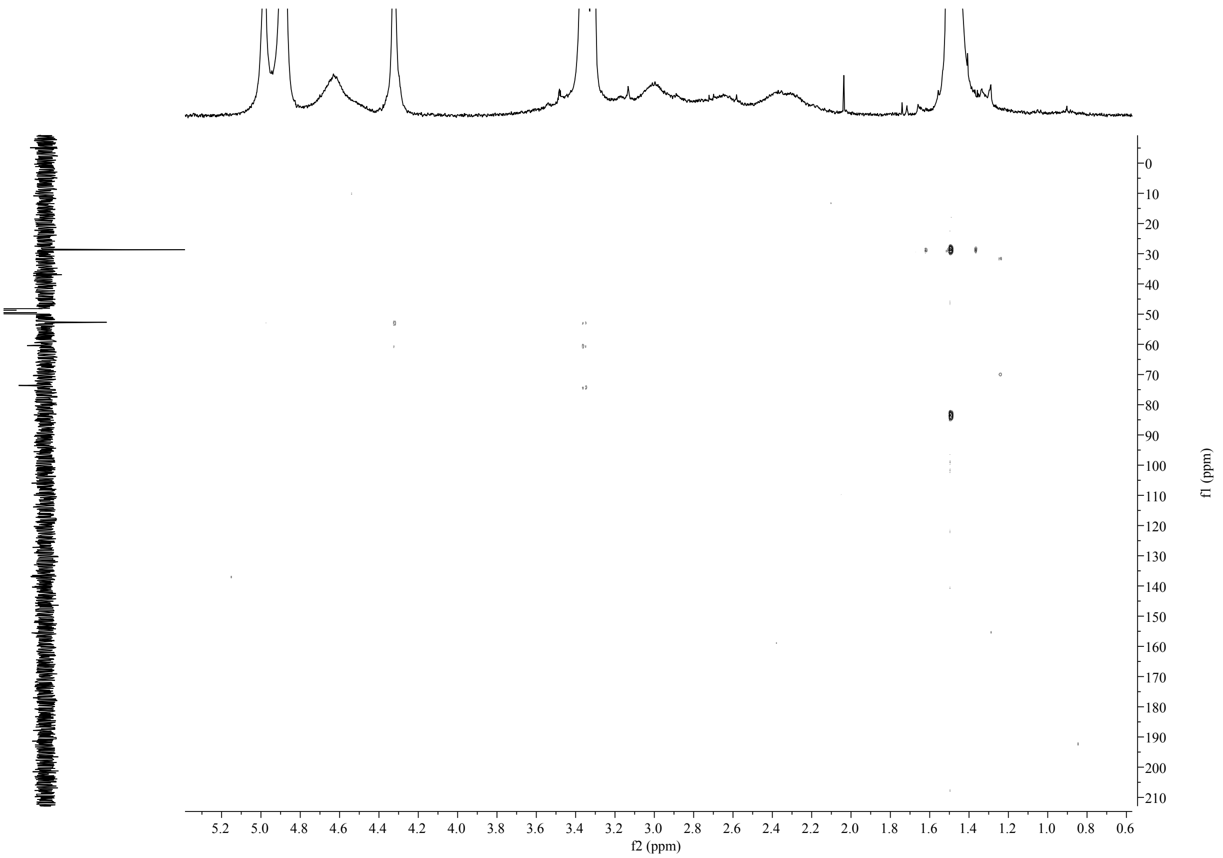

Figure S21: HMBC 2D-NMR spectrum (500 MHz, 125 MHz, CD_3OD) of ^{t}Bu$_4$-C$_1$ZW-DOTA **6a**.

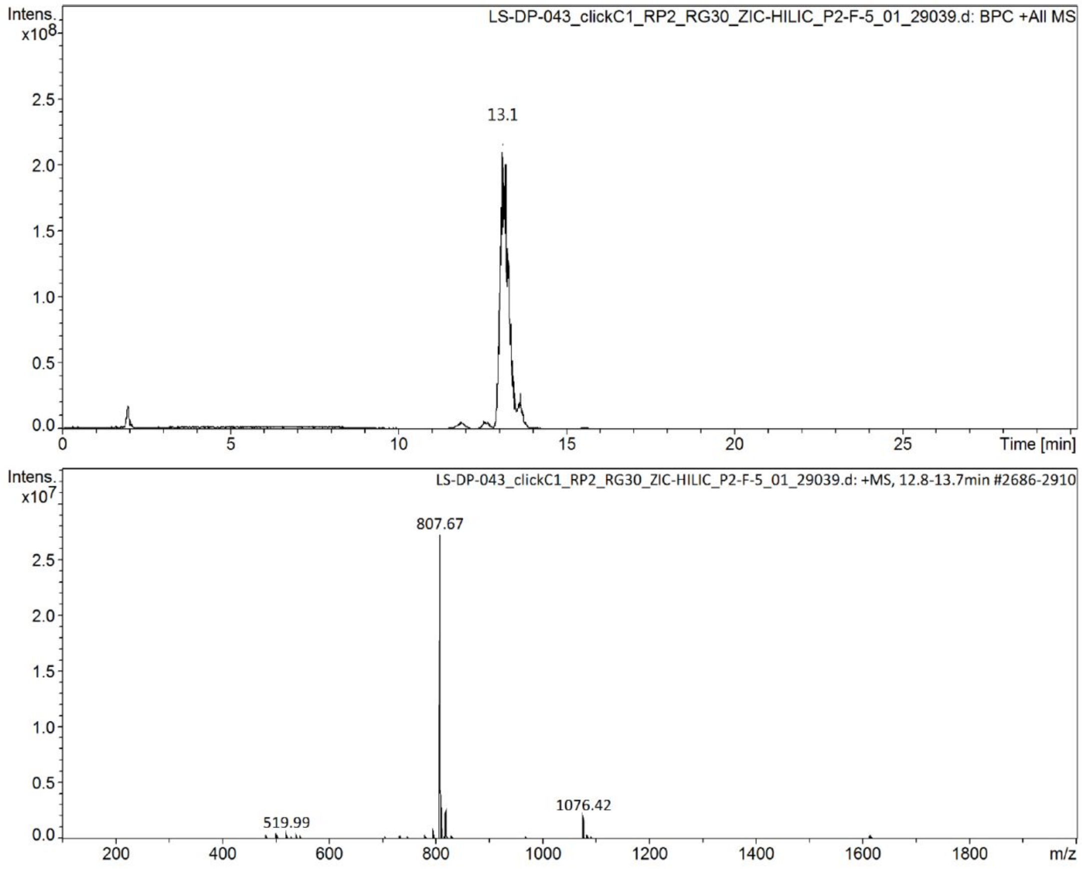

Figure S22: HPLC-MS (SeQuant ZIC-pHILIC, method 1) chromatogram of ^{t}Bu$_4$-C$_1$ZW-DOTA **6a**.

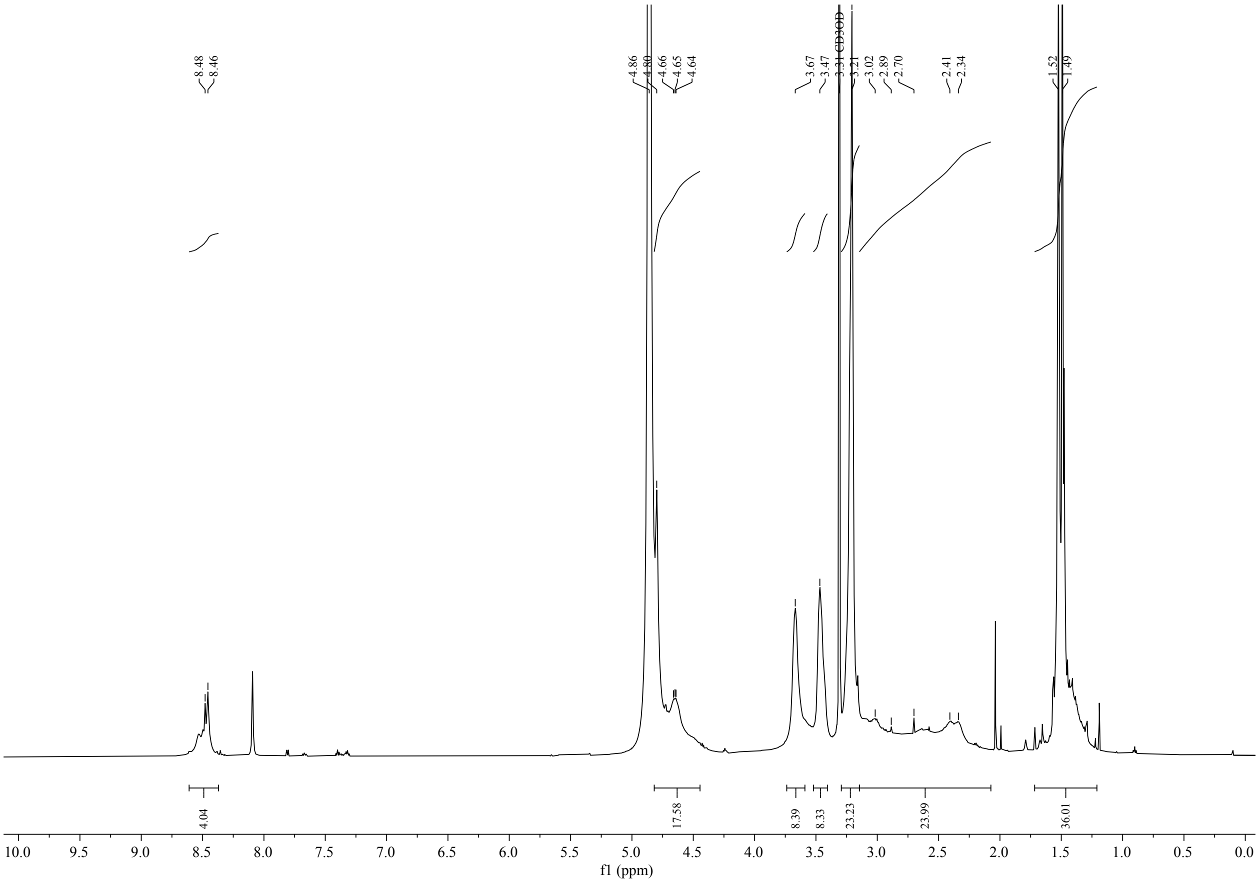

Figure S23: ^{1}H-NMR spectrum (600 MHz, CD_3OD) of $^{t}Bu_4$-C_2ZW-DOTA **6b**.

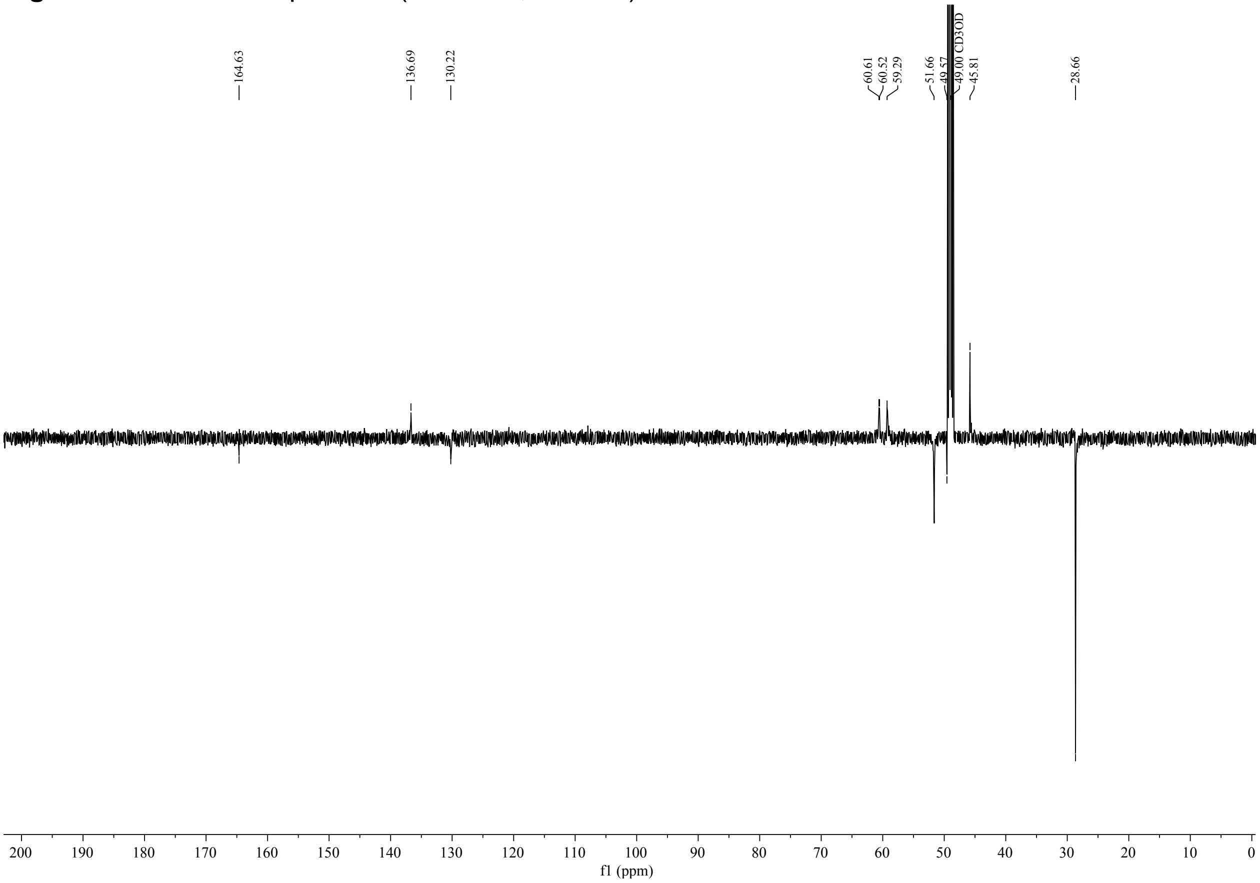

Figure S24: ^{13}C-DEPTQ-135 spectrum (150 MHz, CD_3OD) of $^{t}Bu_4$-C_2ZW-DOTA **6b**.

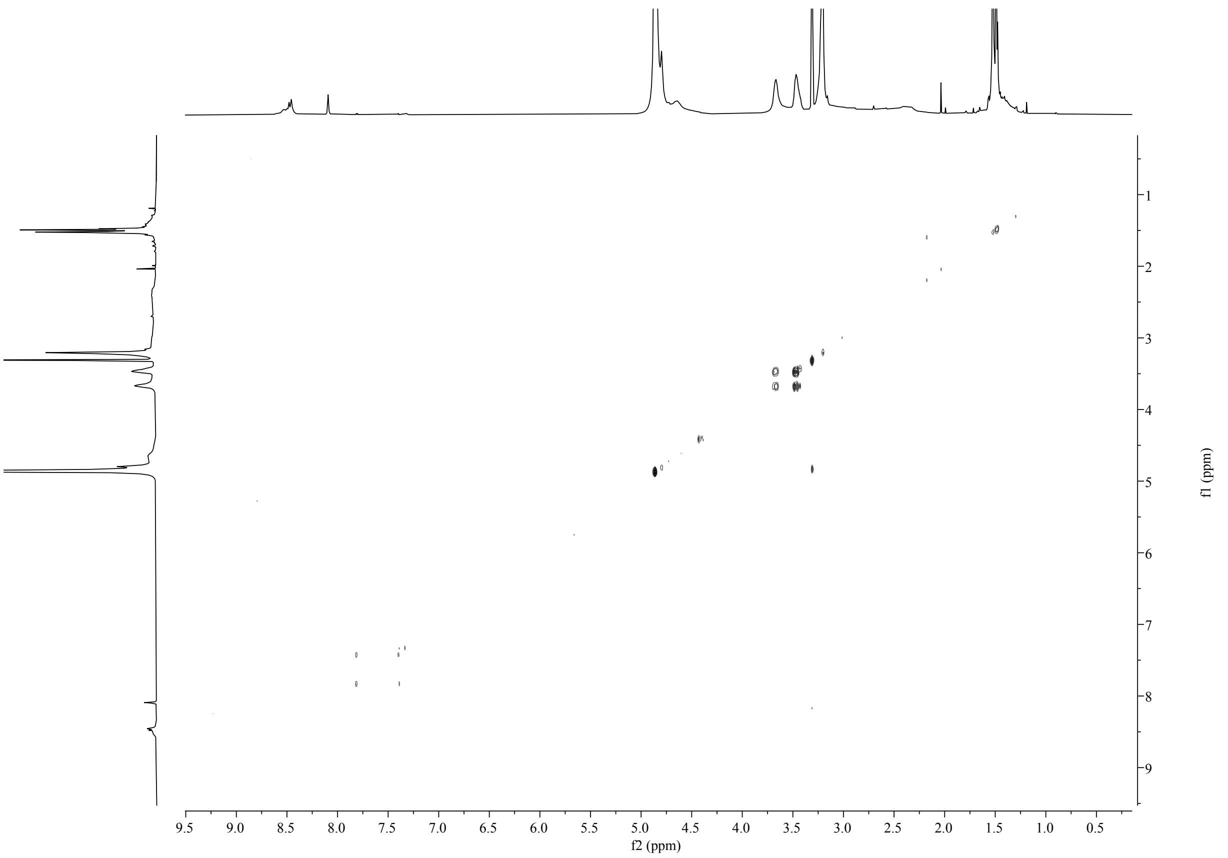

Figure S25: COSY 2D-NMR spectrum (600 MHz, CD_3OD) of ^{t}Bu$_4$-C$_2$ZW-DOTA **6b**.

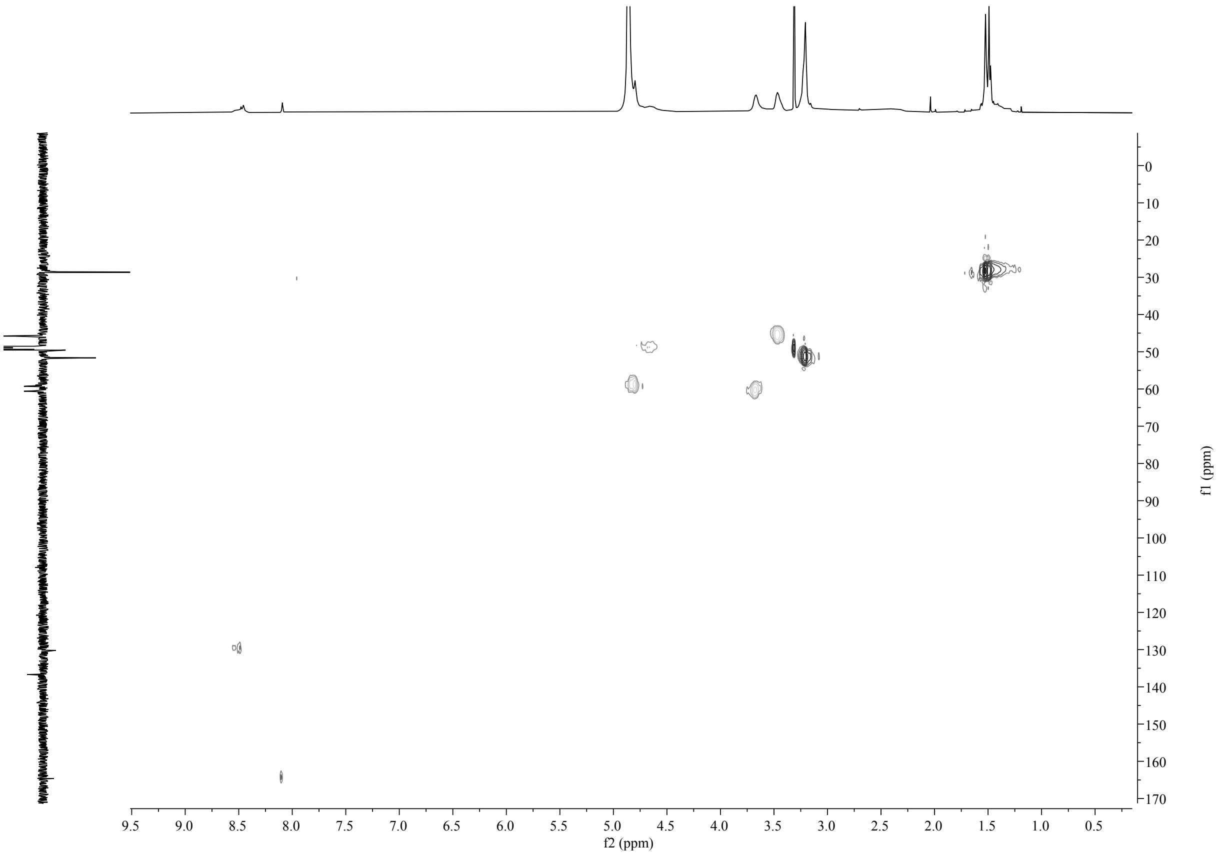

Figure S26: HSQC 2D-NMR spectrum (600 MHz, 150 MHz, CD_3OD) of ^{t}Bu$_4$-C$_2$ZW-DOTA **6b**.

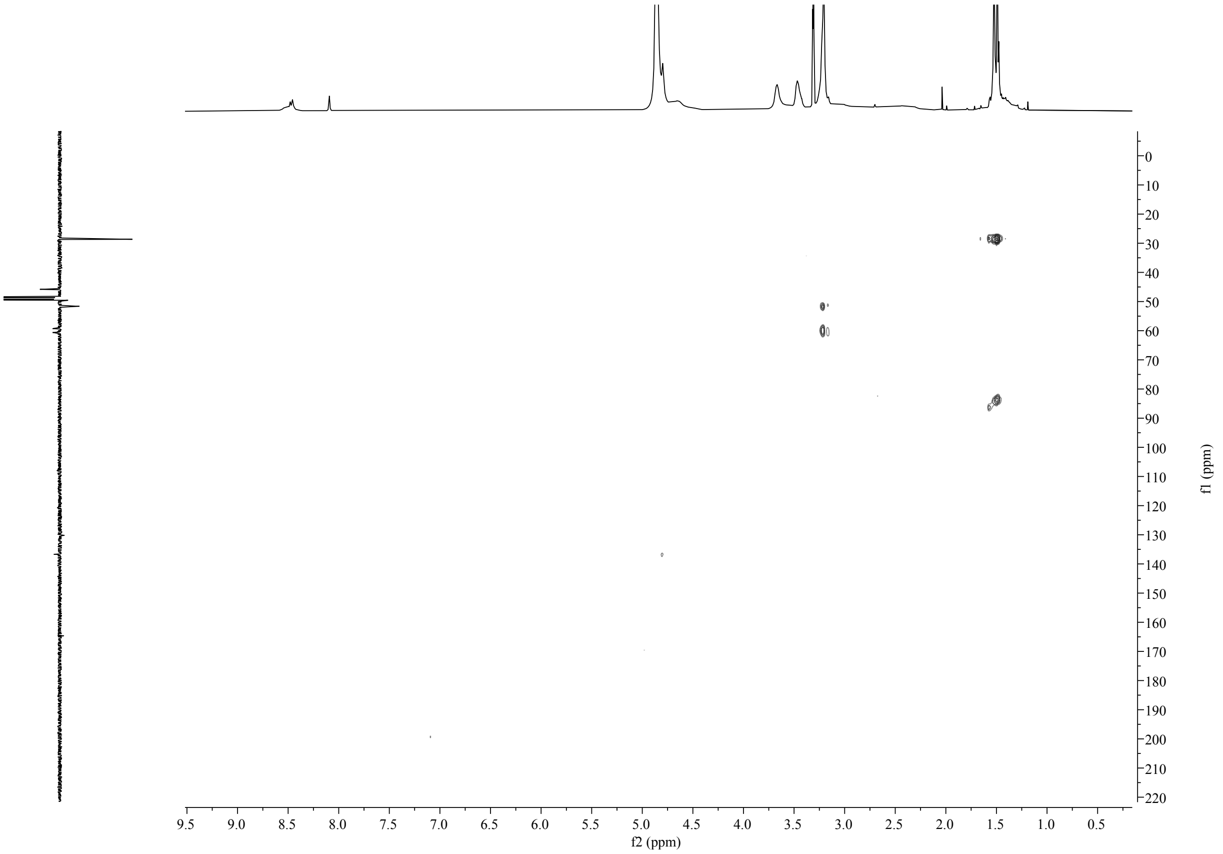

Figure S27: HMBC 2D-NMR spectrum (600 MHz, 150 MHz, CD_3OD) of ^{t}Bu$_4$-C$_2$ZW-DOTA **6b**.

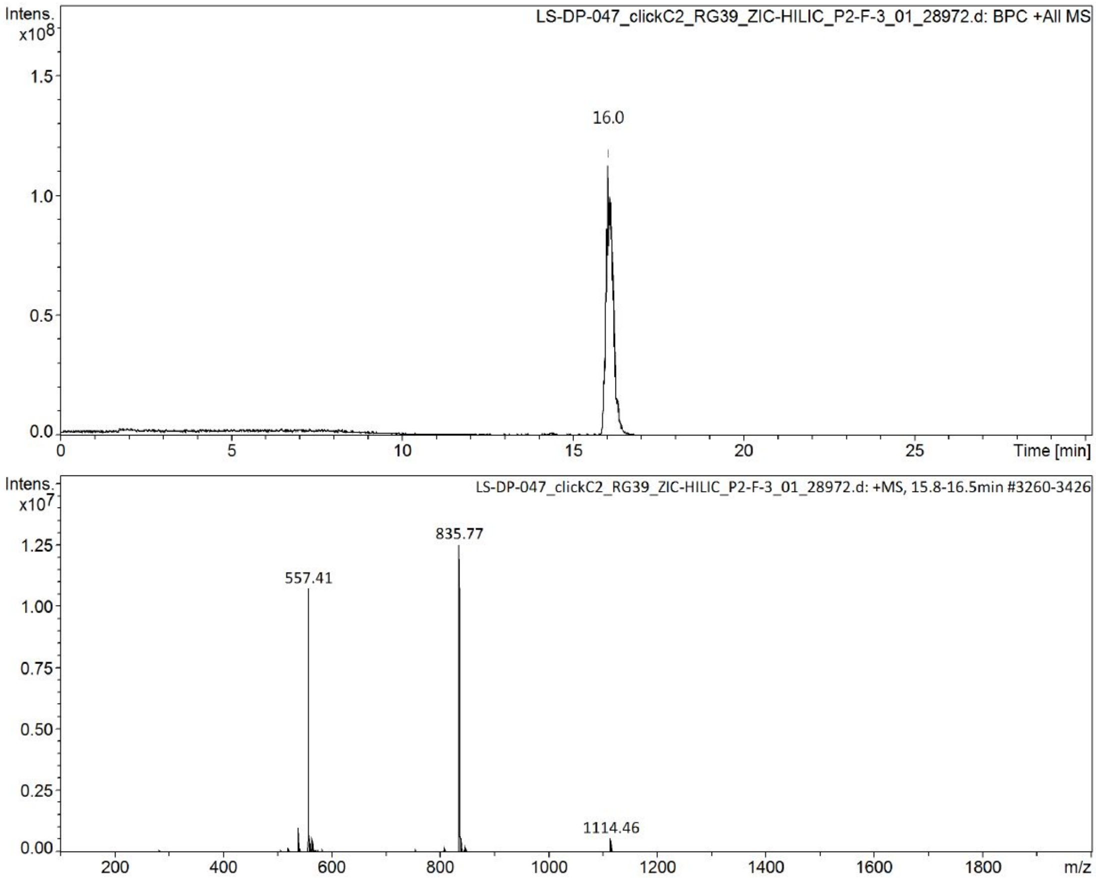

Figure S28: HPLC-MS (SeQuant ZIC-pHILIC, method 1) chromatogram of ^{t}Bu$_4$-C$_2$ZW-DOTA **6b**.

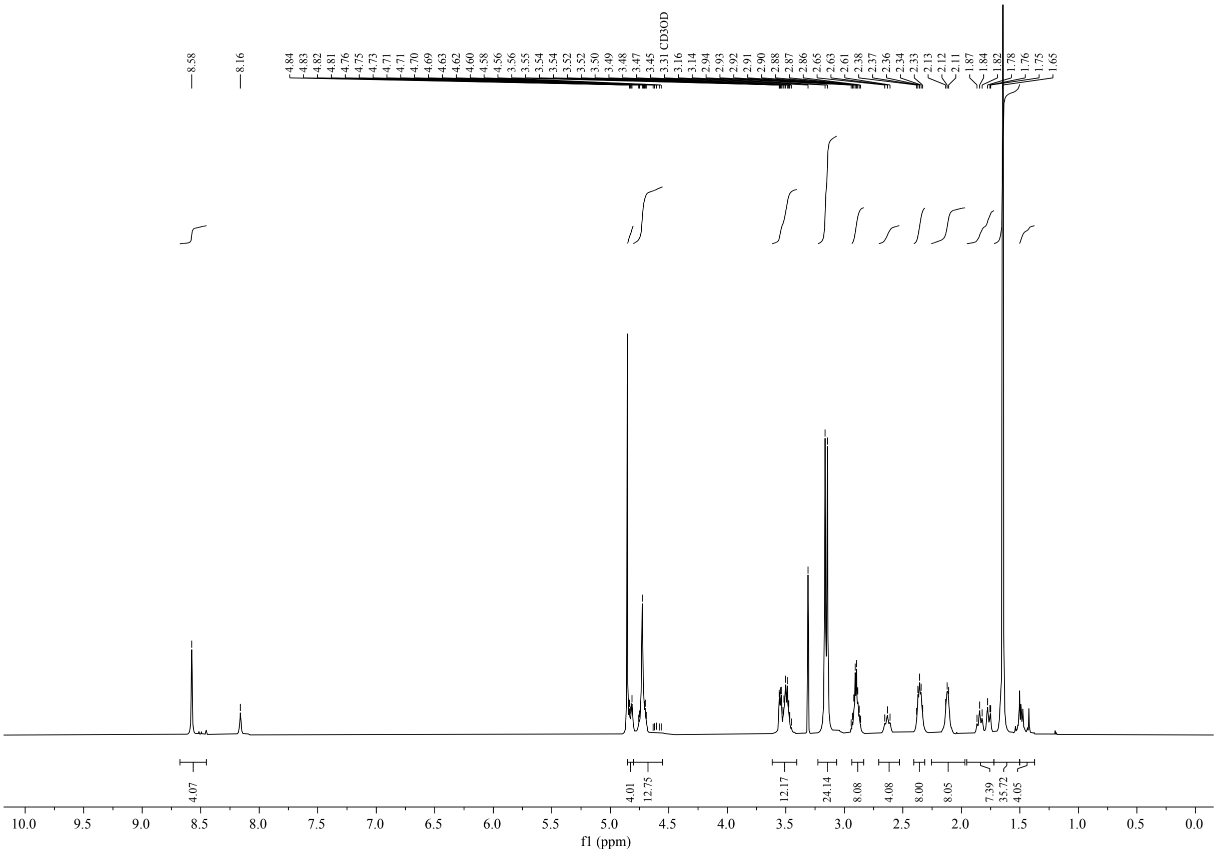

Figure S29: ^{1}H-NMR spectrum (600 MHz, CD_3OD) of $^{t}Bu_4$-C_3ZW-DOTA **6c**.

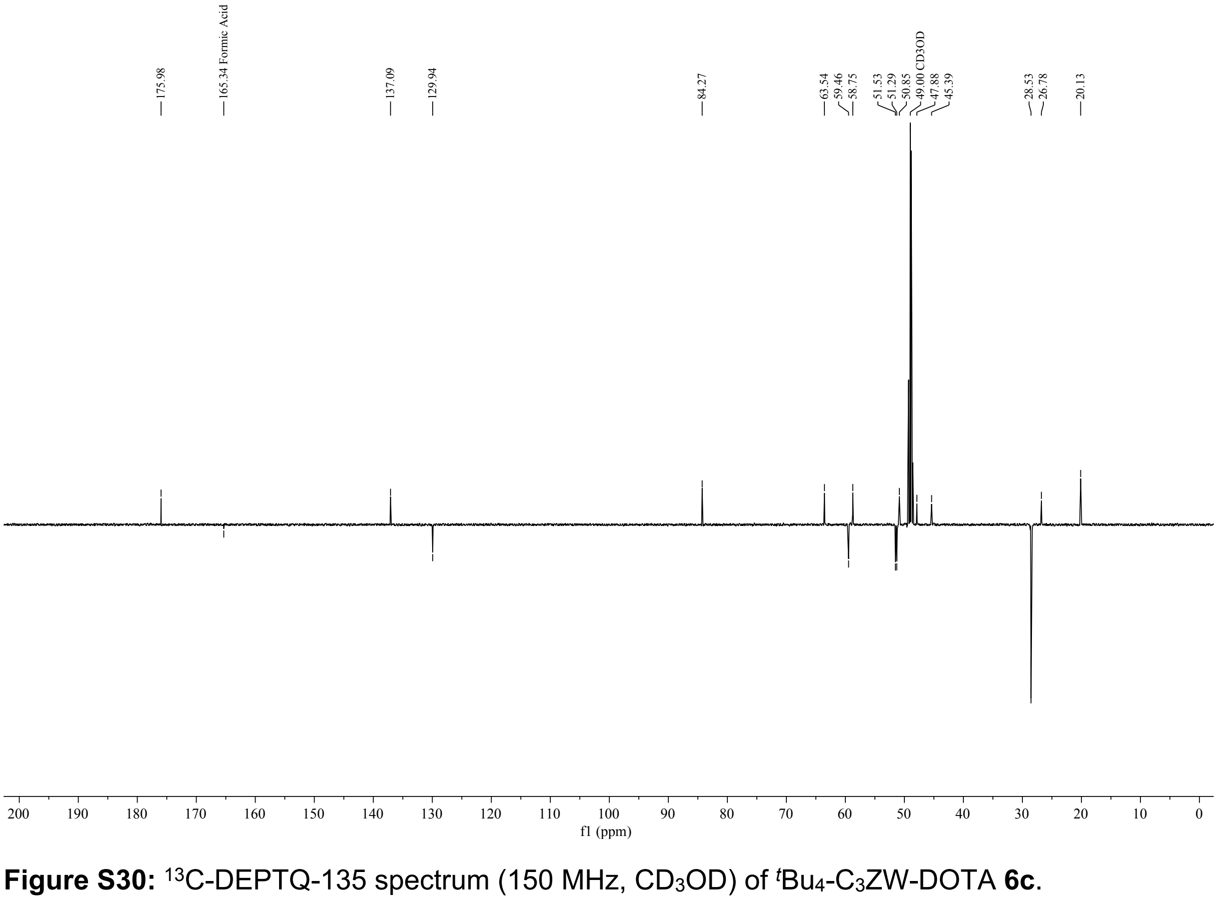

Figure S30: ^{13}C-DEPTQ-135 spectrum (150 MHz, CD_3OD) of $^{t}Bu_4$-C_3ZW-DOTA **6c**.

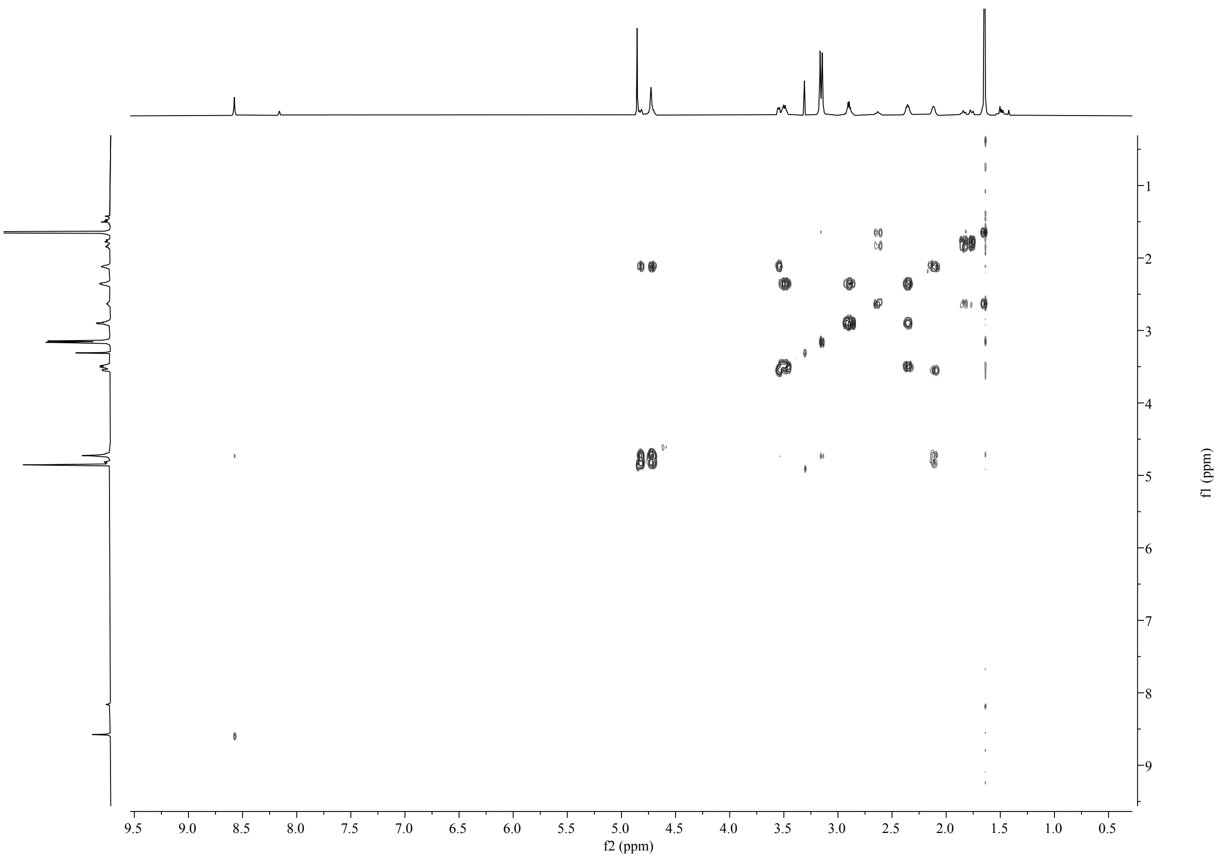

Figure S31: COSY 2D-NMR spectrum (600 MHz, CD_3OD) of ^{t}Bu$_4$-C$_3$ZW-DOTA **6c**.

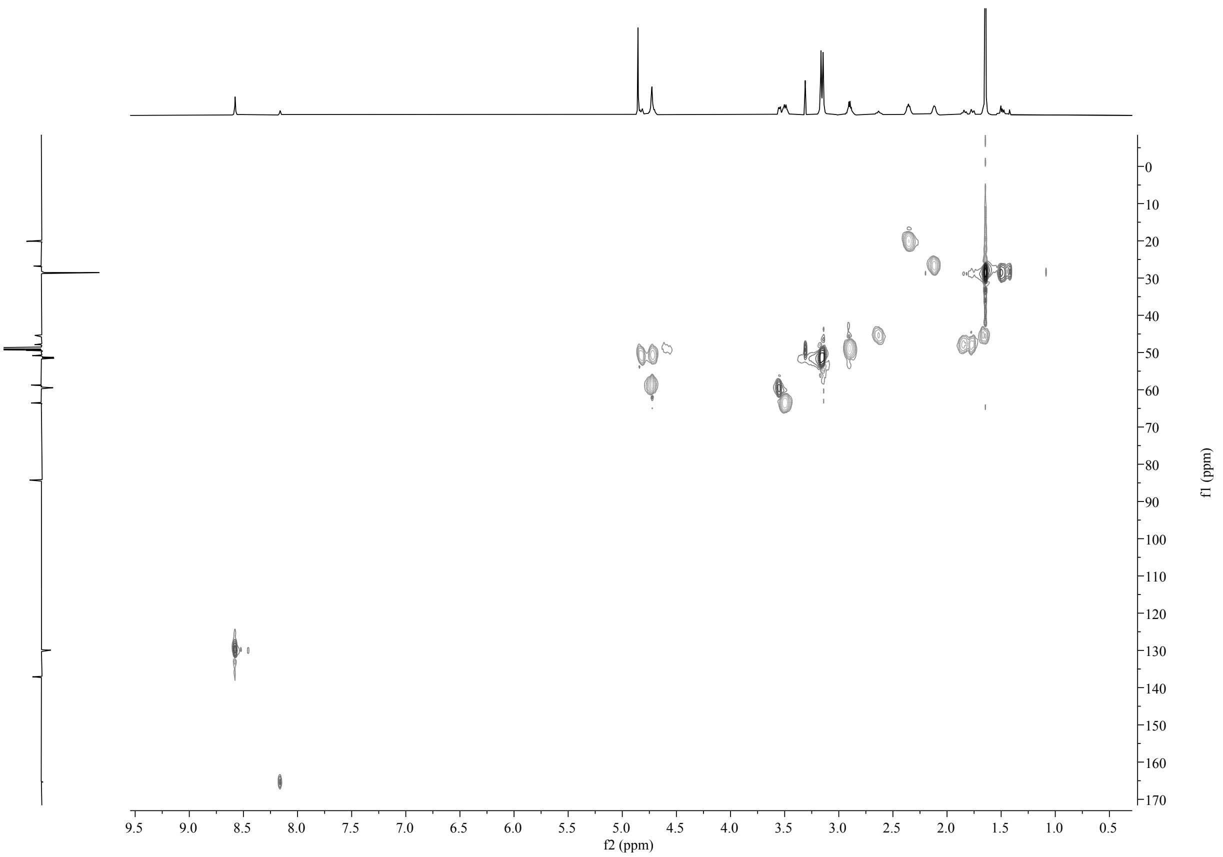

Figure S32: HSQC 2D-NMR spectrum (600 MHz, 150 MHz, CD_3OD) of ^{t}Bu$_4$-C$_3$ZW-DOTA **6c**.

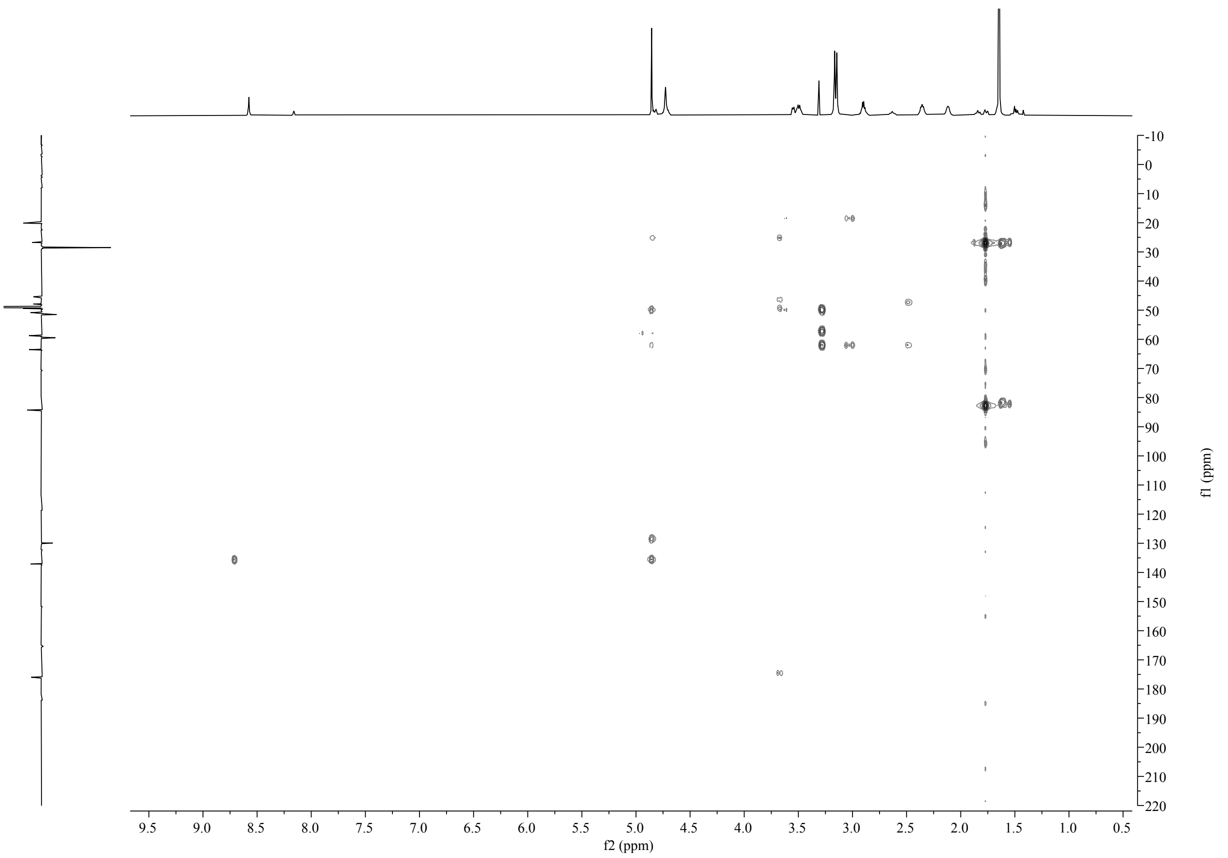

Figure S33: HMBC 2D-NMR spectrum (600 MHz, 150 MHz, CD_3OD) of ^{t}Bu$_4$-C$_3$ZW-DOTA **6c**.

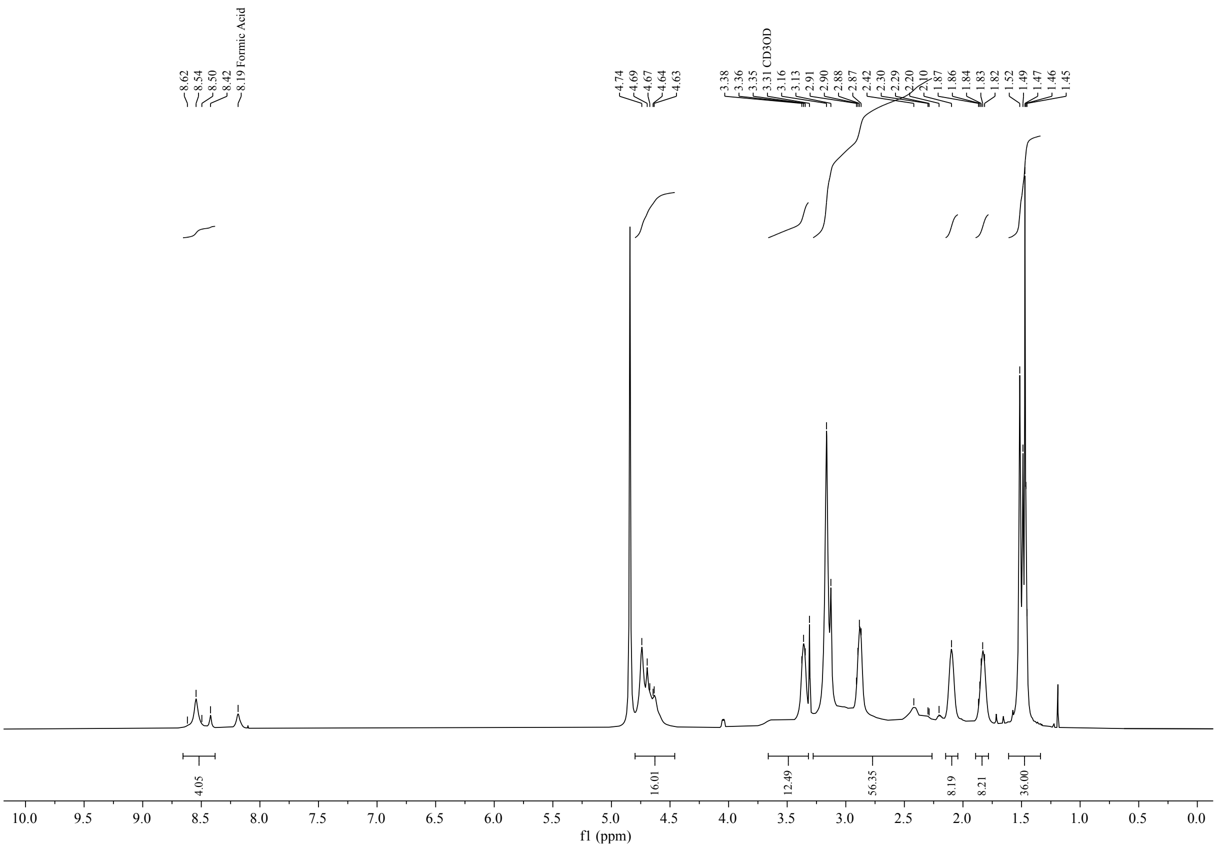

Figure S34: ^{1}H-NMR spectrum (600 MHz, CD_3OD) of ^{t}Bu$_4$-C$_4$ZW-DOTA **6d**.

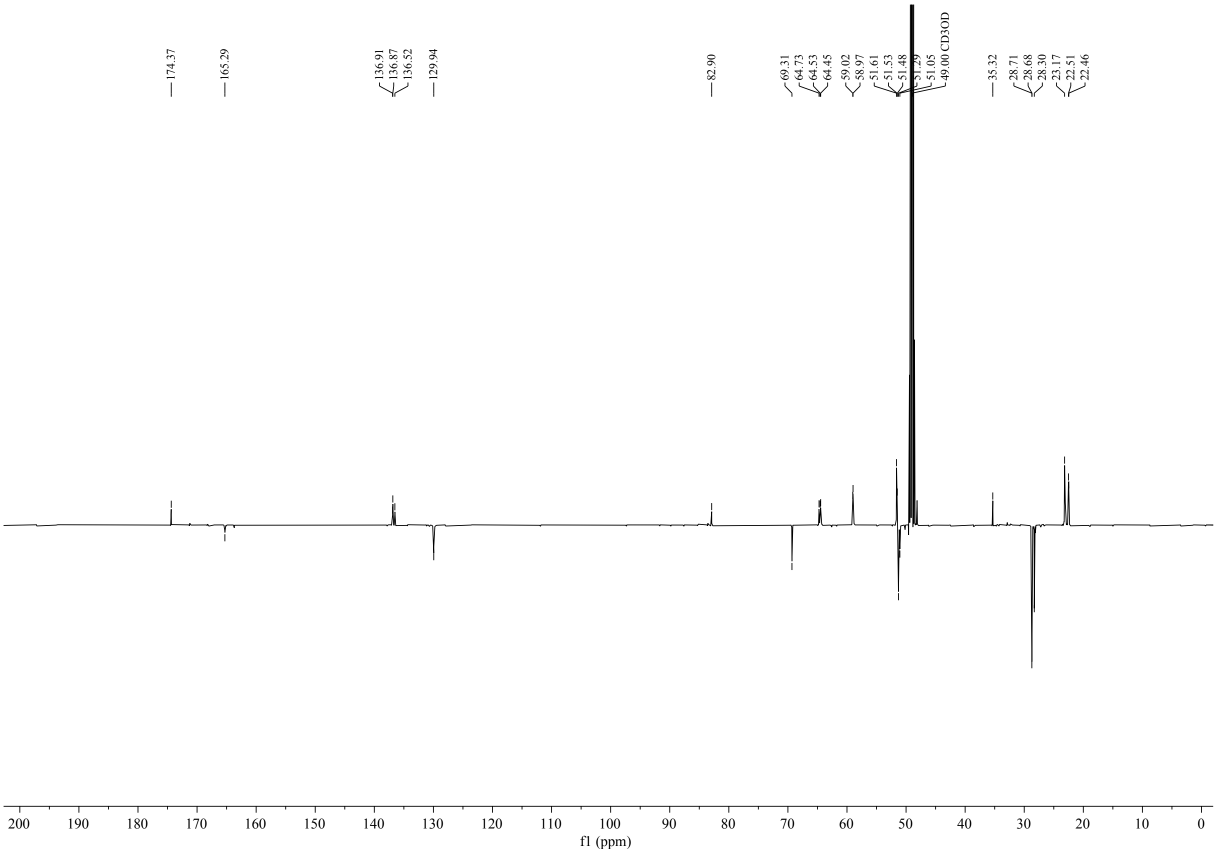

Figure S35: ^{13}C-DEPTQ-135 spectrum (150 MHz, CD_3OD) of ^{t}Bu$_4$-C$_4$ZW-DOTA **6d**.

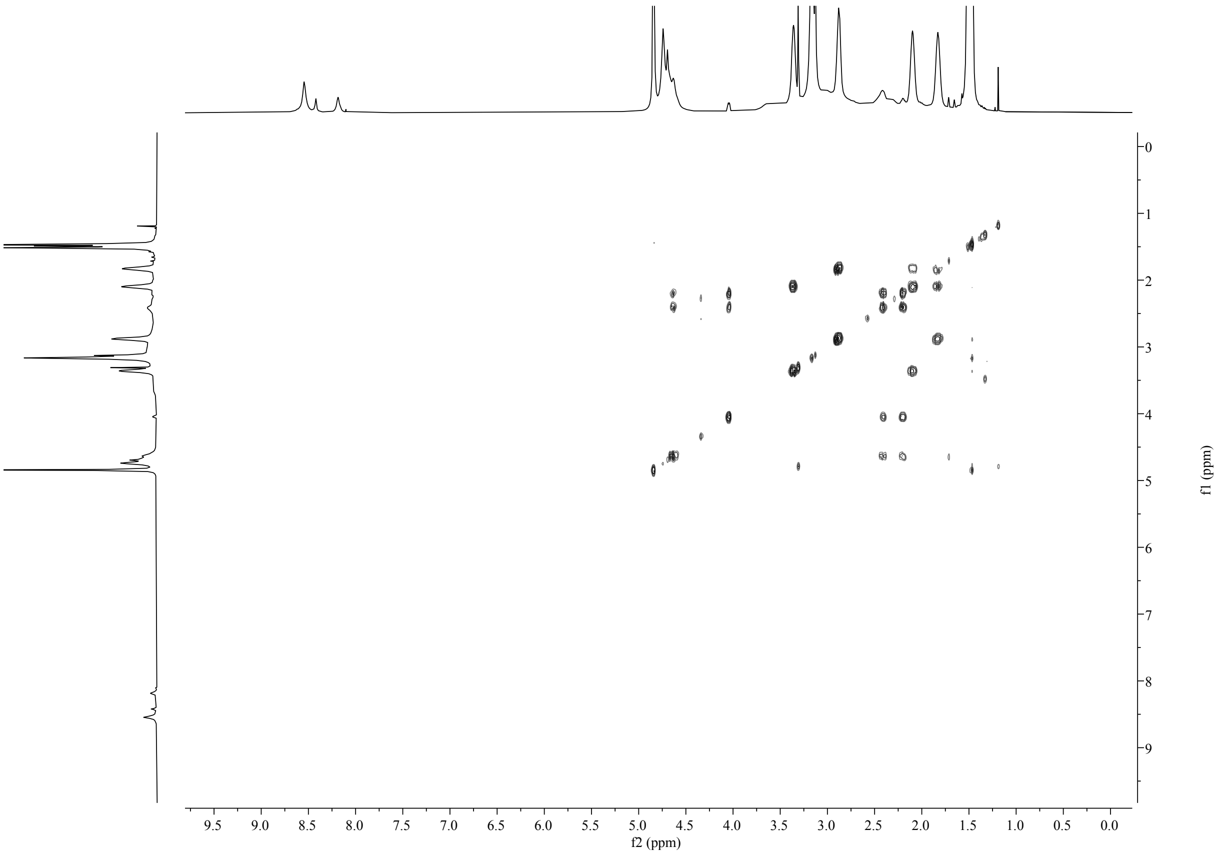

Figure S36: COSY 2D-NMR spectrum (600 MHz, CD_3OD) of ^{t}Bu$_4$-C$_4$ZW-DOTA **6d**.

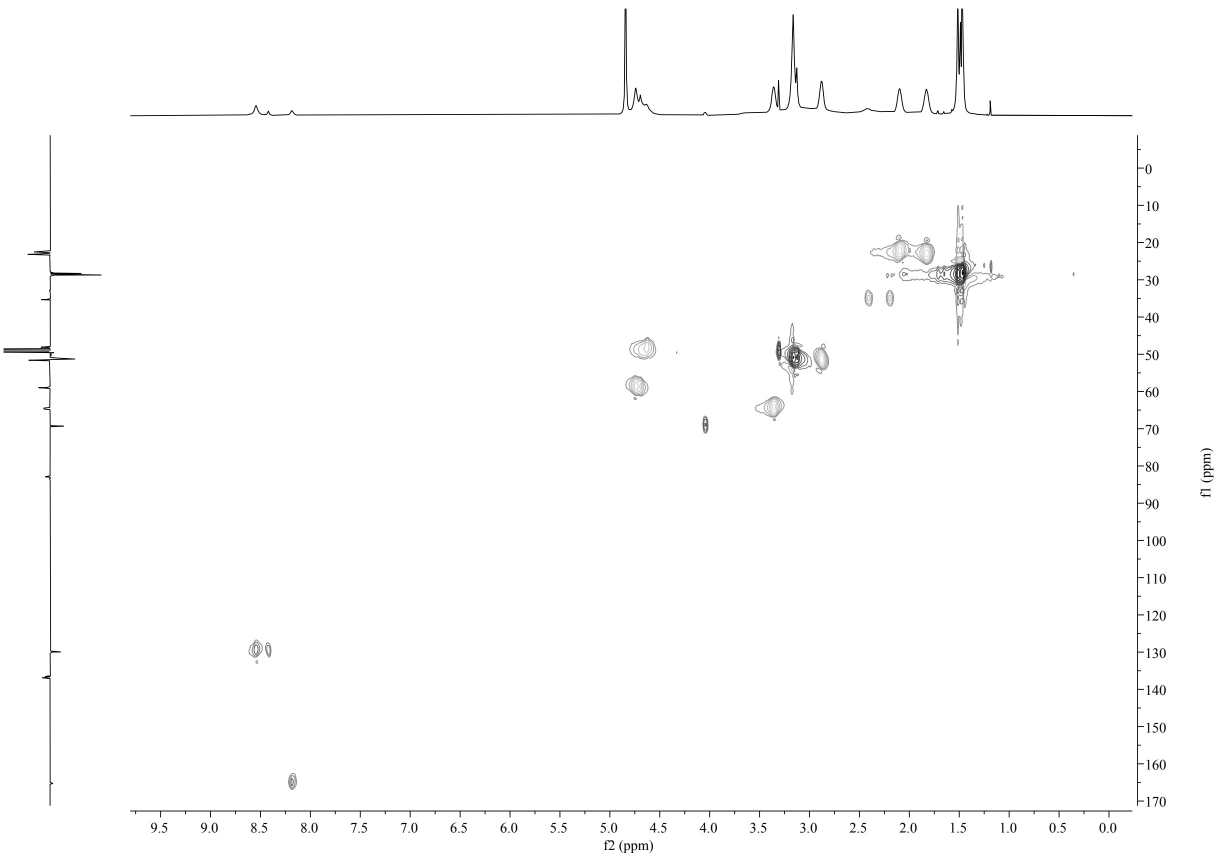

Figure S37: HSQC 2D-NMR spectrum (600 MHz, 150 MHz CD_3OD) of ^{t}Bu$_4$-C$_4$ZW-DOTA **6d**.

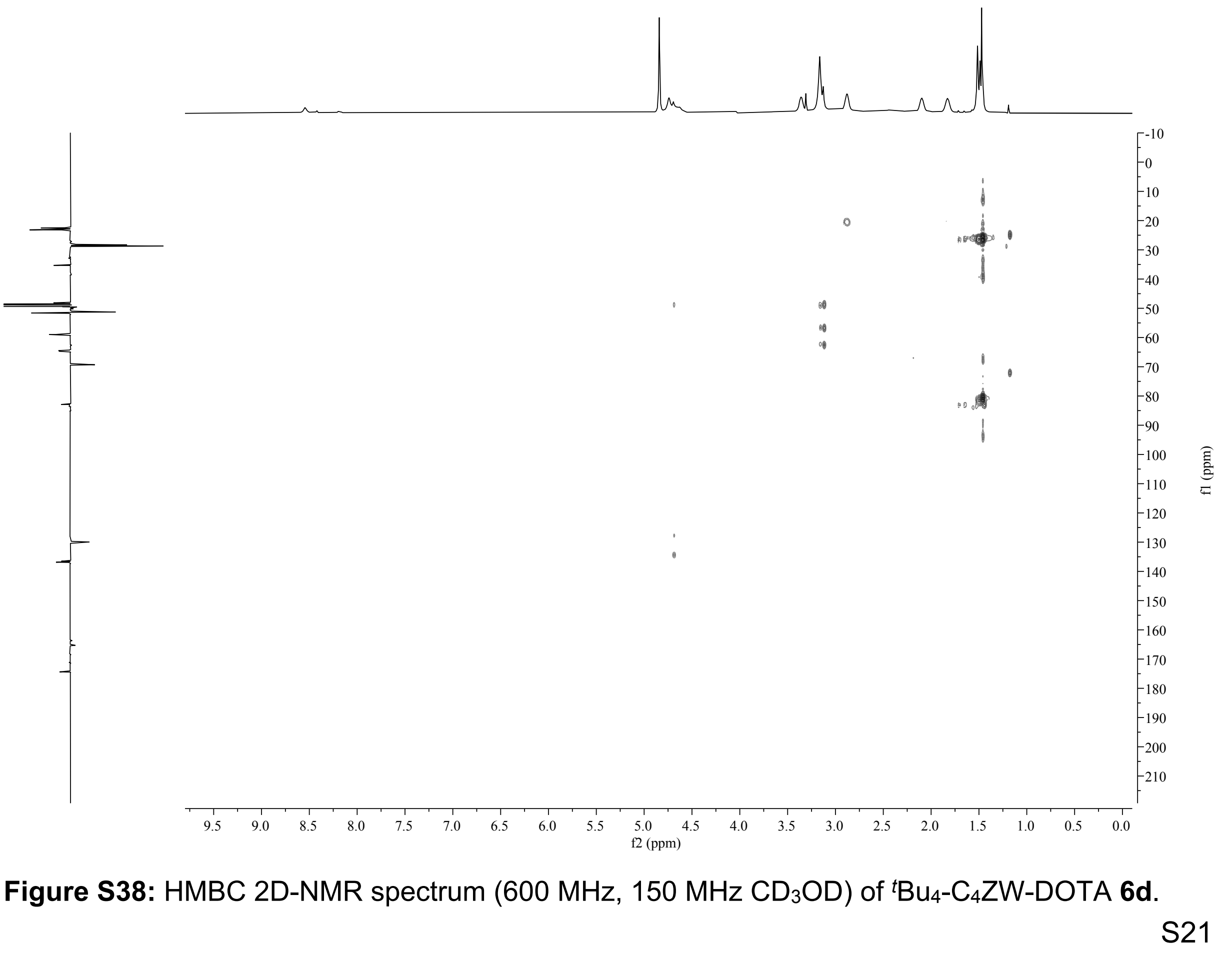

Figure S38: HMBC 2D-NMR spectrum (600 MHz, 150 MHz CD_3OD) of ^{t}Bu$_4$-C$_4$ZW-DOTA **6d**.

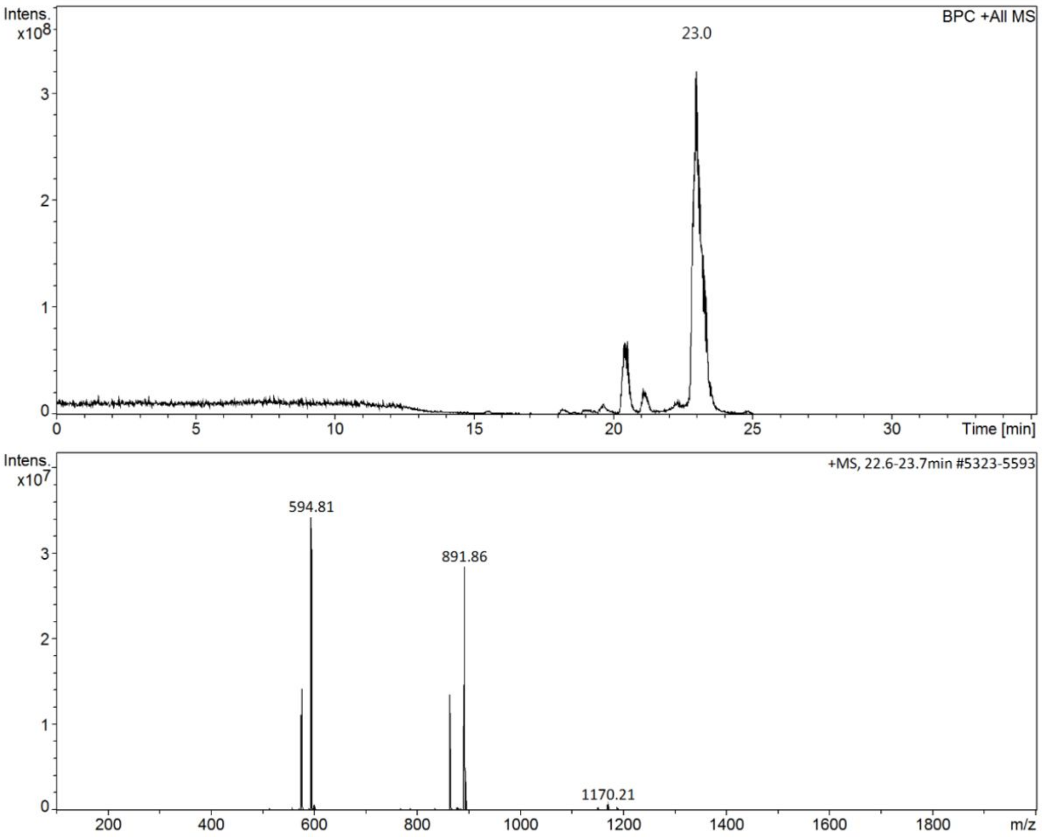

Figure S39: HPLC-MS (SeQuant ZIC-pHILIC, method 1) chromatogram of ^{t}Bu$_4$-C$_4$ZW-DOTA **6d**.

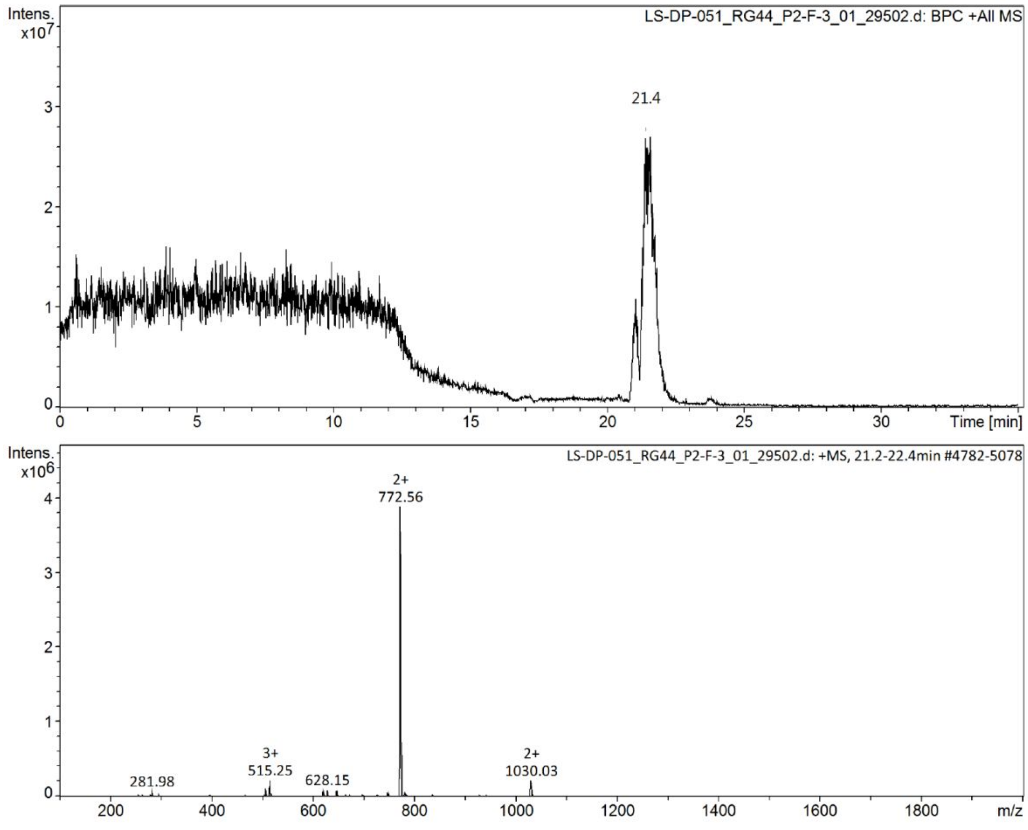

Figure S40: HPLC-MS (SeQuant ZIC-pHILIC, method 1) chromatogram of [Gd-(C$_1$ZW-DOTA)] **7a**.

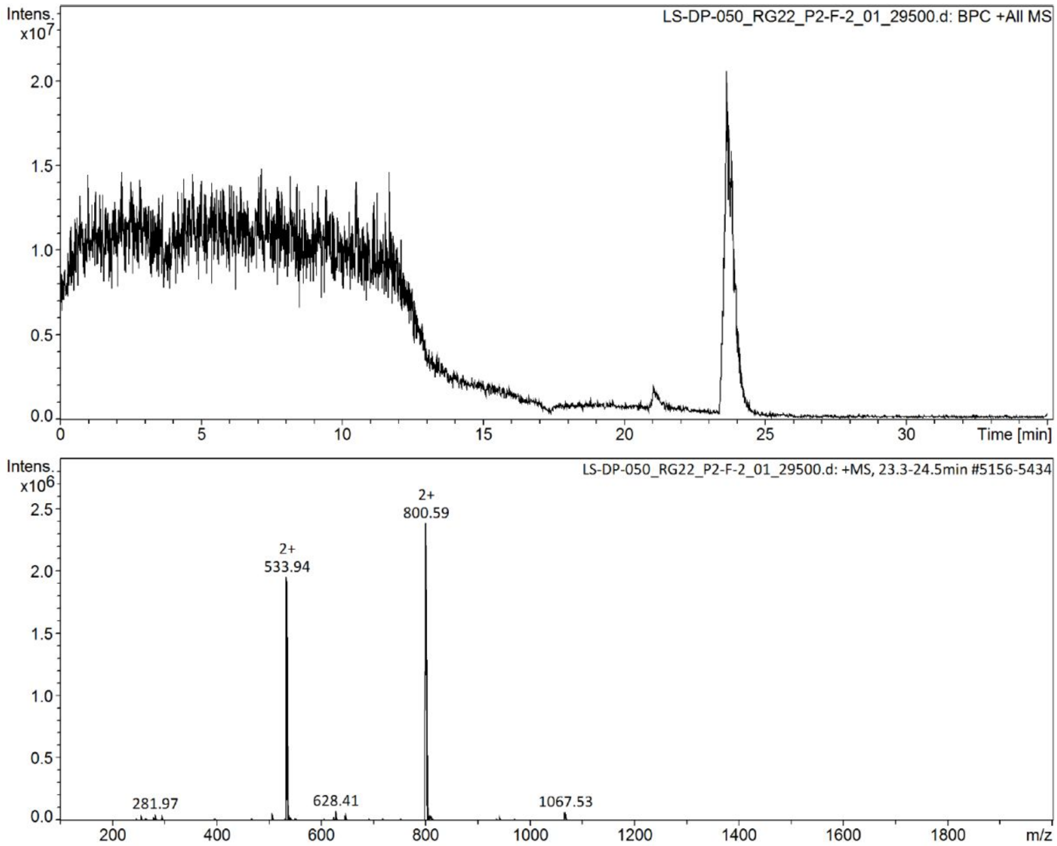

Figure S41: HPLC-MS (SeQuant ZIC-pHILIC, method 1) chromatogram of [Gd-(C_2ZW-DOTA)] **7b**.

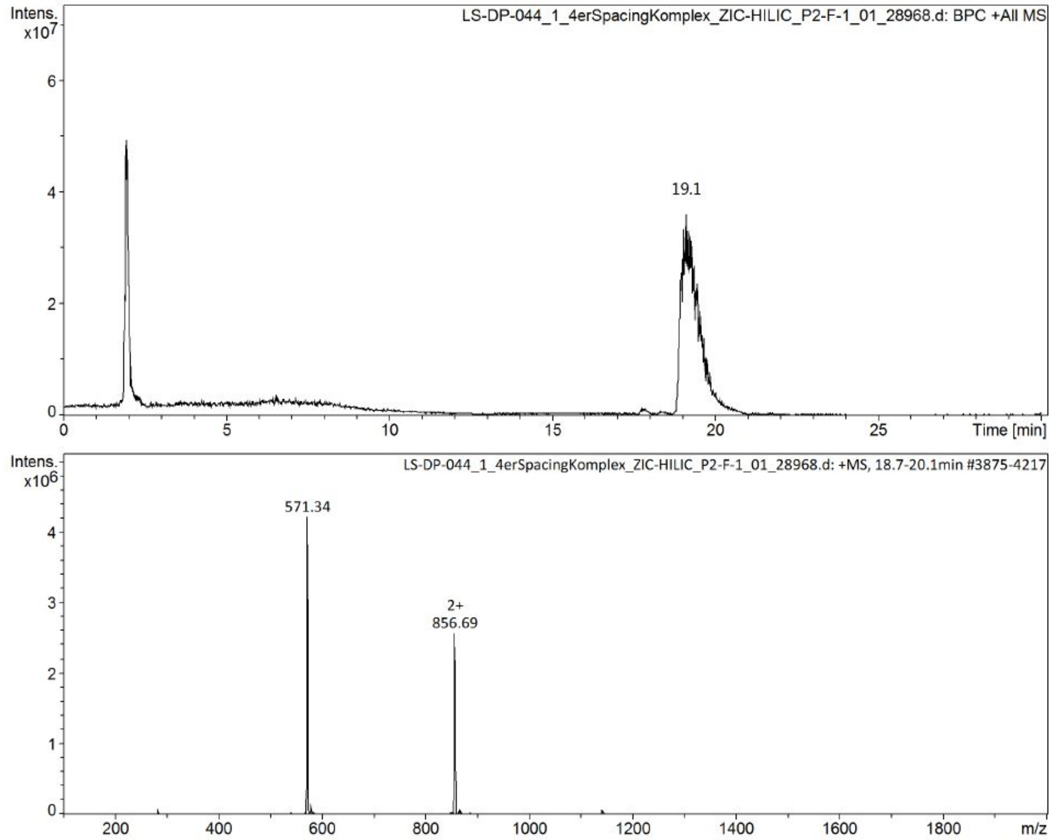

Figure S42: HPLC-MS (SeQuant ZIC-pHILIC, method 1) chromatogram of [Gd-(C_4ZW-DOTA)] **7d**.

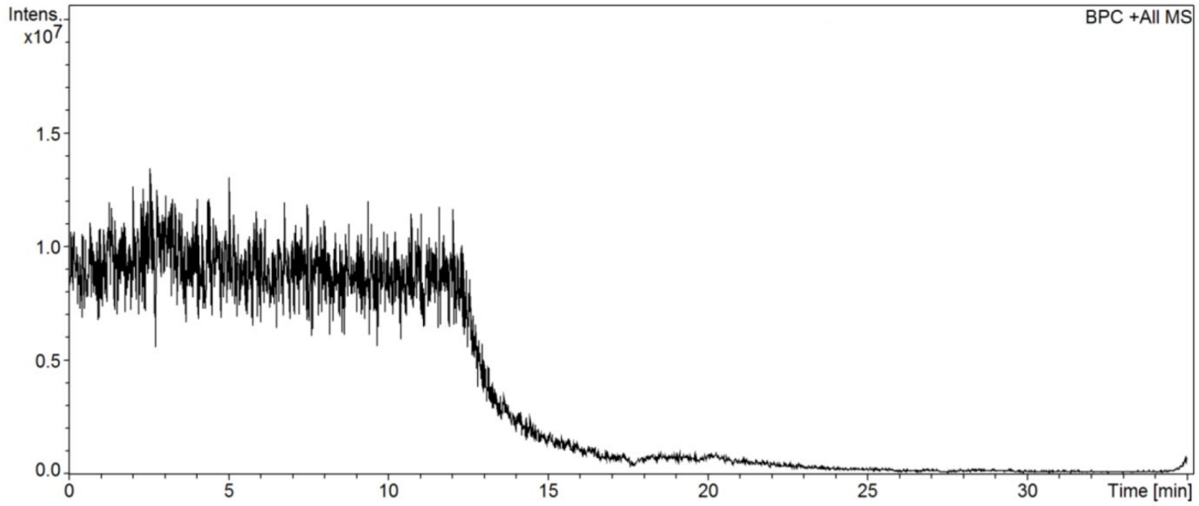

Figure S43: Blank HPLC-MS (SeQuant ZIC-pHILIC, method 1) chromatogram.

Dynamic contrast-enhancement MRI measurements

All animal experiments fitted the guidelines of the Université Laval and Centre de recherche du Centre hospitalier universitaire de Québec's animal ethical committee. Six-week-old BALB/c female mice (Charles River, Montreal, Canada) were first anaesthetized with 3% isoflurane in an induction chamber and transferred to the MRI mouse bed while kept under anesthesia by means of a nose cone integrated to MRI animal bed. The animals were continuously monitored for respiration with a small animal monitoring and gating system (model 1025T; SA Instruments, Stony Brook, NY). For intravenous access, the caudal vein was dilated and cannulated using a 30 G winged needle) connected to a PE-10 catheter (intramedic polyethylene tubing, inner diameter: 280 µm, length: 130 cm, total volume = 80 µL) pre-flushed with heparin and connected to a syringe containing the contrast agent. The needle was secured with adhesive (3M Vetbond) and protective ointment (Lacri-Lube) was applied on the mice's eyes. Then, the animals were positioned within a 35-mm-diameter RF coil and scanned using a 1 T small-animal MRI system (M2M, Aspect Imaging, Israel). The mice were scanned using a T_1-weighted 2D spin−echo sequence in coronal orientation with the following parameters: TR: 700 ms; TE: 14 ms; slice thickness: 0.8 mm; slice gap: 0.1 mm; field-of-view = 100 mm; dwell time = 16 µs; fα = 90°; encoding: 340/200; 1 excitation; total duration = 4 min 16 s. Two pre-injection images were acquired as references (S_0).

Two control animals (mouse 1 = 21.7 g; mouse 2 = 21.0 g) received each 140 µL of a 42 mM gadoteric acid solution prepared by diluting Dotarem 1:12 in saline solution, for a total dose of 5.9 µmol Gd injected per animal. Prior to the injection of each contrast agent, T_1 of the solution was measured using a Bruker Minispec mq60 NMR relaxometer (60 MHz, 1.41 T, 37 °C in saline). For Dotarem the T_1 of the injected solution was 9.577 ± 0.002 ms. In order to generate a similar level of contrast enhancement in the blood of the animals, each injection was then calibrated to reach a T_1 value as close as possible to that obtained for the Dotarem solution (~10 ms). Then, two animals (mouse 3 = 20.2 g, mouse 4 = 24.0 g) were injected with a solution of [Gd-(C_3ZW-DOTA)] **7c** calibrated at T_1 = 10.3 ± 0.1 ms (13.9 mM) with 90 µL (1.25 µmol Gd) in mouse 3 and 100 µL (1.39 µmol) in mouse 4.

MRI scans were acquired approximately every 4 min for the first 90 min p.i., followed by additional acquisitions at 4 h and 24 h p.i..

Regions of interest (ROI) were drawn on MR images (kidney cortex, kidney pelvis, abdominal aorta, brain) using the 3D Slicer imaging software (5.9.0 version). Mean signal values were calculated ($S_{kidney\ cortex}$, $S_{kidney\ pelvis}$, $S_{abdominal}$ S_{aorta}, S_{brain}, respectively) and compared with the mean signal values from adjacent air volumes (S_{air}). Contrast ratios were calculated as follows:

$$Contrast\ ratio\ (CR) = \frac{S_{organ} - S_{air}}{S_{muscle} - S_{air}} \qquad \text{(Equation 1)}$$

Contrast enhancement at time points was calculated as follows:

$$Contrast\ enhancement\ (CE) = \frac{CR_{t0}}{CR_{tx}} \qquad \text{(Equation 2)}$$

Where CR_{t0} and CR_{tx} are contrast ratios at times t_0 (prior to injection) and t_x.

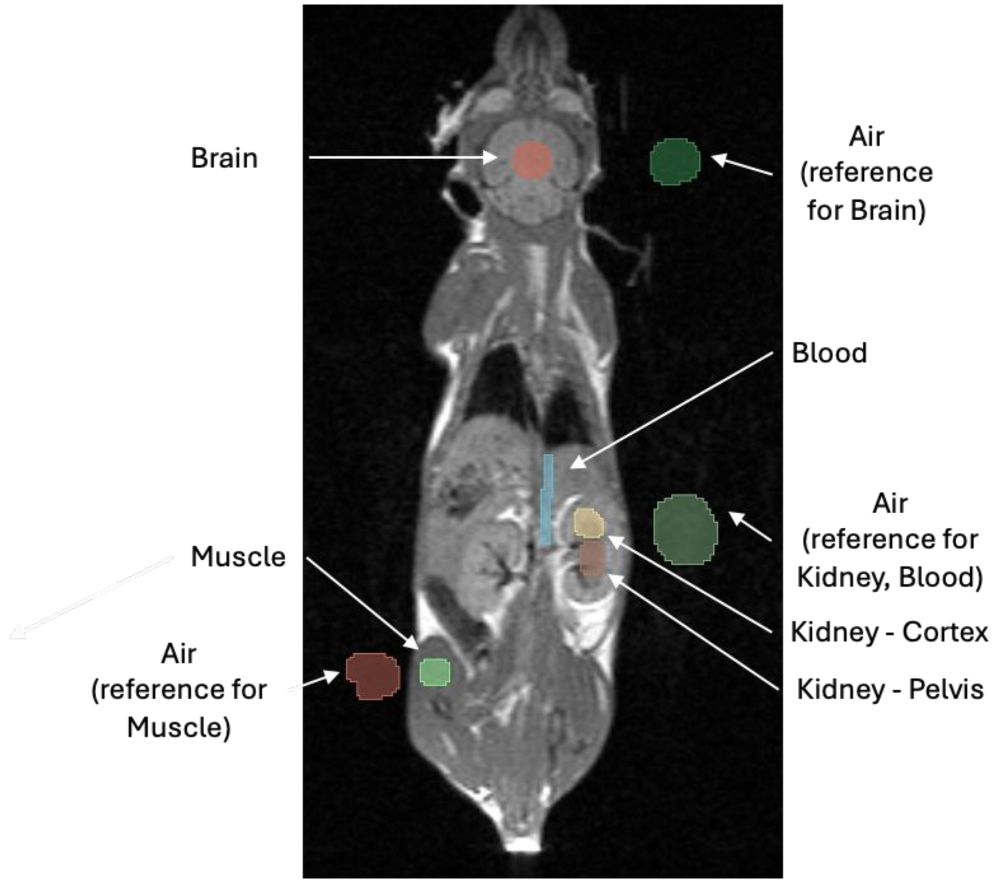

Figure S44: Defined regions of interest used for the calculation of the relative CE ratios.

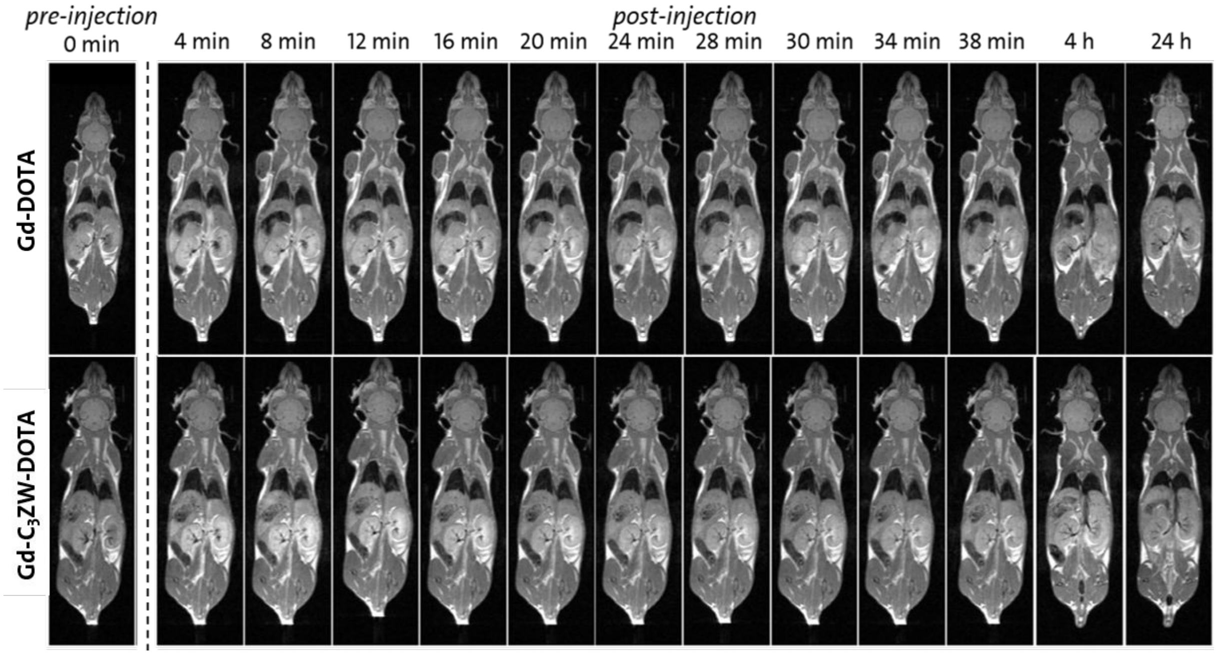

Figure S45: Dynamic contrast-enhanced MRI following GBCA administration. Sequential T_1-weighted images acquired from 0 min (pre-injection) up to 24 h post-injection following administration of [Gd-(DOTA)] (Dotarem) and [Gd-(C_3ZW-DOTA)] **7c**. Images illustrate the time-dependent evolution of contrast enhancement and clearance dynamics of the zwitterionic GBCAs in comparison to the non-zwitterionic reference.
